# Respiratory Syncytial Virus Two-Step Infection Screen Reveals Inhibitors of Early and Late Life Cycle Stages

**DOI:** 10.1128/aac.01032-22

**Published:** 2022-11-08

**Authors:** Svenja M. Sake, Christina Kosch, Sebastian Blockus, Sibylle Haid, Antonia P. Gunesch, Xiaoyu Zhang, Martina Friesland, Sofie B. Trummer, Christina Grethe, Anne Kühnel, Jessica Rückert, W. Paul Duprex, Jiabin Huang, Marie-Anne Rameix-Welti, Martin Empting, Nicole Fischer, Anna K. H. Hirsch, Thomas F. Schulz, Thomas Pietschmann

**Affiliations:** a Institute for Experimental Virology, Twincore-Centre for Experimental and Clinical Infection Research, Hanover, Germany; b Helmholtz Institute for Pharmaceutical Research Saarland (HIPS), Helmholtz Centre for Infection Research (HZI), Saarbrücken, Germany; c Department of Pharmacy, Saarland University, Saarbrücken, Germany; d Institute of Virology, Hanover Medical School, Hanover, Germany; e German Centre for Infection Research, Hanover-Braunschweig Site, Hanover, Germany; f Centre for Vaccine Research, University of Pittsburgh, Pittsburgh, Pennsylvania, USA; g Institute for Medical Microbiology, Virology and Hygiene, University Medical Center Hamburg-Eppendorf, Hamburg, Germany; h Université Paris-Saclay, Université de Versailles St. Quentin, UMR 1173 (2I), INSERM, Assistance Publique des Hôpitaux de Paris, Hôpital Ambroise Paré, Laboratoire de Microbiologie, Versailles, France; i Helmholtz International Lab for Anti-Infectives, Helmholtz Center for Infection Research, Braunschweig, Germany; j Cluster of Excellence RESIST (EXC 2155), Hanover Medical School, Hanover, Germany

**Keywords:** RSV, antivirals, drug discovery, drug screen

## Abstract

Human respiratory syncytial virus (hRSV) infection is a leading cause of severe respiratory tract infections. Effective, directly acting antivirals against hRSV are not available. We aimed to discover new and chemically diverse candidates to enrich the hRSV drug development pipeline. We used a two-step screen that interrogates compound efficacy after primary infection and a consecutive virus passaging. We resynthesized selected hit molecules and profiled their activities with hRSV lentiviral pseudotype cell entry, replicon, and time-of-addition assays. The breadth of antiviral activity was tested against recent RSV clinical strains and human coronavirus (hCoV-229E), and in pseudotype-based entry assays with non-RSV viruses. Screening 6,048 molecules, we identified 23 primary candidates, of which 13 preferentially scored in the first and 10 in the second rounds of infection, respectively. Two of these molecules inhibited hRSV cell entry and selected for F protein resistance within the fusion peptide. One molecule inhibited transcription/replication in hRSV replicon assays, did not select for phenotypic hRSV resistance and was active against non-hRSV viruses, including hCoV-229E. One compound, identified in the second round of infection, did not measurably inhibit hRSV cell entry or replication/transcription. It selected for two coding mutations in the G protein and was highly active in differentiated BCi-NS1.1 lung cells. In conclusion, we identified four new hRSV inhibitor candidates with different modes of action. Our findings build an interesting platform for medicinal chemistry-guided derivatization approaches followed by deeper phenotypical characterization *in vitro* and *in vivo* with the aim of developing highly potent hRSV drugs.

## INTRODUCTION

Human respiratory syncytial virus (hRSV) is the most common cause of acute lower respiratory tract infections in infants and the most frequent cause of hospitalization among those younger than one year (1, 2). Although hRSV infection is usually mild in healthy juvenile and adult populations, it causes substantial disease burden among patients with immune suppression and the elderly. Considering industrialized countries alone, RSV infection caused around 336,000 hospital admissions and 14,000 hospital deaths among older adults (≥65 years) in 2015 (3). Therefore, RSV causes substantial global disease burden in different age groups and patient populations.

A prophylactic monoclonal antibody (Palivizumab) is licensed, and vaccine candidates are in clinical development (4, 5). However, therapeutic options remain limited, and patients are largely treated with supportive care.

The nucleoside analogue Ribavirin inhibits RSV-infection *in vitro* and is approved for RSV therapy. Nevertheless, due to adverse side effects and conflicting evidence on its efficacy, its use is no longer recommended (6, 7). Numerous antiviral strategies against RSV are in preclinical or clinical development (8–10). These include molecules which target distinct viral proteins such as glycoprotein G, fusion protein F, nucleocapsid protein N, transcriptional regulator protein M2-1, and viral RNA-dependent RNA polymerase L (8–11).

The largest number of directly acting RSV antivirals and the most clinically advanced ones target the RSV F protein. However, F protein inhibitors rapidly select for viral resistance (12, 13). The development of Presatovir, for example, has been terminated because it did not meet its clinical trial endpoints (9). Among the non-F-targeting antivirals, the development of Lumicitabine (ALS-008176) (14), a nucleosidic polymerase inhibitor, was also suspended (9). To identify new molecules with diverse modes of action as anchor points for RSV drug development, we conducted a phenotypic screen interrogating a small molecule compound library across the entire RSV replication cycle.

## RESULTS

**FIG 1:**
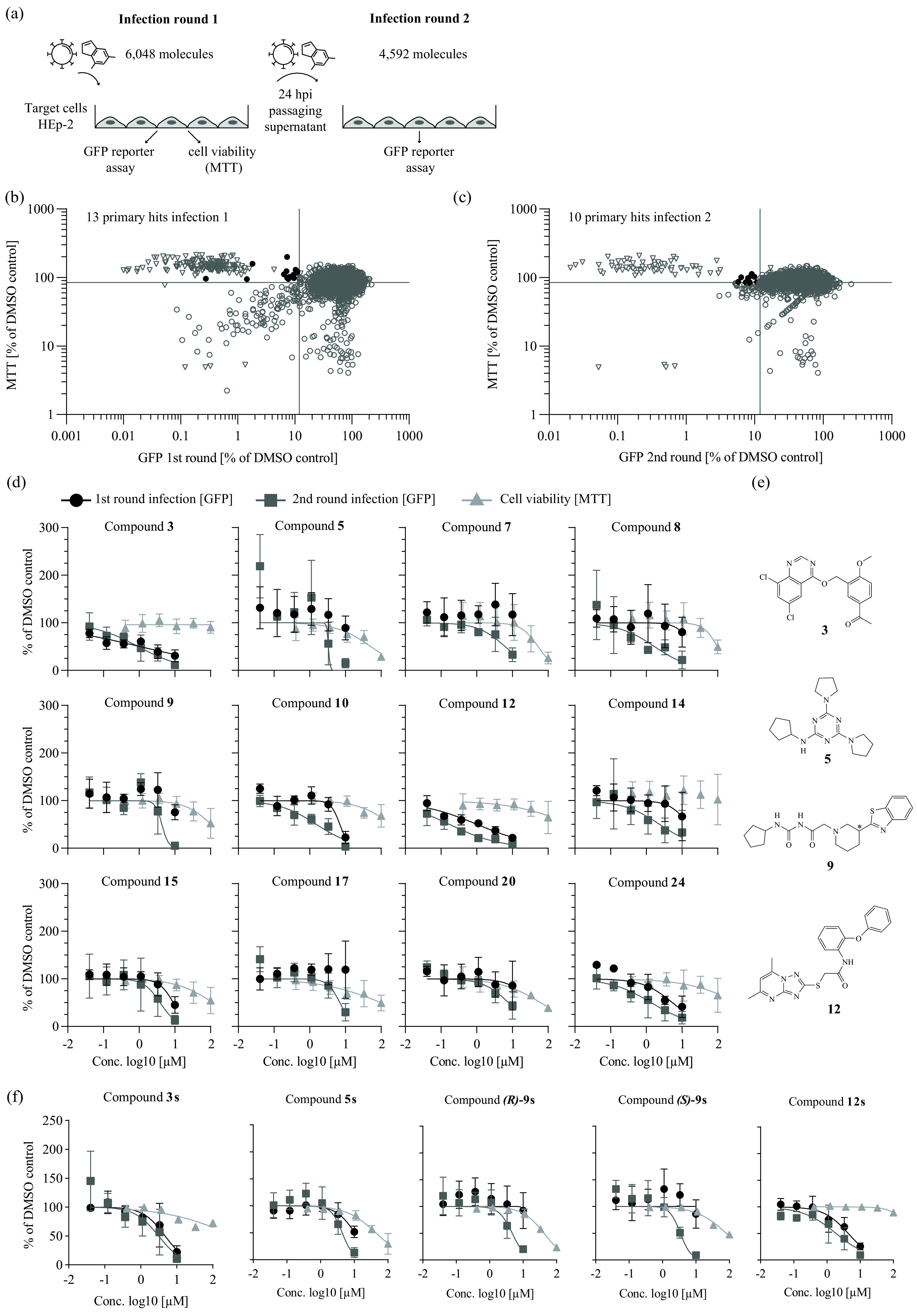
Two-step screening of a small molecule compound library against human respiratory syncytial virus (hRSV). (a) Schematic representation of two-step infection screen of hRSV B05 enhanced green fluorescent protein (eGFP) in HEp-2 cells. (b) Primary hit candidates from the first round of infection. Each circle represents the mean value of four technical replicates. Black circles represent hit compounds according to hit criteria of MTT [3-(4,5-dimethyl-2-thiazolyl)-2,5-diphenyl-2H-tetrazolium bromide] value (cell viability) ≥ 85% and eGFP fluorescence (infection efficiency) ≤ 12%. Synagis (2 μg/mL) was used as a positive control (empty triangles). Cell viability and infection efficiency are plotted relative to the dimethyl sulfoxide (DMSO)-treated control infections, which were set to 100%. (c) Hit compounds in second-round infection assay prioritizing for late viral replication stages (of compounds showing >60% eGFP signal in first round). Cutoff criteria for hit compounds were set at MTT ≥ 85% (round 1) and eGFP fluorescence ≤ 12% (round 2). (d) Dose-response curves of selected hit candidates against a firefly luciferase-expressing hRSV A strain Long reporter virus (rHRSV-A-Luc) (15). Black circles represent infection round 1, gray squares represent infection round 2. For both round 1 and 2 infection assays, luciferase activity was assessed 24 h postinoculation and normalized to control infections conducted in the presence of DMSO. An MTT assay was performed on uninfected cells treated with the given compound doses for 24 h (filled triangles). Means and standard deviations (SD) from three independent experiments are shown. (e) Chemical structure of primary candidate molecules. Compound 9: no stereo information available. (f) Dose-response activity of given resynthesized compounds. Assay setup as in panel d.

We used a two-step screen of an in-house library of small molecules assembled from commercially available libraries (see Materials and Methods) to discover novel hRSV inhibitors with diverse modes of action. To separate cytotoxic compounds from molecules which directly inhibit hRSV, we also conducted a reading of cell viability after the first round of infection (Fig. 1a). We analyzed 6,048 molecules in total and performed a second round of infection for only 4,592 compounds due to technical issues leading to a loss of compounds. We identified 16 candidates which met our first round hit-calling criteria (HRSV-B05 enhanced green fluorescent protein [eGFP] ≤ 12% and 3-(4,5-dimethyl-2-thiazolyl)-2,5-diphenyl-2H-tetrazolium bromide [MTT] ≥ 85% of dimethyl sulfoxide [DMSO] control; Fig. 1b) and selected 10 compounds which complied with our second round hit criteria (first round, rHRSV-B05 eGFP ≥ 60% and MTT ≥ 85%; second round, rHRSV-B05 eGFP ≤ 12%; Fig. 1c).

For 23 of these primary hits, we conducted an orthogonal hRSV luciferase reporter virus infection assay (rHRSV-A-Luc) (15) to confirm their activity. Eleven compounds showed no dose-dependent antiviral activity against hRSV-A-Luc (data not shown) and were excluded. The remaining compounds showed a 50% inhibitory concentration (IC_50_) of ≤10 μm (Fig. 1d, Table S1 in the supplemental material). Compounds 3 and 12 in particular reduced infection with broad therapeutic windows in rounds I and II (Fig. 1d, Table S1), whereas molecules 5, 7, 9, 17, and 20 preferentially inhibited the second round of infection. To exclude library artifacts, we analyzed the purity of the most interesting hits, 3, 5, 9 and 12, via liquid chromatography-mass spectrometry (Table S2), resynthesized them, and confirmed their activity (Fig. 1f). The chemical structures of these molecules are shown in Fig. 1e. For further experiments, we used compounds at their round I IC_90_ concentrations but at a maximum of 1/3 of the 50% cytotoxic concentration (CC_50_) to avoid indirect effects due to cytotoxicity. In cases where either the value was not calculable or the resulting concentration would have exceeded 100 μm, compounds were used at their IC_90_ round II concentrations (Table S1).

**FIG 2:**
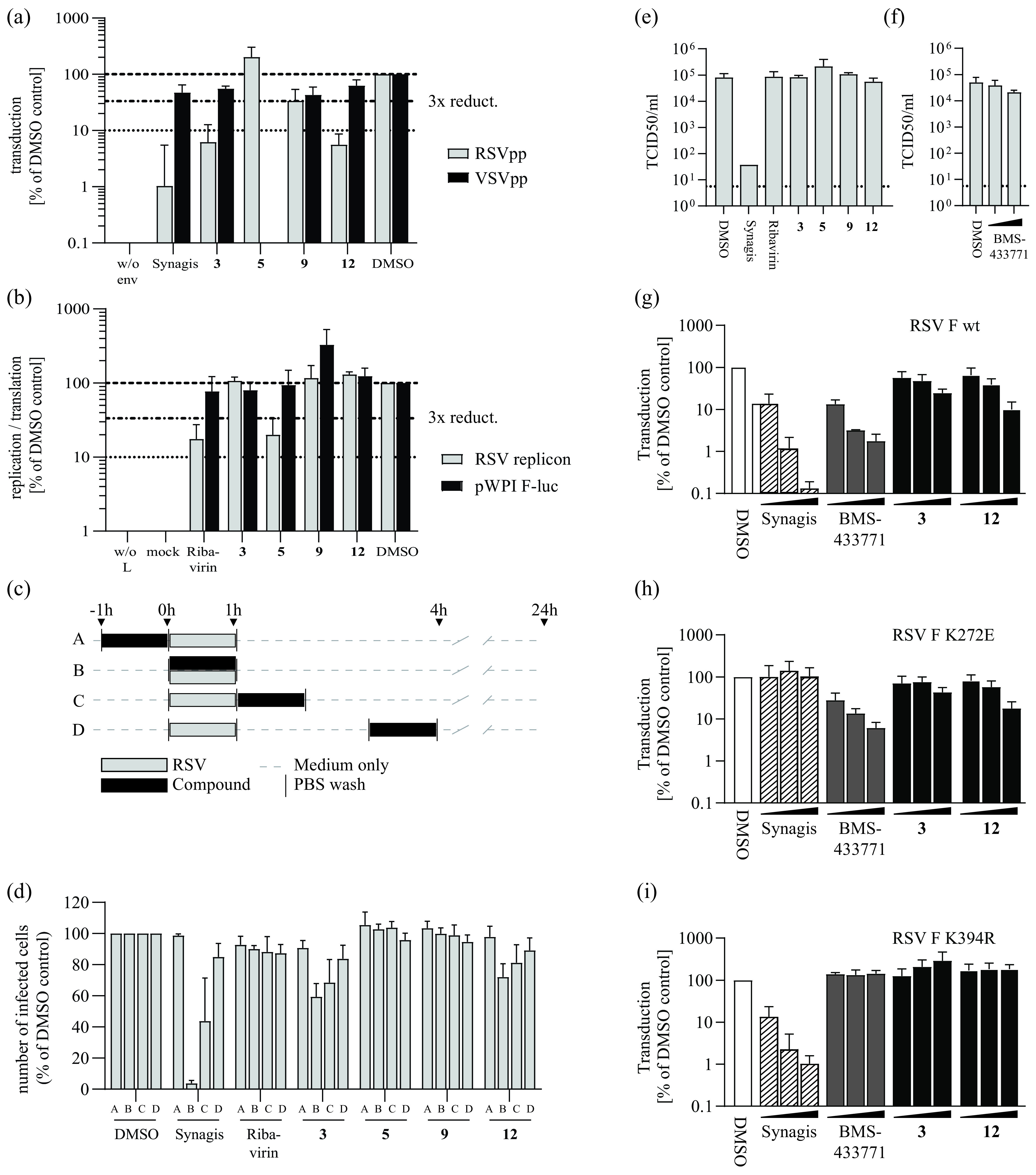
Primary hit candidates target cell entry or late stages in hRSV infection. (a) Inhibition of hRSV-F mediated cell entry in the lentiviral pseudotype asssay. Values were normalized to DMSO-treated infected wells. Synagis (10 μg/mL) was used as control for hRSV-F mediated cell entry inhibition. The compound concentrations used are summarized in Table S1. Means and SD of three independent experiments are shown. (b) Activity of hit compounds against hRSV replication/transcription as determined by an hRSV replicon assay in BSR T7/5 cells. Cells were transfected with the RSV replicon and helper plasmids or with a firefly luciferase-expressing control plasmid (pWpi F-luc). Normalized means and SD of three independent replicates are shown. (c) Schematic representation of the time-of-addition infection assay conducted as outlined above (a, b, c, d). Cells were infected with the recombinant hRSV B05 eGFP (29) reporter virus for 1 h. (d) Results from the time-of-addition infection assay. The number of eGFP-positive, infected cells was assessed by flow cytometry 24 h after inoculation and is normalized to the DMSO control infections. Mean values and SD from three independent replicates are shown. (e and f) Limiting dilution infection assay. HRSV was incubated with 100 μm of given compounds or Synagis (100 μg/mL) at 1 h before serial dilution and infection of HEp-2 cells. Cells were fixed and stained for hRSV phosphoprotein. The 50% tissue culture infective dose (TCID_50_)/mL was calculated from the number of infected wells. Mean and SD from two to three independent replicates are shown. (g, h, i) Impact of hRSV-F protein resistance to Synagis (K272E) (h) or fusion inhibitors (K394R) (i) on the antiviral potency of selected compounds. HEp-2 cells were transduced with lentiviral pseudoviruses harboring WT F protein (g) (RSV F wt) or given resistance mutations (h, i). Increasing concentrations of the indicated compounds (black bars) were applied simultaneously. At 72 h postransduction, luciferase activity was quantified and normalized to the DMSO control (white bars). Synagis: 0.1, 1.0, and 10.0 μg/mL; Compounds 3 and 12: 0.1, 1.0, and 10.0 μm. Means and SD from three independent replicates are shown.

Next, we used lentiviral pseudotypes (16) to analyze inhibition of hRSV cell entry (Fig. 2a). Compounds 3 and 12 reduced transduction of target cells with hRSV pseudoparticles by ca. 20-fold but did not inhibit lentiviral VSV-G pseudoparticles, indicating an RSV glycoprotein-dependent entry inhibition (Fig. 2a). Compound 9 was slightly active against both types of pseudoparticles, and compound 5 blocked VSV-G, but not hRSV-F-mediated cell entry (Fig. 2a).

In the minigenome replicon assay, only compound 5 showed inhibition of hRSV replication and/or transcription (Fig. 2b). These data suggested that compounds 3 and 12 inhibit RSV cell entry, compound 5 impairs RSV transcription/RNA replication, and compound 9 impedes late life cycle stages.

To corroborate these findings, we conducted time-of-addition assays with an RSV-A strain Long GFP reporter virus (rHRSV-A-GFP) (17) (Fig. 2c and d). Compounds 3 and 12 led to approximately 40% and 30% reduction of hRSV infection when present during virus inoculation, and their effect decreased when treatment was initiated at later time points (Fig. 2d). Their patterns of inhibition matched that of the neutralizing antibody Synagis (Palivizumab, Fig. 2d). Comparable to the replication inhibitor Ribavirin, compounds 5 and 9 had no antiviral effect when present for 1 h during any of these early time points (Fig. 2d).

We next investigated whether the compounds exhibited anti-hRSV activity through direct binding to or disruption of authentic virus particles. To this end, we used stocks of a recent clinical isolate, hRSV-A-ON1-H1 (18) (Fig. 2e and f). When the hRSV-F binding antibody Synagis was co-incubated with virus particles and then diluted, it retained its antiviral activity, leading to a lower virus titer in the 50% tissue culture infective does (TCID_50_) measurements (Fig. 2e). As expected, Ribavirin was diluted out, leaving the virus infection titer unchanged (Fig. 2e). Likewise, the tested small molecules and BMS-433771, a small molecule hRSV inhibitor known to bind to the F protein, did not reduce the infection titer (Fig. 2e and f). This refutes the possibility that these compounds disrupt virus particles or that their mode of action is mediated by irreversibly binding to them.

Typically, entry inhibitors targeting hRSV-F select for viral resistance (12, 13, 19). To test whether the antiviral activity of compounds 3 and 12 is influenced by a classical hRSV-F protein resistance mutation against small molecule fusion inhibitors such as K394R (20, 21) or a resistance mutation to Synagis (K272E) (22), we conducted hRSV-F pseudotype assays with particles carrying either wild-type hRSV-F (Fig. 2g) or variant hRSV-F proteins with either the K272E (Fig. 2h) or the K394R (Fig. 2i) mutation. Synagis and BMS-433771 inhibitors were used as controls in these assays. The Synagis resistance mutation K272E did not ablate the activity of compounds 3 and 12 (Fig. 2h), whereas the K394R exchange abrogated the effect of both compounds 3 and 12 as well as that of the BMS-433771 control (Fig. 2g). These results supported the conclusion that compounds 3 and 12 inhibit hRSV-F mediated cell entry by targeting the hRSV-F protein.

**FIG 3:**
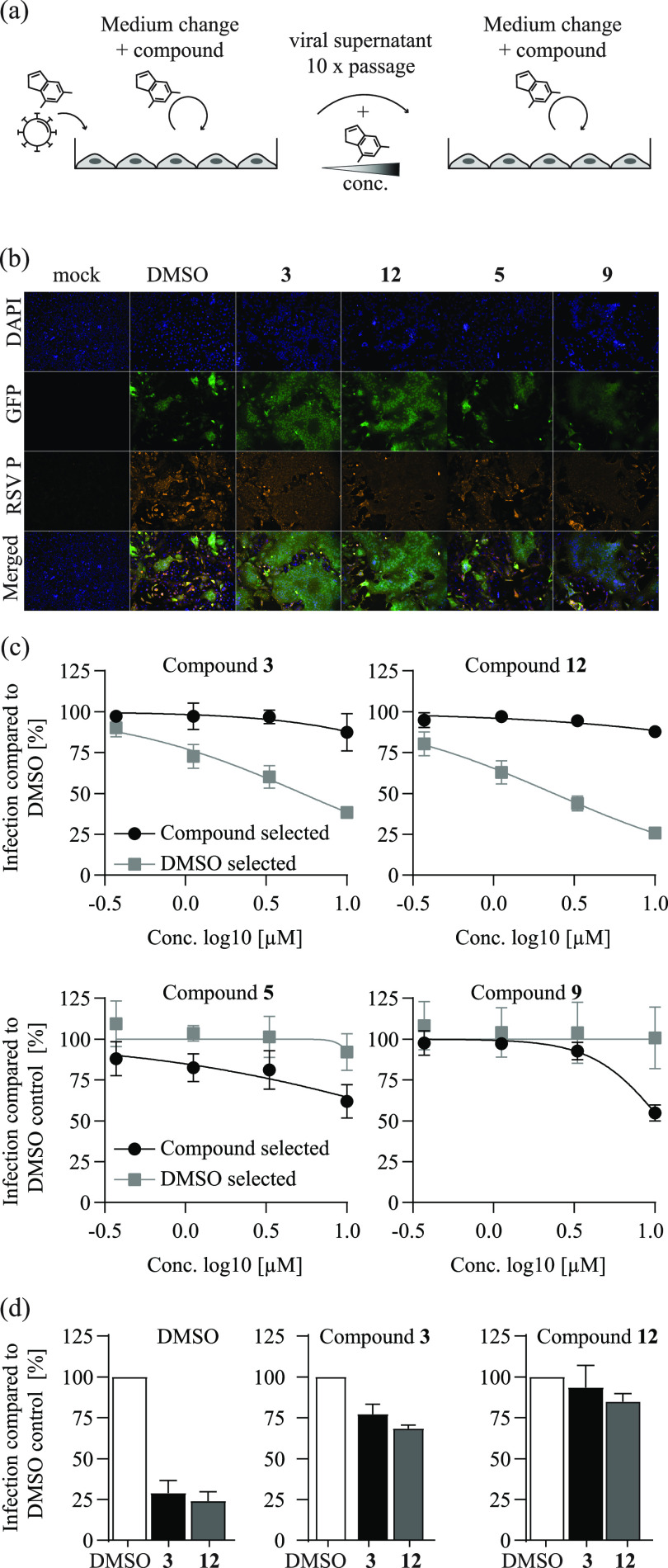
Serial passaging of hRSV induces resistance against selected compounds. (a) Schematic representations of hRSV resistance selection assay. rHRSV-A-GFP, an hRSV A strain Long reporter virus (17), was passaged in the presence of selected hit compounds under increasing concentrations for 10 rounds. In parallel, rHRSV-A-GFP was passaged in the presence of DMSO. (b) Fluorescence microscopy analysis of compound-selected rHRSV-A-GFP infected HEp-2 cells. Cells were inoculated with passage 10 rHRSV-A-GFP virus populations and treated with the compound concentrations present during the final passage (Table S3). At 48 h after virus inoculation, cells were fixed, permeabilized, and stained for hRSV phosphoprotein. Blue, DAPI (4′,6-diamidino-2-phenylindole); orange, hRSV P protein; green, GFP reporter. (c) Phenotypic resistance analysis of virus populations selected with compounds. HEp-2 cells were infected with DMSO- or compound-selected virus populations and treated with the respective compound as given above in each panel. At 24 h postinoculation, cells were fixed, and the number of GFP-positive cells was quantified by fluorescence-activated cell sorter (FACS) and normalized to that observed with DMSO control infections of these virus populations. Gray, dose-response of DMSO-passaged virus population. Black: dose-response of virus population selected with the compound listed at the top of each panel. Mean values and SD of three independent experiments are shown. (d) Cross-resistance of compound 3- and 12-selected rHRSV-A-GFP virus populations. HEp-2 cells were infected with the DMSO-selected (left) compound 3-selected (middle), or compound 12-selected (right) virus populations in the presence of 100 μM compound 3 (black bars) or compound 12 (gray bars). At 24 h postinfection, cells were fixed, and the number of GFP-positive cells was quantified by FACS and normalized to the respective infection in the presence of DMSO. Mean and SD of three independent experiments are shown.

Next, we cultured rHRSV-A-GFP viruses in the presence of increasing doses of compounds 3, 12, 5, and 9 over ten consecutive virus passages (Table S3). As a control, we also passaged rHRSV-A-GFP viruses in the presence of DMSO (Fig. 3a). We examined phenotypic changes in these passaged virus populations by fluorescence microscopy of infected HEp-2 cells (Fig. 3b) and by dose titration of the compounds on the respective passaged virus populations (Fig. 3c). Culturing of rHRSV-A-GFP viruses with compounds 3 and 12—as with other hRSV fusion inhibitors (21)—selected for virus populations with enhanced syncytium formation compared to DMSO- or compound 5- and 9-selected viruses (Fig. 3b). Unlike compounds 5 and 9, compounds 3 and 12 selected for viruses with resistance against the cognate compound and vice versa (Fig. 3c and d).

**FIG 4:**
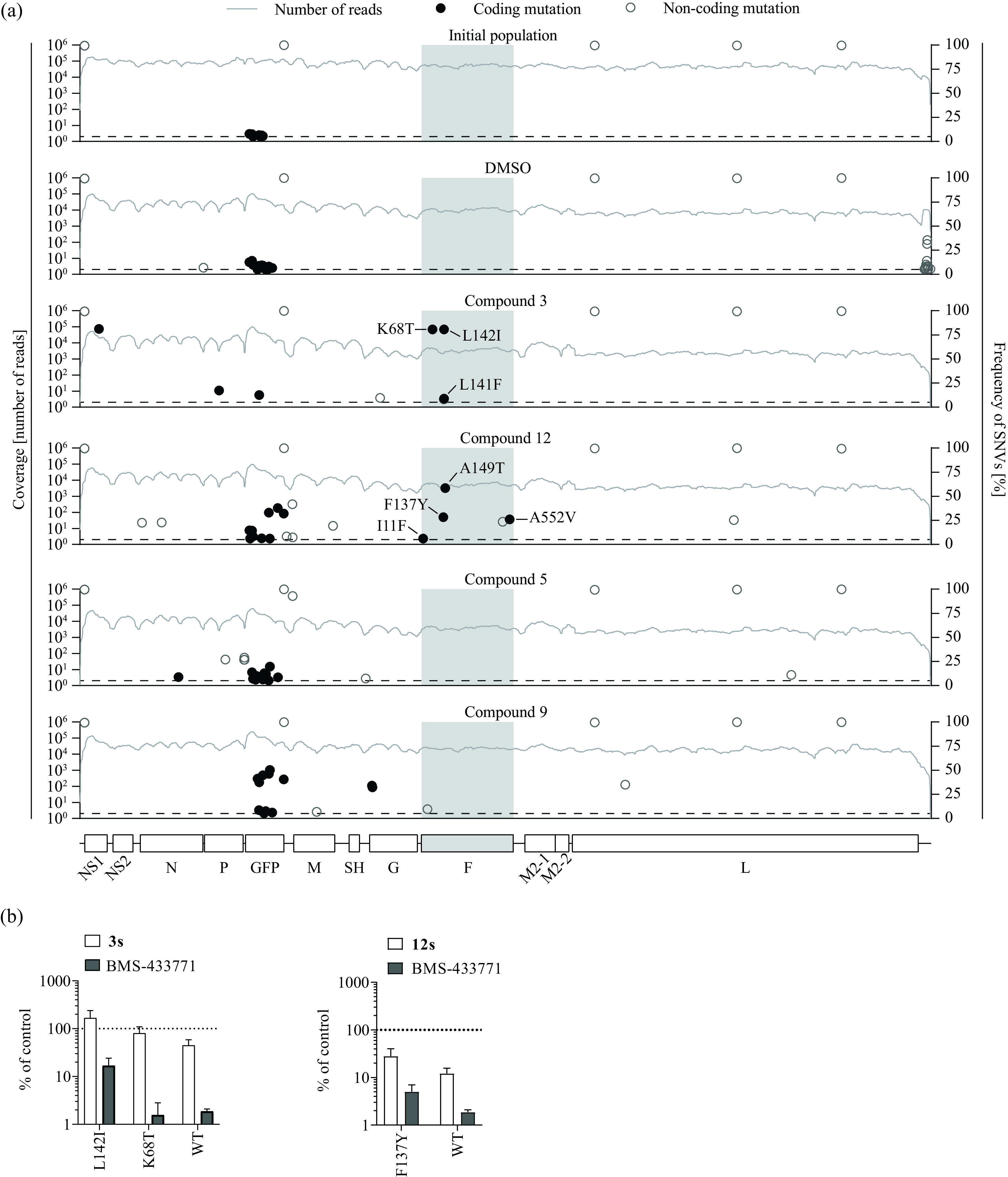
Sequence analysis of compound-selected virus populations and RSV F protein cell entry resistance. (a) Virus populations derived from the initial rHRSV-GFP reporter virus after 10 passages in the presence of DMSO or the given compounds were analyzed by Illumina sequencing. Coding (black circles) and noncoding (open circles) single nucleotide variants (SNVs) were detected based on comparing the sequence reads with the initial plasmid DNA sequence. The frequency of each variant is plotted, and each variant is shown at its respective position in the RSV genome. Lines depict the number of reads at the indicated genome position. Amino acid exchanges with a frequency of ≥5% (dotted line) are labeled. The coding region of the F protein is highlighted with gray shading. (b) Lentiviral hRSV F protein pseudoparticle resistance assay. Lentiviral particles with hRSV F proteins carrying the wild-type protein (WT) or F protein variants with the K68T, L142I, or F137Y mutation, respectively, were used to transduce Huh-7.5 cells in the presence of compound 3 (25 μM), compound 12 (64 μM) (white bars, respectively), or BMS-433771 (10 μM, gray bars). At 72 h postransduction, luciferase activity was quantified and normalized to the DMSO control. Mean values and SDs from three independent replicates are shown.

To examine whether these viral phenotypic changes correlated with fixation of coding mutations in any viral protein, we sequenced the passaged virus populations (Fig. 4a). Similar to the initial virus population, the DMSO-passaged viruses exhibited only a few low-abundance coding variants within the GFP-coding and non-coding variants which distinguished rHRSV-A-GFP from the wild-type RSV strain Long sequence. In contrast, viruses passaged in the presence of compounds 3 and 12 accumulated three and four coding changes, respectively, within the F protein (Table S4). In addition, compound 3 selected a mutation in nonstructural protein 1 (NS1) that was highly frequent in the population and a mutation within the phosphoprotein (P). In contrast, compound 5 did not select for any F protein mutations, and compound 9 selected for only one N protein change or two G protein exchanges. To confirm that the most prominent F protein mutations selected for by compounds 3 or 12 conferred resistance, we tested them in the RSV pseudotype system (Fig. 4b). Both the L142I mutation and the F137Y mutation conferred partial resistance to BMS-433771 and the cognate compounds that had selected them. Both mutations map to the fusion peptide region of the RSV F protein, a hot spot for accumulation of fusion inhibitor resistance mutations (19). These data support the conclusion that compounds 3 and 12 are F protein-binding, hRSV-F-mediated cell entry inhibitors. Because virus populations selected in the presence of compounds 5 and 9 did not show phenotypic resistance, we did not follow up the coding mutations which had accumulated at low abundance.

**FIG 5:**
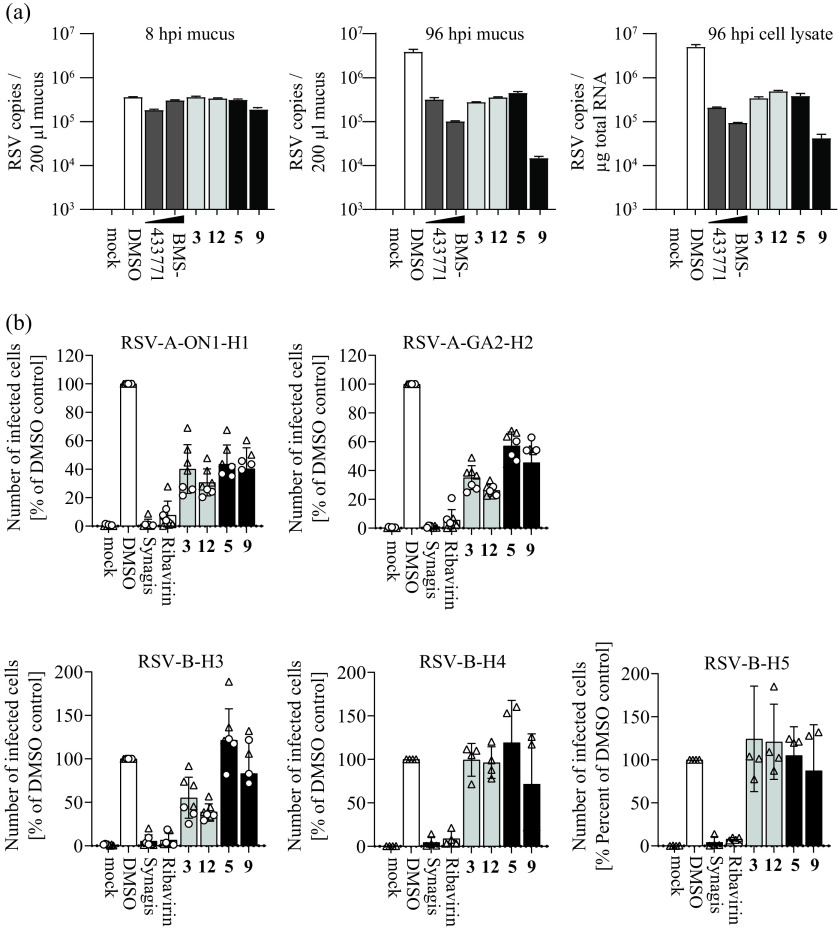
Anti-hRSV activity of selected hit compounds in air-liquid interface cultures of immortalized BCi-NS1.1 cells and RSV-strain dependence of antiviral activity. (a) Anti-hRSV activity of selected hit compounds was assessed in differentiated BCi-NS1.1 cells (23) cultured in an air-liquid interface configuration. Cells were treated from the basolateral side with compounds (concentrations used are summarized in Table S1; 2 and 0.2 μM for BMS-433771) and simultaneously infected with the recombinant rHRSV-A-GFP reporter virus. Basolateral treatment was repeated once daily. Apical washes were collected, and cells were lysed for determination of viral genome copies by reverse-transcription quantitative PCR (qRT-PCR). Genome copies in the mucus after 8 and 96 h and in cell lysate (96 hpi) are shown. Mean values and SD from a single experiment with duplicate measurements are depicted. (b) RSV strain-dependence of the antiviral activity of selected hit compounds. Clinical isolates of RSV-A and RSV-B were used to infect HEp-2 cells (multiplicity of infection [MOI] of 1 or 0.5; dots and triangles, respectively) in the presence of compounds (concentrations as in panel a). After 24 h, infection efficiency was determined by intracellular hRSV phosphoprotein staining and flow cytometry. Bars represent the mean values. Means and SDs are given. Four to seven independent replicates are shown.

Next, we validated the activity of these compounds in a more authentic cell culture system and across a broader range of recent clinical RSV strains. To this end, we infected differentiated BCi-NS1.1 cells (23) grown in an air-liquid interface configuration with hRSV and treated them basolaterally with the selected compounds (Fig. 5a). At 96 hours postinfection (hpi), compounds 3, 12, and 5 reduced RSV copy numbers in the mucus and extracts of infected cells by more than 10-fold, whereas compound 9 inhibited accumulation of viral RNA by ca. 100-fold. To test the breadth of antiviral activity, we used two different recent primary RSV A strains representing the ON1 or GA2 types and three distinct RSV B isolates. We infected HEp-2 cells at a multiplicity of infection (MOI) of 1 or 0.5 and quantified infection by intracellular staining of the hRSV phosphoprotein and flow cytometry 24 h later. Compounds 3 and 12 reduced infection by both RSV A isolates and one of the RSV B isolates (Fig. 5b). Compound 5 was not active against the RSV B isolates and only inhibited infection by the RSV A strains. Likewise, compound 9 primarily inhibited the RSV A strains and had low and variable activity against the RSV B viruses. Collectively, these results confirm antiviral activity in a differentiated lung cell model and reveal a strain-dependent effect of these molecules.

**FIG 6:**
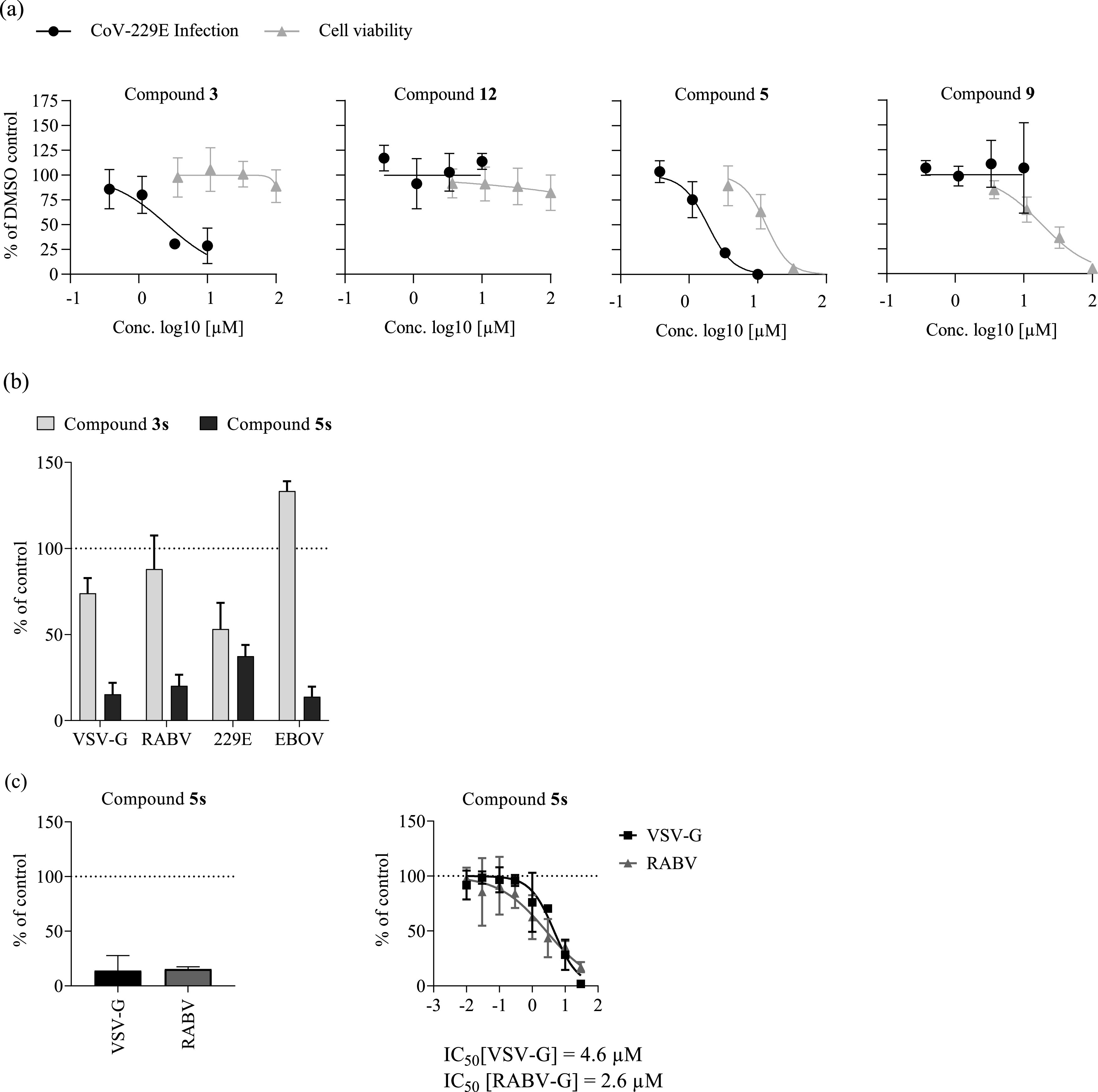
Broad-spectrum antiviral activity of compound 5s. (a) Huh-7.5 cells were infected with a renilla luciferase expressing human coronavirus (hCoV-229E)-Rluc reporter virus (38) in the presence of compounds at the indicated concentrations for 48 h. The cytotoxicity of these compounds was assessed by MTT assays of uninfected Huh-7.5 cells exposed to the compounds for 48 h. Values were normalized to the DMSO control and averages of three independent replicates ± SD are shown. (b) Huh-7.5_ACE2 cells were transduced with recombinant vesicular stomatitis virus (VSV)-based pseudoparticles encoding firefly luciferase and resynthesized compounds 3s (25 μM) and 5s (17 μM) or DMSO. After 4 h postransduction, medium was changed, and luciferase activity was determined after 16 to 18 h as a measure of residual transduction. (c) Huh-7.5 cells were seeded in 96-well plates and inoculated with lentiviral pseudoparticles harboring the G proteins of VSV (black bar) or rabies virus (RABV; gray bar and compound 5s (17 μM), or DMSO and inoculum was removed after 16 h. Firefly luciferase activity was determined after another 72 h. Values of three independent biological replicates were normalized to their DMSO control and means and SD are shown. EBOV, ebola virus.

Given the RSV strain-dependent activities and differential modes of action of these compounds, we explored whether they inhibit a human-pathogenic coronavirus. Interestingly, both compounds 3 and 5 inhibited human coronavirus (hCoV-229E) infection (Fig. 6a). In vesicular stomatitis virus (VSV) pseudotype assays, compound 3 modestly decreased 229E spike protein-dependent entry but did not inhibit cell entry by the other envelope proteins tested (Fig. 6b). In contrast, compound 5 was broadly antiviral against VSV-based pseudotypes with VSV, rabies virus, hCoV-229E, or ebola virus envelope proteins. Moreover, inhibition by compound 5 was independent of the pseudotyping platform, because infection of lentiviral particles carrying rabies virus or the cognate VSV glycoprotein was also dose-dependently inhibited (Fig. 6c).

## DISCUSSION

This two-step screen combined with our mechanistic follow-up identified two chemical scaffolds with anti-RSV F protein activity (compounds 3 and 12). These molecules selected for RSV F protein-resistance mutations in the fusion peptide region, an area prone to accumulating resistance mutations (24). However, to our knowledge, these changes have not been previously reported. Therefore, compounds 3 and 12 may serve as starting points for the development of fusion inhibitors with alternative susceptibility to viral resistance mutations. Both compounds revealed comparable RSV strain-dependence, with antiviral activity against all tested RSV A strains and loss of activity against 2 out of 3 clinical RSV B strains. Therefore, these compounds and their derivatization may prove useful in pinpointing the principles which control RSV strain coverage by fusion inhibitors and delivering molecules with particularly broad activity. Such work may also entail identification of fusion inhibitor subclasses, which could be used in combination to increase the barrier to viral resistance and/or as salvage therapy against viruses resistant to a certain fusion inhibitor subtype.

Compound 3 also inhibited hCoV-229E infection, probably also via inhibition of cell entry. This finding suggests that, in principle, cell entry inhibitors against diverse viral targets could be designed. If available, such molecules would be particularly attractive in situations when point-of-care diagnostics are not available and the respiratory disease-causing agent is not precisely diagnosed. Moreover, such broadly active molecules could be a first-line defense against newly emerging viruses. Considering these potential benefits, it will be interesting to better define the mode of action of compound 3 against RSV and hCoV-229E. In the case of RSV, it is well established that typical F protein inhibitors bind to a central cavity formed by the F protein trimers in their pre-fusion conformation, and that compound binding prevents triggering of these complexes for membrane fusion (24). It could be interesting to explore whether other viral fusion proteins also form “pockets” with such properties. If so, this may enable the design of fusion inhibitors with particularly broad activity.

We also identified compound 5, which exhibits broad-spectrum antiviral activity against RSV and hCoV-229E infections and in viral pseudotype entry assays programmed with diverse envelope proteins. This feature, together with the inability of RSV to develop resistance against this compound, make this candidate an interesting molecule for deeper profiling of the mechanism(s) underlying this broad-spectrum antiviral activity.

Likewise, compound 9 did not select for an overt phenotypic viral resistance in HEp-2 cell virus passaging experiments. This molecule was only active against RSV and did not inhibit the other viruses tested. It had comparable activity between the tested RSV strains. Originally, we included this compound because it met inclusion criteria for the second-round infection assay. The evaluation of the synthetic compounds (R)-9s and (S)-9s confirmed this phenotype across both enantiomers. Surprisingly, neither the RSV pseudotype, replicon, time of addition, nor particle-dilution assays revealed an antiviral activity of this molecule. This suggests that it inhibits late stages of the replication cycle such as assembly, release, or cell-to-cell spread. Alternatively, the antiviral activity (for instance, against cell entry) may be relatively weak, such that it is only detectable in the second-round infection assay. This may also preclude detection in the single-round RSV F protein pseudotype infection assays. Another plausible explanation could be that this inhibitor targets the viral G protein, which is not essential for infection of RSV pseudotypes (16); this would explain why the RSV pseudotype assay did not detect antiviral activity. Consistent with this hypothesis, long-term RSV passaging in the presence of compound 9 accumulated two coding mutations within the G protein coding region. However, at least in HEp-2 cells, the virus population with these two mutations did not exhibit resistance in a short-term infection assay. While this result argues against compound 9 targeting RSV G protein and the relevance of these mutations for resistance, the contribution of the G protein in RSV infection of HEp-2 cells may be relatively low. In fact, this limitation may generally complicate identification and validation of G protein inhibitors in cell lines. Johnson et al. (25) reported that G protein-targeting antibodies can be non-neutralizing in immortalized cell lines but neutralizing in primary human airway epithelial (HAE) cell culture systems. Furthermore, they reported that this was due to differential entry factor usage of RSV G protein between these cellular systems and the relevance of chemokine receptor CX3CR1 for infection of HAE cells. It is possible that small molecule inhibitors of RSV G protein are also subject to this difference. In this regard, it is interesting to note that among the tested molecules, compound 9 had the greatest antiviral effect in the differentiated BCi-NS1.1 culture system. This observation could motivate additional studies to confirm the G protein target hypothesis, including the search for tractable culture systems which exhibit RSV G protein-dependent cell entry comparable to that in human primary lung cells. Such models and these types of inhibitors could prove valuable in developing treatment options for RSV-infected patients.

Ultimately, the newly identified scaffolds 3, 5, 9, and 12 share low structural similarity with the licensed drug Ribavirin as well as with compounds in development such as Presatovir or BMS-433771. Ribavirin is a guanosine analogue harboring the typical RNA-derived backbone. None of the identified compounds are nucleic acid-derived structures and therefore likely act via a completely different mode of action. Also, none of the described compounds 3, 5, 9, and 12 possess the benzimidazole/dihydrobenzimidazole of BMS-433771. A minor structural similarity could be assigned between compound 12 and Presatovir with regard to the pyrazolo-pyrimidine (Presatovir) and triazolo-pyrimidine (12) cores. However, these structures show a different decoration with substituents. For example, 12 completely lacks the sulfonamide functionality of Presatovir, rendering both compounds structurally divergent classes. In conclusion, we could identify structurally distinctive small molecules to known and currently investigated drugs, opening up additional potential for the development of novel RSV therapeutics.

## MATERIALS AND METHODS

### Cell lines.

HEK293T/17 (CCL-3216), Calu-3 (HTB-55), Huh-7.5 (26), and BSR T7/5 cells (27) were maintained in Dulbecco’s modified Eagle’s medium supplemented with 10% fetal bovine serum (FBS), 100 U/mL penicillin, 100 μg/mL streptomycin, 1% non-essential amino acids, and 2 mM l-glutamine (Thermo Fisher Scientific, Waltham, MA). Huh-7.5_ACE2 cells were additionally supplemented with 10 μg/mL blasticidin. Vero (CCL-81) and HEp-2 (CCL-23) cells were maintained in advanced MEM media supplemented with 10% FBS, 100 U/mL penicillin, 100 μg/mL streptomycin, 1% non-essential amino acids, and 2 mM l-glutamine. BCi-NS1.1 cells (CVCL_T029) were (23) maintained in Bronchial Epithelial Cell Growth Medium (BEGM) (28). All cells were cultured under 5% CO_2_ at 37°C.

### Clinical RSV isolates.

HEp-2 cells were inoculated with nasopharyngeal washes from RSV-infected children collected at Hanover Medical School (ethic vote MHH no. 6309_10/31/2012). Cells were cultured at 37°C and 5% CO_2_ until syncytium formation was visible. Virus-containing supernatant was collected as described in the supplemental material. Genotyping of isolated virus was performed by partial PCR-amplification using genotype-specific primers, followed by Sanger sequencing and phylogenetic analysis.

### Small molecule compound library.

The small molecule library at the Institute of Virology of Hanover Medical School consists of 58,000 compounds purchased from two commercial suppliers (Enamine, ChemDiv). Compounds were selected to cover a broad chemical space. A subset of 6,048 compounds from this library was screened in this project.

### Resynthesis of compounds 3, 5, 9, and 12.

The resynthesis of compounds 3, 5, 9, and 12 (supplemental information A, Scheme 1) is described in detail in the chemical supplemental material (supplemental information B).

### RSV-B-GFP screening.

We screened a library of 6,048 small molecules with drug-like properties (ChemDiv and Enamine) using a Biomek FXP Automation Workstation. HEp-2 cells were seeded in black, clear-bottomed 384-well plates at a density of 3 × 10^3^ cells per 60 μL/well. At 24 h post-seeding, cells were infected with hRSV B05 eGFP (29) (reporter virus kindly provided by W. Paul Duprex, University of Pittsburgh School of Medicine, Pittsburgh, PA, USA) at an MOI of 1 in the presence of 10 μM compound (1% DMSO). The licensed F protein-targeting antibody Synagis (2 μg/mL) (Palivizumab; AbbVie, Wiesbaden, Germany) was used as a positive control to validate the screen. Furthermore, the screen was statistically validated by Z’-factor analysis (30). The first round of screening was stopped at 48 h postinfection and the supernatant containing virus and compound was transferred onto new cells for the second round of screening, while cells from the first round of screening were processed to measure virus infectivity and cytotoxicity. Virus infectivity was measured via GFP fluorescence. Cytotoxicity was measured with an MTT assay. Infectivity of the second round of infection was measured 48 h postinfection.

### Selection for RSV drug resistance and next generation sequencing.

HEp-2 cells (3 × 10^5^/well) were inoculated with 6 × 10^4^ TCID_50_ of the rHRSV-A-GFP virus (Marie-Anne Rameix-Welti, UMR1173, the Institut National de la Santé et de la Recherche Médicale [INSERM], Université de Versailles St. Quentin, Montigny-le-Bretonneux, France and Jean-François Eléouët, Unité de Virologie et Immunologie Moléculaires, INRA, Université Paris Saclay, Jouy-en-Josas, France) in the presence of compounds or 1% DMSO. Syncytium formation and GFP signal intensity were monitored over time to guide virus passaging and increases in drug dosage over 10 consecutive passages.

RNA Illumina NGS libraries were prepared from each sample after rRNA removal using an NEBNext rRNA Depletion kit v2 followed by NEB Ultra II RNA library preparation (New England Biolabs, Ipswich, MA, USA) according to the manufacturer’s instructions. All libraries were multiplex-sequenced on an Illumina MiSeq instrument (300 cycles, PE protocol) with approximately 4,000,000 reads per sample. Adapter sequences of the reads and bases with a score of less than Q30 were trimmed, and any reads shorter than 36 nt were removed using Trimmomatic v0.36 (31). The paired-end reads were mapped to the host genome (10 mm, GCA_000001635.2) using the Bowtie2 package (32). The resulting non-human sequences were analyzed using CLC Genomics Workbench v10 software (Qiagen Bioinformatics, Hilden, Germany). Unmapped reads were aligned to the sequence of the molecular clone from which the recombinant hRSV subtype A GFP reporter virus had been derived using Novoalign v3.07.00 (http://www.novocraft.com) with the parameters “-r Random -l 20 -g 40 -x 20 -t 100 -k”. We then used the tools samtools (PMID: 19505943) and MarkDuplicate, available in Picard tools (http://broadinstitute.github.io/picard/), to sort the alignments and remove duplicate sequences. Reads which mapped twice or more on the sequence were discarded. The derived alignment was fed into V-Phaser2 (PMID: 24088188) for intra-individual single nucleotide variation (iSNV) identification. For the variant identification, we only considered variants supported by at least five reads on each strand, for which the ratio of the number of reads on the two strands was smaller than 10. Only iSNVs with an allele frequency of >5% were included in the final results.

### Infection of human airway epithelial cells.

Differentiated BCi-NS1.1 cells were cultured in *trans*-wells under air-liquid interface conditions until infection with hRSV-A-GFP (15). Compounds were added with the inoculum. The apical mucus was harvested by incubating the cells with Hanks’ balanced salt solution at 37°C for 20 min. Apical treatment was repeated for 1 h twice a day and basal treatment once daily, until the final mucus harvest at 96 hpi and processing for reverse-transcription quantitative PCR (qRT-PCR) analysis as described previously (18).

### Lentiviral pseudotype preparation and transduction.

Lentiviral pseudotypes were prepared as described previously (16). The viral plasmids used consisted of (i) a packaging plasmid containing the HIV gag-pol genes (pRV8.74) (33); (ii) a transfer plasmid containing a firefly luciferase reporter gene flanked by lentiviral long terminal repeats (pWPI-F-Luc-BLR) (34); and (iii) an envelope plasmid coding the RSV glycoprotein (hRSV-F), Synagis- (K272E) or fusion inhibitor (K394R)-resistant F-mutations (16), VSV-G (pczVSV-G) (35), rabies virus (RABV) glycoprotein (36), or empty vector control (pcDNA3.1). Synagis (Palivizumab; AbbVie, Wiesbaden, Germany) and BMS-433771 (Sigma-Aldrich, St. Louis, MO, USA) were used as controls.

### RSV minigenome replicon assay.

BSR T7/5 cells (27) were seeded in 2 mL growth medium in 6-well plates (5 × 10^5^ cells/well). The next day, 4.75 μg DNA was transfected to the cells in a total volume of 1,250 μL Opti-MEM and 20 μL Lipofectamine 2000 according to the manufacturer’s instructions. The plasmids used encode (i) phosphoprotein P (pCITE_P), (ii) nucleoprotein N (pCITE_N), (iii) polymerase L (pCITE_L), (iv) anti-transcription-termination factor M2-1 (pCITE_M2-1) of a clinical isolate hRSV subtype A genotype ON1 (18), and (v) a subgenomic luciferase replicon of the hRSV strain A2 (37) (ratio of 3.33:3.33:1.7:1:3.33). The respective genes are preceded by a T7 promoter resulting in mRNA expression in the presence of a T7 polymerase. In the 5′-to-3′ direction, the bicistronic subgenomic replicon contains a T7 promoter in front of the 5′-trailer sequence of hRSV, followed by a firefly luciferase-encoding gene separated from an NS1/M chimera by the M/SH intergenic region and completed by the 3′-leader sequence of hRSV and a hepatitis delta ribozyme. Solutions containing all plasmids except the L protein-encoding construct or containing only one plasmid encoding a T7 polymerase-independent firefly luciferase were prepared as negative and positive controls, respectively. Cellular medium was removed, and 750 μL Opti-MEM and the DNA-Lipofectamine mix were added. After incubation at 37°C for 4 h, cells were washed with phosphate-buffered saline (PBS) and re-seeded in 96-well plates (1 × 10^4^ cells/well), followed by addition of the compounds in the indicated concentrations. Ribavirin (Sigma-Aldrich, St. Louis, MO, USA) was used as a control. After 72 h, the cells were lysed, and luciferase activity was determined as a measure for hRSV replication and transcription.

### HCoV-229E RLuc preparation and infection assay.

The renilla luciferase reporter encoding hCoV-229E (38) (kindly provided by Volker Thiel, Institute of Virology and Immunology [IVI], Bern and Mittelhäusern, Switzerland and Department of Infectious Diseases and Pathobiology, Vetsuisse Faculty, University of Bern, Switzerland) was propagated in Huh-7.5 cells at 33°C (5% CO_2_). For compound testing, Huh-7.5 cells were seeded at 2 × 10^4^ cells/well in a 96-well plate in 200 μL of culture medium at 37°C. The next day, hCoV-229E RLuc was diluted in the presence of compounds in the indicated concentrations or DMSO. After incubation at 37°C for 48 h, the cells were lysed by application of 0.5% Triton X-100 in PBS and freezing at −20°C. As a measure of residual infectivity, renilla luciferase activity was measured in 20 μL of the cell lysate (0.2-s measuring time; Berthold Centro plate luminometer version 2.02).

### Data availability.

The sequencing data are stored in the NCBI Sequence Read Archive (SRA) database with the BioProject accession number: PRJNA896514 and SRA accession numbers: SRR22123809, SRR22123810, SRR22123811, SRR22123812, SRR22123813, and SRR22123814.
