# Supplementary material for: Respiratory Syncytial Virus Two-Step Infection Screen Reveals Inhibitors of Early and Late Life Cycle Stages
Source: Antimicrob Agents Chemother. 2022 Nov 8;66(12):e01032-22. doi: 10.1128/aac.01032-22 (PMC9765014; doi:10.1128/aac.01032-22)
Supplement: Supplemental file 1 — Supplemental material. Download aac.01032-22-s0001.pdf, PDF file, 2.3 MB [file aac.01032-22-s0001.pdf]

Supplementary information A

Respiratory syncytial virus two-step infection screen reveals inhibitors of early and late life cycle stages

Svenja M. Sake[a], Christina Kosch[b, c], Sebastian Blockus[a], Sibylle Haid[a], Antonia P. Gunesch[a], Xiaoyu Zhang[a], Martina Friesland[a], Sofie B. Trummer[a], Christina Grethe[a], Anne Kühnel[a], Jessica Rückert[d,e], W. Paul Duprex[f], Jiabin Huang[g], Marie-Anne Rameix-Welti[h], Martin Empting[b, c], Nicole Fischer[g], Anna K. H. Hirsch[b, c, i, j], Thomas F. Schulz[d, e, j], and Thomas Pietschmann[a, e, i, j,#]

[a]Institute for Experimental Virology, Twincore - Centre for Experimental and Clinical Infection Research; Feodor-Lynen-Str. 7, 30625 Hannover, Germany.

[b]Helmholtz Institute for Pharmaceutical Research Saarland (HIPS) - Helmholtz Centre for Infection Research (HZI); Campus Building E8.1, 66123 Saarbrücken, Germany.

[c]Department of Pharmacy, Saarland University; Campus Building E8.1, 66123 Saarbrücken, Germany.

[d]Institute of Virology, Hannover Medical School; Carl Neuberg Str. 1, 30625 Hannover, Germany.

[e]German Centre for Infection Research, Hannover-Braunschweig Site; 30625 Hannover, Germany.

[f]Centre for Vaccine Research, University of Pittsburgh; Pittsburgh, Pennsylvania 15261, USA.

[g]Institute for Medical Microbiology, Virology and Hygiene, University Medical Center Hamburg-Eppendorf; 20246 Hamburg, Germany.

[h]Université Paris-Saclay, Université de Versailles St. Quentin; UMR 1173 (2I), INSERM; Assistance Publique des Hôpitaux de Paris, Hôpital Ambroise Paré, Laboratoire de Microbiologie, DMU15; Versailles, France.

[i]Helmholtz International Lab for Anti-infectives.

[j]Cluster of Excellence RESIST (EXC 2155), Hannover Medical School; 30625 Hannover, Germany.

# Corresponding author: Thomas Pietschmann, Institute for Experimental Virology, Twincore - Centre for Experimental and Clinical Infection Research; Feodor-Lynen-Straße 7, 30625 Hannover, Germany, phone: +49-511-220027130, fax: +49-511-220027131 email: thomas.pietschmann@twincore.de

Email: Thomas.pietschmann@twincore.de

Running title: RSV inhibitors

Contents

1. Supplementary Methods 4

1.1 Preparation of RSV virus stocks 4

1.2 Immunofluorescence staining 4

1.3 Quantification of RSV infection by intracellular RSV P protein staining and FACS analysis 4

1.4 Vesicular stomatitis virus pseudotype preparation and transduction 5

2. Supplementary Schemes 6

3. Supplementary Tables 8

4. List of Tables 11

5. Supplementary References 11

1. Supplementary Methods

1.1 Preparation of RSV virus stocks

Stocks of the recent clinical isolates RSV-A-ON1-H1, RSV-A-GA2-H2, and RSV-B-H3/H4/H5(1, 2) (nasopharyngeal washes kindly provided by the Clinic for Pediatric Pulmonology, Allergology and Neonatology, Hannover Medical School, Hannover, Germany), as well as the RSV B reporter viruses hRSV B05 eGFP(3) and of the firefly luciferase or GFP strain Long reporter viruses rHRSV-A-Luc, rHRSV-A-GFP(4, 5) were prepared in HEp-2 cells (reporter viruses kindly provided by W. Paul Duprex, University of Pittsburgh School of Medicine, Pittsburgh, PA, USA; Marie-Anne Rameix-Welti, UMR1173, Institute National de la Santé et de la Recherche Médicale (INSERM), Université de Versailles St. Quentin, Montigny-le-Bretonneux, France and Jean-François Eléouët, Unité de Virologie et Immunologie Moléculaires, INRA, Université Paris Saclay, Jouy-en-Josas, France). For harvesting, cells were scraped and transferred together with supernatant into 50 mL tubes and vortexed rigorously for 2 min to ensure release of cell bound virus particles. The samples were then centrifuged at 1,000 x g for 5 min to remove cellular debris. The supernatant was mixed with a stabilizing solution (final conc. 100 mM $MgSO_4$, 50 mM HEPES, pH7.5). Aliquots were snap-frozen and stored at -80 °C.

1.2 Immunofluorescence staining

Cells were fixed in 3% paraformaldehyde in PBS for at least 30 min and then washed with PBS before permeabilisation with 0.5% Triton X-100 in PBS for 5 min. Subsequently, cells were incubated with the anti-RSV-P (26D6G5C6) antibody(6) in PBS containing 5% goat serum for 1 h. After washing with PBS, cells were incubated with a fluorescently labelled goat anti-mouse antibody for 1 h, then stained with DAPI (0.5 µg/mL) for 1 min. After washing with H_2O, cover slips were fixed on microscope slides and analyzed on an Olympus IX81 microscope.

1.3 Quantification of RSV infection by intracellular RSV P protein staining and FACS analysis

Cells were detached by trypsinisation and incubated in fixation buffer (0.5% paraformaldehyde, 1% FCS in PBS) for 30 min before permeabilisation in PBS supplemented with 0.1% Saponin at 4 °C for 20 min. Cells were then incubated with anti-RSV-P (26D6G5C6; 2 µg/mL in PBS/1% FCS) antibody (6) for 1 h, washed with PBS, and stained by Alexa-fluor-488 coupled anti-mouse antibodies (10 µg/mL

in PBS/1% FCS) for 30 min. Cells were resuspended in fixation buffer and measured via flow cytometry on the Accuri™ C6 Cytometer or SA3800 Spectral Analyzer and results were evaluated using FlowJo V10.

1.4 Vesicular stomatitis virus pseudotype preparation and transduction

Recombinant VSV-based pseudotypes were produced according to an adapted protocol of Berger Rentsch *et al.* (7). G-protein expression was induced in BHK-G43 cells by addition of 10^{-9} M mifepristone for 6 h. Subsequently, cells were inoculated in a ratio of 1:100 with VSVΔG-G (kindly provided by Gert Zimmer, Institute of Virology and Immunology, Bern, Switzerland) for 24 h. The next day, supernatant was centrifuged for 15 min at 1200 x g at 4 °C and titrated on HEK293T cells. For production of pseudotyped virons, HEK293T were seeded in 10 cm dishes (4.8 x 10^5 cells/well) and transfected the next day. Therefore, 6 µg/µL of envelope plasmids VSV-G, RAB-V, 229E-S (pCAGGS_229E-S, kindly provided by Stefan Pöhlmann, German Primate Centre, Göttingen, Germany), ebola virus glycoprotein ((pVR1012-GP(Z) (Zaire EBOV-GP), kind gift of Dr. Gary Nabel and Anthony Sanchez, NIH, Bethesda, USA), or empty vector was mixed with Lipofectamine 2000 (ratio 1:1) and OptiMEM and a total of 3 mL were added to 6 mL of fresh 3% FCS DMEM w/o antibiotics on the cells. After 4-6 h at 37 °C, media were exchanged for 10 mL 3% FCS DMEM complete. The next day, cells were inoculated with VSVΔG-G (MOI 3) and washed with PBS after 2 h. To 10 mL of fresh media, anti-VSV-G antibody (I1, produced from CRL-2700 mouse hybridoma cells) was added 1:1000 except for the VSV-G transfected wells. After incubation over night at 37 °C, supernatant was collected and centrifuged at 2000 x g for 10 min. Stocks were titrated on target cells as well as on BHK-21 cells. For transduction, Huh-7.5_Ace2 cells were seeded at 1.5 x 10^4 cells/well in 96-well plates and inoculated the next day with the respective pseudotypes and compound **3s**, **5s**, or DMSO in the indicated concentrations. Medium was changed after 4-6 h and cells were lysed after another 16-18 h incubation at 37 °C, followed by FLuc activity measurement.

2. Supplementary Schemes

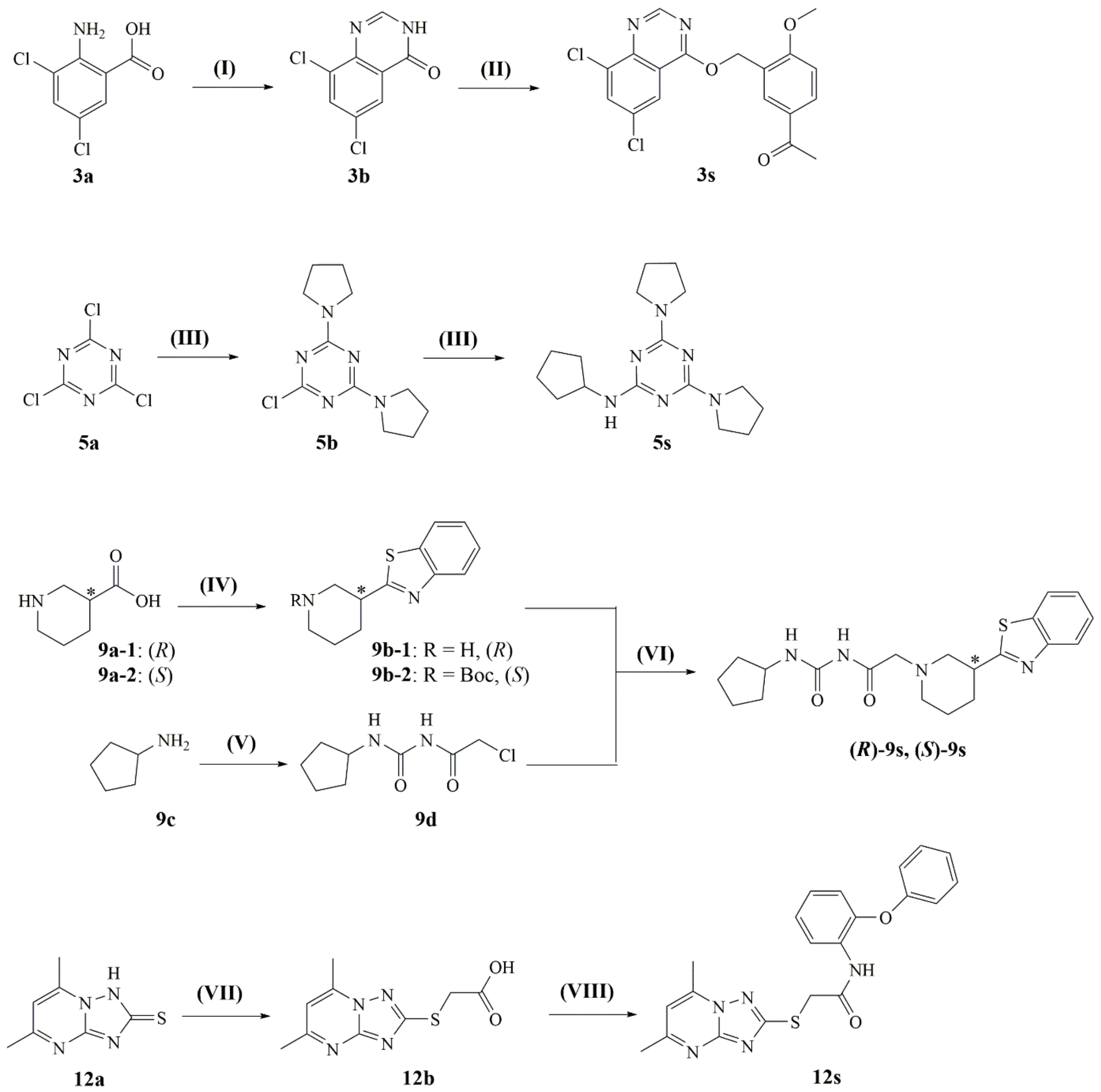

SA-Scheme1: Synthetic pathways for resyntheses and corresponding conditions.

(I) Formamide, acetic acid, 150°C, overnight (o/n), closed vessel, **3b**: 91%. **(II)** 1-(3-(chloromethyl)-4-methoxyphenyl)ethan-1-one, K_2CO_3, *N*,*N*-dimethylformamide (DMF), room temperature (r.t.), o/n, **3s**: 55%. **(III)** Corresponding amine (1.0 - 2.2 equiv.), *N*,*N*-diisopropylethylamine (DIPEA), dichloromethane (DCM), 0°C → r.t., 1 h - 7 days, **5b**: 67%, **5s**: 29%. **(IV)** 2-Aminothiophenol, DIPEA, propanephosphonic acid anhydride (T3P, 50% in DMF), microwave-assisted (mw), 100 °C, 15 min, **9b-1**: 43% (crude), **9b-2**: 28%. **(V)** Chloroacetyl isocyanate, DCM, r.t., o/n, **9d**: 76%. **(VI)** Triethylamine

(NEt$_3$), tetrahydrofuran (THF), r.t., 1 - 2 days, **(*R*)-9s**: 27%, **(*S*)-9s**: 50%. **(VII)** 1) NaOH, H_2O, reflux, 0.5 h, 2) $ClCH_2COOH$, H_2O, reflux, 5 h, **12b**: 61%. **(VIII)** 2-phenoxyaniline, DIPEA, 1-[Bis(dimethylamino)methylene]-1*H*-1,2,3-triazolo[4,5-*b*]pyridinium 3-oxid hexafluorophosphate (HATU), DMF, 120 °C, 2 h, **12s**: 35%.

3. Supplementary Tables

SA-Table 1: IC_{50}, IC_{90} and CC_{50} values of hRSV targeting small molecules [µM].

	Compound	3	5	9	12
Infection round I	IC_{50}	1.1	> 10	> 10	1.0
	IC_{90}	> 100	n.c.	n.c.	63.9
	CC50	> 100	51.6	> 100	> 100
	SI [CC_{50}/IC_{50}]	> 91	n.a.	n.a.	> 100
Infection round II	IC_{50}	0.9	3.4	4.4	0.2
	IC_{90}	23.6	3.7	6.9	6.2
	SI [CC_{50}/IC_{50}]	111	15	>23	> 500
	Dose used in further experiments	25[1]	17[2]	34[2]	64[3]
	Final concentrations in p10	30	12	10	100

n.c., not calculable by the used curve fit algorithm; n.a., not applicable; [1] dose based on IC_{90} round II; [2] dose based on CC_{50} (1/3 of CC_{50}); [3] dose based on IC_{90} round I

SA-Table 2: Purities of commercial and resynthesized compounds according to LC-MS analyses. Purities determined *via* peak integration of peaks in UV/Vis chromatograms (SB-Fig. 34 - 37, SB-Tables 7 - 9, applied system and method described in SB 1.).

Compound	Purity according to LC-MS
**3**	94%
**3s**	> 98%
**5**	96%
**5s**	> 98%
**9***	> 98%
**(*R*)-9s**	> 98%
**(*S*)-9s**	> 98%
**12**	> 98%
**12s**	> 98%

*no stereo information available

SA-Table 3: Compound concentration during virus adaptation [µM].

	Compound			
**Passage**	**3**	**5**	**9**	**12**
**1**	2	4	4	2
**2**	10	8	8	4
**3**	20	8	8	20
**4**	30	8	8	30
**5**	30	4	0	20
**6**	30	4	2	25
**7**	30	6	4	40
**8**	30	8	6	60
**9**	30	10	8	100
**10**	30	12	10	100

SA-Table 4: Amino acid changes in viral proteins of the adapted virus populations (frequency in %).

	Virus population passaged in presence of				
**Protein**	**3**	**5**	**9**	**12**	**DMSO**
**NS1**	**Y88H** (81.17)	-	-	-	-
**NS2**	-	-	-	-	-
**N**	-	**V239I** (8.87)	-	-	-
**P**	**P91H** (17.38)	-	-	-	-
**M**	-	-	-	-	-
**SH**	-	-	-	-	-
**G**	-	-	**L13P** (34.1)<br>**W17R** (32.23)	-	-
**F**	**K68T** (80.62)<br>**L141F** (8.73)<br>**L142I** (80.76)	-	-	**I11F** (6.18)<br>**F137Y** (28.35)<br>**A149T** (58.43)<br>**A552V** (26.03)	-
**M2-1**	-	-	-	-	-
**M2-2**	-	-	-	-	-
**L**	-	-	-	-	-

4. List of Tables

SA-Table 1: IC_{50}, IC_{90} and CC_{50} values of hRSV targeting small molecules [µM]. 8
SA-Table 2: Purities of commercial and resynthesized compounds according to LC-MS analyses. Purities determined *via* peak integration of peaks in UV/Vis chromatograms (SB-Fig. 34 - 37, SB-Tables 7 - 9, applied system and method described in SB 1.). 8
SA-Table 3: Compound concentration during virus adaptation [µM]. 9
SA-Table 4: Amino acid changes in viral proteins of the adapted virus populations (frequency in %). 10

5. Supplementary References

1. Blockus S, Sake SM, Wetzke M, Grethe C, Graalmann T, Pils M, Le Goffic R, Galloux M, Prochnow H, Rox K, Huttel S, Rupcic Z, Wiegmann B, Dijkman R, Rameix-Welti MA, Eleouet JF, Duprex WP, Thiel V, Hansen G, Bronstrup M, Haid S, Pietschmann T. 2020. Labyrinthopeptins as virolytic inhibitors of respiratory syncytial virus cell entry. Antiviral Res 177:104774.
2. Risso-Ballester J, Galloux M, Cao J, Le Goffic R, Hontonnou F, Jobart-Malfait A, Desquesnes A, Sake SM, Haid S, Du M, Zhang X, Zhang H, Wang Z, Rincheval V, Zhang Y, Pietschmann T, Eleouet JF, Rameix-Welti MA, Altmeyer R. 2021. A condensate-hardening drug blocks RSV replication in vivo. Nature 595:596-599.
3. Lemon K, Nguyen DT, Ludlow M, Rennick LJ, Yuksel S, van Amerongen G, McQuaid S, Rima BK, de Swart RL, Duprex WP. 2015. Recombinant subgroup B human respiratory syncytial virus expressing enhanced green fluorescent protein efficiently replicates in primary human cells and is virulent in cotton rats. J Virol 89:2849-56.
4. Rameix-Welti MA, Le Goffic R, Herve PL, Sourimant J, Remot A, Riffault S, Yu Q, Galloux M, Gault E, Eleouet JF. 2014. Visualizing the replication of respiratory syncytial virus in cells and in living mice. Nat Commun 5:5104.
5. Bouillier C, Rincheval V, Sitterlin D, Blouquit-Laye S, Desquesnes A, Eleouet JF, Gault E, Rameix-Welti MA. 2019. Generation, Amplification, and Titration of Recombinant Respiratory Syncytial Viruses. J Vis Exp doi:10.3791/59218.
6. Haid S, Grethe C, Bankwitz D, Grunwald T, Pietschmann T. 2015. Identification of a Human Respiratory Syncytial Virus Cell Entry Inhibitor by Using a Novel Lentiviral Pseudotype System. J Virol 90:3065-73.
7. Berger Rentsch M, Zimmer G. 2011. A Vesicular Stomatitis Virus Replicon-Based Bioassay for the Rapid and Sensitive Determination of Multi-Species Type I Interferon. PLOS ONE 6:e25858.

Supplementary information B

Respiratory syncytial virus two-step infection screen reveals inhibitors of early and late life cycle stages

Svenja M. Sake[a] , Christina Kosch[b, c], Sebastian Blockus[a] , Sibylle Haid[a], Antonia P. Gunesch[a], Xiaoyu Zhang[a], Martina Friesland[a], Sofie B. Trummer[a] , Christina Grethe[a], Anne Kühnel[a], Jessica Rückert[d,e], W. Paul Duprex[f], Jiabin Huang[g], Marie-Anne Rameix-Welti[h], Martin Empting[b, c], Nicole Fischer[g], Anna K. H. Hirsch[b, c, i, j], Thomas F. Schulz[d, e, j], and Thomas Pietschmann[a, e, i, j,#]

[a]Institute for Experimental Virology, Twincore - Centre for Experimental and Clinical Infection Research; Feodor-Lynen-Str. 7, 30625 Hannover, Germany.

[b]Helmholtz Institute for Pharmaceutical Research Saarland (HIPS) - Helmholtz Centre for Infection Research (HZI); Campus Building E8.1, 66123 Saarbrücken, Germany.

[c]Department of Pharmacy, Saarland University; Campus Building E8.1, 66123 Saarbrücken, Germany.

[d]Institute of Virology, Hannover Medical School; Carl Neuberg Str. 1, 30625 Hannover, Germany.

[e]German Centre for Infection Research, Hannover-Braunschweig Site; 30625 Hannover, Germany.

[f]Centre for Vaccine Research, University of Pittsburgh; Pittsburgh, Pennsylvania 15261, USA.

[g]Institute for Medical Microbiology, Virology and Hygiene, University Medical Center Hamburg-Eppendorf; 20246 Hamburg, Germany.

[h]Université Paris-Saclay, Université de Versailles St. Quentin; UMR 1173 (2I), INSERM; Assistance Publique des Hôpitaux de Paris, Hôpital Ambroise Paré, Laboratoire de Microbiologie, DMU15; Versailles, France.

[i]Helmholtz International Lab for Anti-infectives.

[j]Cluster of Excellence RESIST (EXC 2155), Hannover Medical School; 30625 Hannover, Germany.

# Corresponding author: Thomas Pietschmann, Institute for Experimental Virology, Twincore - Centre for Experimental and Clinical Infection Research; Feodor-Lynen-Straße 7, 30625 Hannover, Germany, phone: +49-511-220027130, fax: +49-511-220027131 email: thomas.pietschmann@twincore.de

Email: Thomas.pietschmann@twincore.de

Running title: RSV inhibitors

Contents

1. General information 4

2. General procedures 6

2.1 General procedure A (GP A) 6

2.2 General procedure B (GP B) 6

2.3 General Procedure C (GP C) 6

3. Experimental details 7

3.1 6,8-Dichloroquinazolin-4(3*H*)-one (3b) 7

3.2 1-(3-(((6,8-Dichloroquinazolin-4-yl)oxy)methyl)-4-methoxyphenyl)ethan-1-one (3s) 7

3.3 2-Chloro-4,6-di(pyrrolidin-1-yl)-1,3,5-triazine (5b) 8

3.4 *N*-Cyclopentyl-4,6-di(pyrrolidin-1-yl)-1,3,5-triazin-2-amine (5s) 8

3.5 (*R*)-2-(piperidin-3-yl)benzo[*d*]thiazole (9b-1) 8

3.6 (*S*)-2-(1-Boc-piperidin-3-yl)benzo[*d*]thiazole (9b-2) 8

3.7 2-Chloro-*N*-(cyclopentylcarbamoyl)acetamide (9d) 9

3.8 (*R*)-2-(3-(benzo[*d*]thiazol-2-yl)piperidin-1-yl)-*N*-(cyclopentylcarbamoyl)acetamide ((*R*)-9s) 10

3.9 (*S*)-2-(3-(benzo[*d*]thiazol-2-yl)piperidin-1-yl)-*N*-(cyclopentylcarbamoyl)acetamide ((*S*)-9s) 10

3.10 2-((5,7-Dimethyl-[1,2,4]triazolo[1,5-a]pyrimidin-2-yl)thio)acetic acid (12b) 11

3.11 2-((5,7-dimethyl-[1,2,4]triazolo[1,5-a]pyrimidin-2-yl)thio)-*N*-(2-phenoxyphenyl)acetamide (12s)... 11

4. Appendix 13

4.1 NMR spectra 13

4.2 LC-MS data 25

4.3 HRMS data 36

5. List of Figures 39

6. List of Tables 40

7. Supplementary References 40

1. General information

This document describes the general procedures, experimental details as well as characterizations of chemically synthesized compounds **3s**, **5s**, ***(R)*-9s**, ***(S)*-9s**, and **12s**. Furthermore, details regarding the performed purity analyses of screening compounds are included.

All chemicals were used as obtained from commercial suppliers without further purification.

^{1}H and ^{13}C nuclear magnetic resonance (NMR) spectra were recorded on a Bruker Fourier 500 [500 MHz (^{1}H), 126 MHz (^{13}C)] spectrometer. Chemical shifts were given in parts per million (ppm) and referenced against the residual dimethyl sulfoxide-d_6 (DMSO-d_6), Chloroform-d ($CDCl_3$), or acetone-d_6 peak, while coupling constants (J) were indicated in hertz (Hz). Multiplicities were described with singlet (s), broad singlet (br s), doublet (d), doublet of a doublet (dd), doublet of a doublet of a doublet (ddd), triplet (t), doublet of a triplet (dt), quartet (q), doublet of a quartet (dq), sextet (sxt), septet (sept), and multiplet (m).

For reaction controls, thin layer chromatography (TLC) and/or liquid chromatography-mass spectrometry (LC-MS) were used. Low-resolution mass analytics and purity control of final compounds were performed by applying a SpectraSystems-MSQ LC-MS system (Thermo Fisher Scientific) consisting of pump, Hypersil Gold column (100 mm x 2.1 mm, particle size 3 µm), autosampler, VWD detector and an ESI quadrupole mass spectrometer. For LC-MS measurements, the following method was used: positive/negative mode, acetonitrile (ACN + 0.1% formic acid (FA)/water (H_2O) + 0.1% FA, 5 – 100% ACN + 0.1% FA over 5.8 min, 0.7 mL/min, wavelength (λ) = 254 nm.

Microwave-assisted (mw) syntheses were carried out in a Discover microwave synthesis system from CEM. For column chromatography, either the automated flash column chromatography (AFC) system CombiFlash Rf 150 (Teledyne Isco) equipped with RediSepRf silica columns was used or manual flash column chromatography (MFC) with Silica 60 M, 0.04 - 0.063 mm or 0.063 - 0.2 mm, (Macherey-Nagel) was performed.

Final products were dried under high vacuum. In case the final compound was purified with semi-preparative high performance liquid chromatography (semi-prep HPLC), the corresponding isolated fraction was lyophilized using a Christ Alpha 2-4 LD plus freeze-dryer connected to Chemistry Hybrid Pump RC6 (Vacuubrand). For semi-prep HPLC, an Ultimate 3000 ultra-high performance liquid

chromatography (UHPLC) system (Thermo Fisher Scientific) equipped with Dionex RS Pump, Diode Array Detector, Automated Fraction Collector, Nucleodur C18 Gravity column (250 mm x 10 mm (column A) or 16 mm (column B), particle size 5 µm) was used.

High resolution mass spectrometry (HRMS) measurements were conducted with a Q Exactive Focus (Thermo Fischer Scientific) connected to Dionex Ultimate 3000 RS Pump and Autosampler as well as UHPLC system Column compartment with Nucleodur C18 Pyramid column (150 mm x 2 mm, particle size 3 µm) and Diode Array Detector.

2. General procedures

2.1 General procedure A (GP A)

The general procedure A was performed as described in the protocol of Cheng *et al.*[1]. The corresponding amine (1.0 – 2.2 equivalents (equiv.)) and *N,N*-diisopropylethylamine (DIPEA, 2.2 - 3.0 equiv.) were added dropwise to a solution of the related starting material in dry dichloromethane (DCM, 0.54 M) at 0 °C. The mixture was stirred at the same temperature or reaching room temperature. After a full conversion detected *via* LC-MS, the reaction mixture was washed with hydrochloric acid (HCl, 2 M, aqueous (aq.)). From this aqueous layer, the crude was extracted twice with DCM. After combining all organic layers and drying them over $MgSO_4$, the solvent was removed *in vacuo*. The crude was purified *via* AFC to isolate the related product.

2.2 General procedure B (GP B)

Piperidine-3-carboxylic acid **9a-1** or **9a-2**, DIPEA (1.5 equiv.), propanephosphonic acid anhydride (T3P, 50% in *N,N*-dimethylformamide (DMF), 1.0 equiv.), and 2-aminothiophenol (1.2 equiv.) were heated mw to 100 °C for 15 min[2]. For **9b-1**, the reaction mixture was concentrated *in vacuo*, and water (5 mL) was added to the residue. For **9b-1**, the pH was adjusted to 8 with Na_2CO_3 solution (aq., saturated (sat.)). From this aqueous mixture the crude was extracted with EtOAc (2 x 10 mL), and the combined organic layers were dried over $MgSO_4$ and *in vacuo*. For **9b-2**, the same procedure was applied without changing the pH of the aqueous layer, washing with Brine (10 mL) after extraction and using Na_2SO_4 for drying the organic phase[2]. The resulting crude product was either used without further purification (**9b-1**), or purified via MFC (**9b-2**).

2.3 General Procedure C (GP C)

In case of a Boc-protected starting material, the compound was first dissolved in DCM (0.1 M), cooled to 0 °C and treated with HCl (4 N in 1,4-dioxane, 10.0 equiv.). The mixture was stirred overnight reaching room temperature. After removing the solvent, the resulting HCl salt was directly used for the substitution reaction. **9b-1** or deprotected **9b-2**, respectively, triethylamine (NEt_3, 1.1 equiv. for amines or 2.2 equiv. for HCl salts), and **9d** (1.0 equiv.) were stirred in tetrahydrofuran (THF, 0.45 M) at room temperature for 1 – 2 days. Then, the reaction mixture was poured into ice and the formed beige solid

was extracted with DCM. After drying the organic layer over $MgSO_4$, the solvent was removed *in vacuo*. The obtained crude product was purified *via* AFC and/or semi-prep HPLC[3].

3. Experimental details

3.1 6,8-Dichloroquinazolin-4(3*H*)-one (3b)

Adapting literature known conditions[4, 5] slightly, 2-aminobenzoic acid **3a** (50.0 mg, 0.24 mmol), acetic acid (0.2 mL, 1.2 M) and formamide (2.0 mL, 0.12 M) were stirred at 150 °C in a closed vessel overnight. The reaction mixture was cooled to room temperature and poured into ice water. The formed, white to yellow solid was filtered, washed with water, transferred with acetone and dried *in vacuo* to obtain **3b** (47.7 mg, 0.22 mmol, 91%) as a colorless solid. **^{1}H NMR** (DMSO-d_6, 500 MHz) δ = 12.70 (br s, 1H), 8.25 (s, 1H), 8.15 (d, 1H, J=2.4 Hz), 8.03 (d, 1H, J=2.4 Hz).* **^{13}C NMR** (DMSO-d_6, 126 MHz) δ = 159.4, 146.9, 144.3, 134.1, 132.4, 130.7, 125.1, 124.2. **LC-MS** ($[M+H]^+$) = 214.96 Retention time regarding UV-chromatogram ($t_{r,UV}$) = 3.28 min, *purity* 98%.

3.2 1-(3-(((6,8-Dichloroquinazolin-4-yl)oxy)methyl)-4-methoxyphenyl)ethan-1-one (3s)

3b (50.0 mg, 0.77 mmol) was dissolved in DMF. To this solution, K_2CO_3 (32.2 mg, 0.23mmol) and 1-(3-(chloromethyl)-4-methoxyphenyl)ethan-1-one (69.3 mg, 0.35 mmol) were added. The mixture was stirred at room temperature overnight. After concentrating the mixture *in vacuo,* water was added to the residue. The crude was extracted from the aqueous mixture using DCM. Then, the combined organic layers were dried over $MgSO_4$ and the solvent was removed *in vacuo*[6]. After additional washing with ethyl acetate (EtOAc) the pure product **3s** (49.0 mg, 0.13 mmol, 55%) was obtained as a colorless solid. **^{1}H NMR** (DMSO-d_6, 500 MHz) δ = 8.64 (s, 1H), 8.16 (d, 1H, *J*=2.4 Hz), 8.04 (d, 1H, *J*=2.4 Hz), 7.97 (dd, 1H, *J*=2.2, 8.6 Hz), 7.84 (d, 1H, *J*=2.2 Hz), 7.14 (d, 1H, *J*=8.6 Hz), 5.7-5.8 (m, 1H), 5.15 (s, 2H), 3.92 (s, 3H), 2.50 (s, 3H) †. **^{13}C NMR** (DMSO-d_6, 126 MHz) δ = 196.3, 161.1, 158.8, 149.8, 143.3, 134.1, 132.3, 131.1, 131.0, 130.0, 129.4, 124.4, 124.0, 123.4, 110.8, 56.1, 46.2, 26.4. **LC-MS** *m/z* ($[M+H]^+$, $[M-C_{10}H_{11}O_2]^+$) = 376.95, 164.08, $t_{r,UV}$ = 4.49 min, *purity* > 98%. **HRMS** *calculated: m/z* ($[M+H]^+$) = 377.0454, *found*: *m/z* ($[M+H]^+$) = 377.0448.

* ^{1}H NMR published by Orfi *et al.*[4], but they reported another tautomer in DMSO-d_6; Li *et al.*[6] published ^{1}H NMR in Chloroform-*d*.

† Overlapping with solvent peak, by recording Heteronuclear Single Quantum Coherence spectrum (HSQC - 2D spectrum) this could be confirmed (cf. SB-Fig. 5)

3.3 2-Chloro-4,6-di(pyrrolidin-1-yl)-1,3,5-triazine (5b)

According to **GP A**, **5b** was synthesized using cyanuric chloride **5a** (100.0 mg, 0.54 mmol), DIPEA (0.21 mL, 1.19 mmol) and pyrrolidine (0.10 mL, 1.193 mmol) in DCM (1.0 mL). The reaction was fully converted after 1 h. After purification *via* AFC (petroleum ether (PE/EtOAc, 0 – 100% EtOAc over 23 min, 18 mL/min, 4 g silica, product at 22% EtOAc), **5b** was obtained (92.2 mg, 0.36 mmol, 67%) as a colorless solid. **^{1}H NMR** ($CDCl_3$, 500 MHz) δ = 3.5-3.6 (m, 8H), 1.9-2.0 (m, 8H). **^{13}C NMR** ($CDCl_3$, 126 MHz) δ = 168.3, 162.6, 46.4, 46.2, 25.3, 25.0. **LC-MS** *m/z* ([M+H]$^{+}$) = 253.97, $t_{r,UV}$ = 4.62 min, *purity* > 98%.

3.4 *N*-Cyclopentyl-4,6-di(pyrrolidin-1-yl)-1,3,5-triazin-2-amine (5s)

5s was synthesized according to **GP A** mixing **5b** (50.0 mg, 0.20 mmol), DIPEA (0.12 mL, 0.59 mmol) and cyclopentylamine (0.06 mL, 0.59 mmol) in DCM (0.5 mL). The solution was stirred for 7 days at room temperature to afford **5s** (17.2 mg, 0.057 mmol, 29%) as a colorless solid after purification *via* AFC (cyclohexane/EtOAc, 15 - 55% EtOAc over 17 min, 18 mL/min, product at 15% EtOAc). **^{1}H NMR** ($CDCl_3$, 500 MHz) δ = 4.69 (br d, 1H, *J*=6.9 Hz), 4.29 (sxt, 1H, *J*=6.9 Hz), 3.52 (br s, 8H), 2.0-2.1 (m, 2H), 1.89 (m, 8H), 1.6-1.7 (m, 2H), 1.5-1.6 (m, 2H), 1.4-1.5 (m, 2H). **^{13}C NMR** ($CDCl_3$, 126 MHz) δ = 52.3, 45.8, 33.3, 25.3, 23.8. **LC-MS** *m/z* ([M+H]$^{+}$) = 303.15, $t_{r,UV}$ = 4.04 min, *purity* > 98%. **HRMS** *calculated*: *m/z* ([M+H]$^{+}$) = 303.2292, *found*: *m/z* ([M+H]$^{+}$) = 303.2286.

3.5 (*R*)-2-(piperidin-3-yl)benzo[*d*]thiazole (9b-1)

Following **GP B**, **9b-1** was synthesized. For this, **9a-1** (100.0 mg, 0.77 mmol), DIPEA (0.2 mL, 1.16 mmol), T3P (50% in DMF, 500 mg, 0.79 mmol), and 2-aminothiophenol (0.1 mL, 0.96 mmol) were used. The resulting crude **9b-1** (72.1 mg, 0.33 mmol, 43%) was used without further purification. **^{1}H NMR** ($CDCl_3$, 500 MHz) δ = 7.99 (d, 1H, *J*=8.1 Hz), 7.87 (d, 1H, *J*=8.1 Hz), 7.47 (t, 1H, *J*=7.6 Hz), 7.37 (t, 1H, *J*=7.6 Hz), 3.47 (br d, 1H, *J*=12.1 Hz), 3.2-3.4 (m, 1H), 3.11 (br d, 1H, *J*=12.4 Hz), 3.04 (t, 1H, *J*=11.5 Hz), 2.77 (t, 1H, *J*=11.5 Hz), 2.2-2.4 (m, 1H), 1.9-1.9 (m, 1H), 1.8-1.9 (m, 1H), 1.6-1.7 (m, 1H). **^{13}C NMR** ($CDCl_3$, 126 MHz) δ = 174.0, 153.0, 134.5, 125.9, 124.8, 122.7, 121.5, 51.8, 46.3, 42.4, 31.4, 29.7, 25.6. **LC-MS** *m/z* ([M+H]$^{+}$) = 219.00, t_r (UV) = 0.93 min, *purity* 62%.

3.6 (*S*)-2-(1-Boc-piperidin-3-yl)benzo[*d*]thiazole (9b-2)

First, (*S*)-1-Boc-piperidine-3-carboxylic acid was prepared from starting material **9a-2** (100.0 mg, 0.77 mmol) by stirring its solution in THF/H_2O (v/v 5/1, 2.6 mL) with Na_2CO_3 (82.0 mg, 0.77 mmol)

and di-*tert*-butyl bicarbonate (Boc_2O, 168.9 mg, 0.77 mmol) at 0 °C overnight reaching room temperature. The resulting mixture was diluted with water, adjusted to pH 7 using NH_4Cl (aq., sat.), and extracted with EtOAc (3 x 10 mL). Furthermore, the organic layers were combined, dried over Na_2SO_4 and *in vacuo* to isolate the Boc-protected compound (139.0 mg, 0.61 mmol, 78%). A part of it (86.4 mg, 0.38 mmol) was directly used for the next step without further purification following **GP B** under usage of DIPEA (0.10 mL, 0.57mmol), T3P (50% in DMF, 0.11 mL, 0.38 mmol) and 2-aminothiophenol (0.04 mL, 0.38 mmol). The resulting crude was purified by MFC (PE/EtOAc, 3 – 6% EtOAc, ~5 g silica Mesh 60 (0.04 - 0.063 mm)) obtaining **9b-2** (34 mg, 0.107 mmol, 28%) as a pink sticky oil. **R_f** (PE/EtOAc, 93/7) = 0.08. **^{1}H NMR** ($CDCl_3$, 500 MHz) δ = 8.00 (d, 1H, *J*=8.1 Hz), 7.87 (d, 1H, *J*=8.1 Hz), 7.47 (t, 1H, *J*=7.6 Hz), 7.37 (t, 1H, *J*=7.6 Hz), 4.40 (br s, 1H), 4.07 (br d, 1H, *J*=6.1 Hz), 3.2-3.3 (m, 1H), 3.19 (br s, 1H), 2.9-3.0 (m, 1H), 2.3-2.3 (m, 1H), 1.8-1.9 (m, 2H), 1.6-1.7 (m, 1H), 1.5-1.5 (m, 9H). **^{13}C NMR** ($CDCl_3$, 126 MHz) δ 172.7, 154.7, 153.0, 134.5, 126.0, 124.9, 122.8, 121.6, 79.8, 48.8[‡], 44.0[§], 41.4, 31.3, 28.4, 24.6. **LC-MS** *m/z* ($[M+H]^+$, $[M\text{-}Boc]^+$) = 319.23, 219.12, $t_{r,UV}$ = 5.06 min, *purity* > 98%.

3.7 2-Chloro-*N*-(cyclopentylcarbamoyl)acetamide (9d)

Chloroacetyl isocyanate (168.4 mg, 1.41 mmol) was added dropwise to a solution of cyclopentyl amine **9c** (100.0 mg, 1.17 mmol) in dry DCM (2.0 mL) and stirred at room temperature overnight. After removing the solvent *in vacuo* and washing with cold ether, the residue was recrystallized from ethanol (EtOH)/H_2O (v/v 1/1, 10 mL). Additionally, the resulting solid was washed with cold water, to isolate **9d** (182.3 mg, 0.891 mmol, 76%) as a colorless solid[7]. **^{1}H NMR** (acetone-d_6, 500 MHz) δ = 9.54 (br s, 1H), 8.12 (br s, 1H), 4.30 (s, 2H), 4.09 (sxt, 1H, *J*=6.4 Hz), 1.94 (qd, 2H, *J*=6.4, 12.4 Hz), 1.7-1.7 (m, 2H), 1.6-1.7 (m, 2H), 1.4-1.5 (m, 2H). **^{13}C NMR** (acetone-d_6, 126 MHz) δ = 168.5, 152.3, 152.3, 51.8, 43.2, 33.0, 23.6. **HRMS** *calculated*: *m/z* ($[M+H]^+$) = 205.0738, *found*: *m/z* ($[M+H]^+$) = 205.0739.

‡ Weak signal

§ Weak signal

3.8 (*R*)-2-(3-(benzo[*d*]thiazol-2-yl)piperidin-1-yl)-*N*-(cyclopentylcarbamoyl)acetamide ((*R*)-9s)

According to **GP C***,* **9b-1** (68.8 mg, 0.315 mmol), THF (0.70 mL), NEt_3 (0.05 mL, 0.35 mmol,) and **9d** (65.5 mg, 0.32 mmol) were stirred at room temperature for 1 day. The obtained crude was purified by AFC (cyclohexane/EtOAc, 0 – 45% EtOAc over 14 min, 18 mL/min, 4 g silica, product at 10% EtOAc) and additionally by semi-prep HPLC (ACN + 0.05% FA/ H_2O + 0.05% FA, 5 – 100% ACN + 0.05% FA over 55 min, 5 mL/min, column A, product at 38% ACN + 0.05% FA) giving **9-(*R*)** (32.7 mg, 0.085 mmol, 27%) as a sticky colorless oil. Ratio (rotamer A/rotamer B) = 4/5: Rotamer A: **^{1}H NMR** ($CDCl_3$, 500 MHz) δ = 9.14 (br s, 1H), 8.19 (br s, 1H), 8.00 (br d, 1H, *J*=8.1 Hz), 7.79 (d, 1H, *J*=8.1 Hz), 7.41 (t, 1H, *J*=7.6 Hz), 7.30 (t, 1H, *J*=7.6 Hz), 4.1-4.1 (m, 1H), 4.03 (s, 2H), 3.40 (br s, 1H), 3.10 (br s, 3H), 2.73 (br s, 2H), 2.35 (br s, 1H), 2.15 (br s, 1H), 1.9-2.0 (m, 2H), 1.7-1.8 (m, 1H), 1.6-1.7 (m, 2H), 1.5-1.6 (m, 2H), 1.4-1.5 (m, 2H). Rotamer B: **^{1}H NMR** ($CDCl_3$, 500 MHz) δ = 8.49 (br s, 1H), 8.03 (2 br s, 1H), 8.00 (br d, 1H, *J*=8.1 Hz), 7.79 (d, 1H, *J*=8.1 Hz), 7.40 (t, 1H, *J*=7.6 Hz), 7.30 (t, 1H, *J*=7.6 Hz), 4.1-4.1 (m, 1H), 4.03 (s, 2H), 3.40 (br s, 1H), 3.10 (br s, 3H), 2.73 (br s, 2H), 2.35 (br s, 1H), 2.15 (br s, 1H), 1.9-2.0 (m, 2H), 1.7-1.8 (m, 1H), 1.6-1.7 (m, 2H), 1.5-1.6 (m, 2H), 1.4-1.5 (m, 2H). **^{13}C NMR** ($CDCl_3$, 126 MHz) δ = 166.3, 152.0, 151.2, 150.6, 133.4, 125.1, 124.0, 121.9, 120.5, 53.1, 50.8, 50.6, 41.4, 32.1, 32.0, 29.4, 22.6, 22.5. **LC-MS** *m/z* ($[M+H]^+$, $[M-C_5H_{10}N+H]^+$) = 387.01, 301.99 , $t_{r,UV}$ = 3.33 min, *purity* > 98%. **HRMS** *calculated*: *m/z* ($[M+H]^+$) =387.1849,; *found*: *m/z* ($[M+H]^+$) = 387.1842.

3.9 (*S*)-2-(3-(benzo[*d*]thiazol-2-yl)piperidin-1-yl)-*N*-(cyclopentylcarbamoyl)acetamide ((*S*)-9s)

Following **GP C**, **(*S*)-9** was prepared using **9b-2** (27.4 mg, 0.079 mmol), HCl (4 N in 1,4-dioxane, 0.20 mL) in DCM (0.79 mL) for the deprotection step, and NEt_3 (0.02 mL, 0.17 mmol) as well as **9d** (16.2 mg, 0.079 mmol) in THF (0.16 mL) for the substitution reaction. The reaction mixture was stirred at room temperature for 2 days. The obtained crude was purified *via* semi-prep HPLC (ACN + 0.05% FA/H_2O + 0.05% FA, 5 - 100% ACN + 0.05% FA over 30 min, 10 mL/min, column B, product at 37% ACN + 0.05% FA) giving **9-(*S*)** (15.08 mg, 0.085 mmol, 50%) as a colorless solid. **^{1}H NMR** (DMSO-d_6, 500 MHz) δ = 9.90 (s, 1H), 8.27 (br d, 1H, *J*=6.9 Hz), 8.05 (d, 1H, *J*=8.1 Hz), 8.01 (br d, 1H, *J*=8.1 Hz), 7.48 (t, 1H, *J*=7.6 Hz), 7.40 (t, 1H, *J*=7.6 Hz), 4.00 (sxt, 1H, *J*=6.7 Hz), 3.40

(br s, 1H), 3.38 (br s, 1H), 3.27 (d, 1H, *J*=16.3 Hz), 3.19 (d, 1H, *J*=16.3 Hz), 3.12 (br d, 1H, *J*=9.3 Hz), 2.73 (br d, 1H, *J*=10.5 Hz), 2.65 (t, 1H, *J*=9.6 Hz), 2.3-2.4 (m, 1H), 2.0-2.1 (m, 1H), 1.86 (qd, 2H, *J*=6.4, 12.4 Hz), 1.6-1.7 (m, 5H), 1.5-1.6 (m, 2H), 1.41 (qd, 2H, *J*=6.4, 12.4 Hz). **^{13}C NMR** (DMSO-d_6, 126 MHz) δ = 196.4, 173.7, 172.3, 163.3, 152.5, 152.2, 134.2, 126.0, 124.9, 122.5, 122.0, 60.8, 57.4, 52.8, 50.8, 40.6, 32.6, 30.1, 24.1, 23.1. **LC-MS** *m/z* ($[M+H]^+$, $[M- C_5H_{10}N+H]^+$) = 367.31, 302.17 , $t_{r,UV}$ = 3.38 min, *purity* > 98%. **HRMS** *calculated*: *m/z* ($[M+H]^+$) = 387.1849; *found*: *m/z* ($[M+H]^+$) = 387.1845.

3.10 2-((5,7-Dimethyl-[1,2,4]triazolo[1,5-a]pyrimidin-2-yl)thio)acetic acid (12b)

Similar to the literature known procedure[8], **12b** was synthesized. For this synthesis, **12a** (100.0 mg, 0.56 mmol), sodium hydroxide (44.4 mg, 1.11 mmol), and 2-chloroacetic acid (52.4 mg, 0.56 mmol) were used. After acidifying the reaction mixture to pH 2-3 using HCl (5.0 mL, 2 M), no product precipitated. Therefore, the product was extracted with DCM (3 x 5mL) instead of isolation by filtration and recrystallization from DMF. The combined organic layers were dried over $MgSO_4$ and the solvent was removed *in vacuo* to obtain **12b** (81.2 mg, 0.34 mmol, 61%) as a white solid. This product was used without further purification. **^{1}H NMR** (DMSO-d_6, 500 MHz) δ = 12.87 (br s, 1H), 7.12 (s, 1H), 4.08 (s, 2H), 2.66 (s, 3H), 2.55 (s, 3H). **^{13}C NMR** (DMSO-d_6, 126 MHz) δ = 169.8, 165.1, 164.2, 155.0, 146.3, 110.4, 54.9, 33.1, 24.4, 16.5. **LC-MS** *m/z* ($[M+H]^+$) = 238.97, $t_{r,UV}$ = 2.24 min, *purity* 91%.

3.11 2-((5,7-dimethyl-[1,2,4]triazolo[1,5-a]pyrimidin-2-yl)thio)-*N*-(2-phenoxyphenyl)acetamide (12s)

To a solution of carboxylic acid **12b** (50.0 mg, 0.21 mmol) in dry DMF (0.35 M), DIPEA (0.04 mL, 0.23 mmol) was added. This solution was stirred at room temperature for 10 min. After addition of 1-[Bis(dimethylamino)methylene]-1*H*-1,2,3-triazolo[4,5-*b*]pyridinium 3-oxid hexafluorophosphate (HATU, 95.4 mg, 0.25 mmol) to the mixture and further stirring for 10 min at room temperature, 2-phenoxyaniline (38.7 mg, 0.21 mmol) was added. The solution was stirred at 120 °C for 2 h, cooled to room temperature, and concentrated *in vacuo*. After adding NH_4Cl (sat., aq.) to the residue the product was extracted with DCM for three times. The organic layers were combined and dried over $MgSO_4$. Then, the solvent was removed *in vacuo*. To isolate the product, the crude was purified by AFC (cyclohexane/EtOAc, gradient: 0 – 100% EtOAc over 25 min, 4 g silica, product at 75% EtOAc) obtaining **12s** (29.8 mg, 0.074 mmol, 35%) as a colorless solid. **^{1}H NMR** ($CDCl_3$, 500 MHz) δ = 9.68 (br s, 1H), 8.39 (dd, 1H, *J*=1.5, 8.5 Hz), 7.0-7.1 (m, 3H), 6.94 (t, 2H, *J*=7.3 Hz), 6.67 (td, 3H, *J*=1.5, 8.5 Hz),

6.58 (br s, 1H), 4.07 (s, 2H), 2.61 (s, 2H), 2.6-2.7 (m, 1H), 2.52 (s, 2H), 2.4-2.6 (m, 1H). **^{13}C NMR** ($CDCl_3$, 126 MHz) δ = 167.6, 166.4, 164.4, 156.2, 155.3, 146.6, 145.7, 129.9, 129.3, 124.2, 123.8, 123.4, 121.4, 118.8, 117.6, 110.2, 35.2, 24.8, 16.9. **LC-MS** *m/z* ($[M+H]^+$) = 405.99, $t_{r,UV}$ = 4.16 min, *purity* > 98%. **HRMS** *calculated*: *m/z* ($[M+H]^+$) =406.1332; *found*: *m/z* ($[M+H]^+$) = 406.1327.

4. Appendix

4.1 NMR spectra

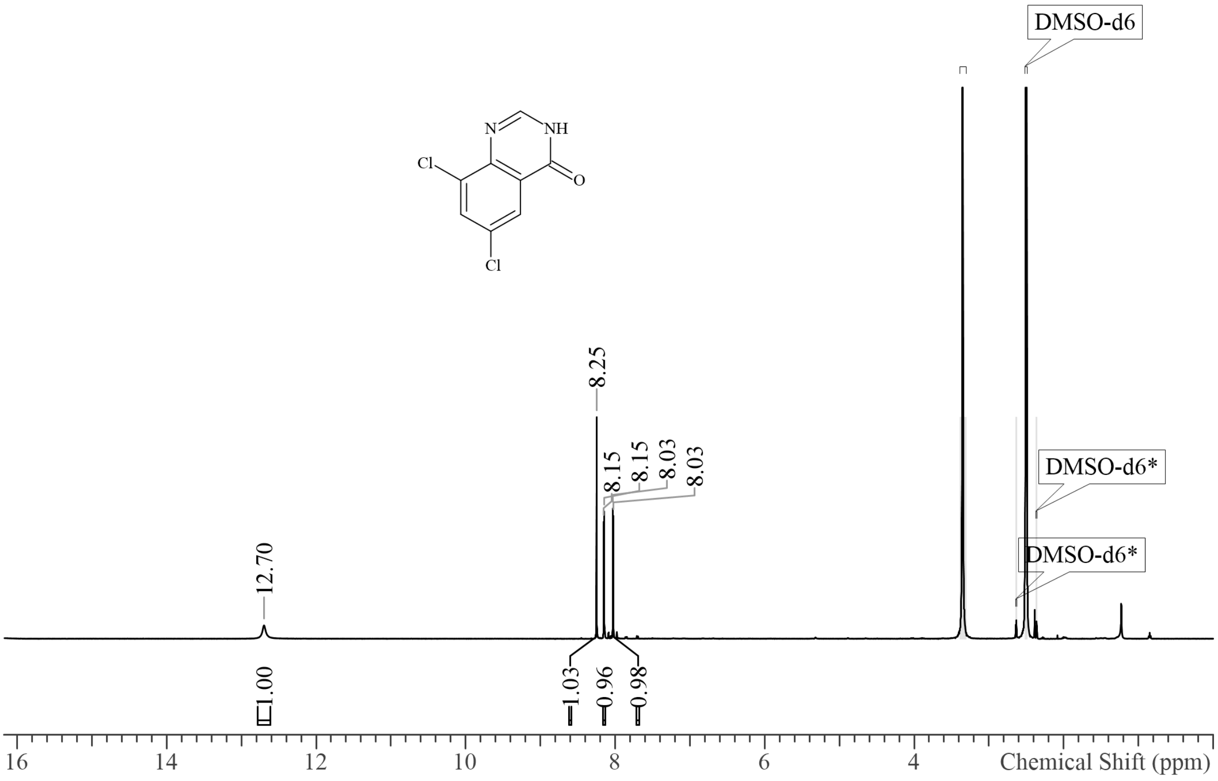

SB-Fig.1: ^{1}H NMR spectrum of 3b in DMSO-d$_6$ at 500 megahertz (MHz).

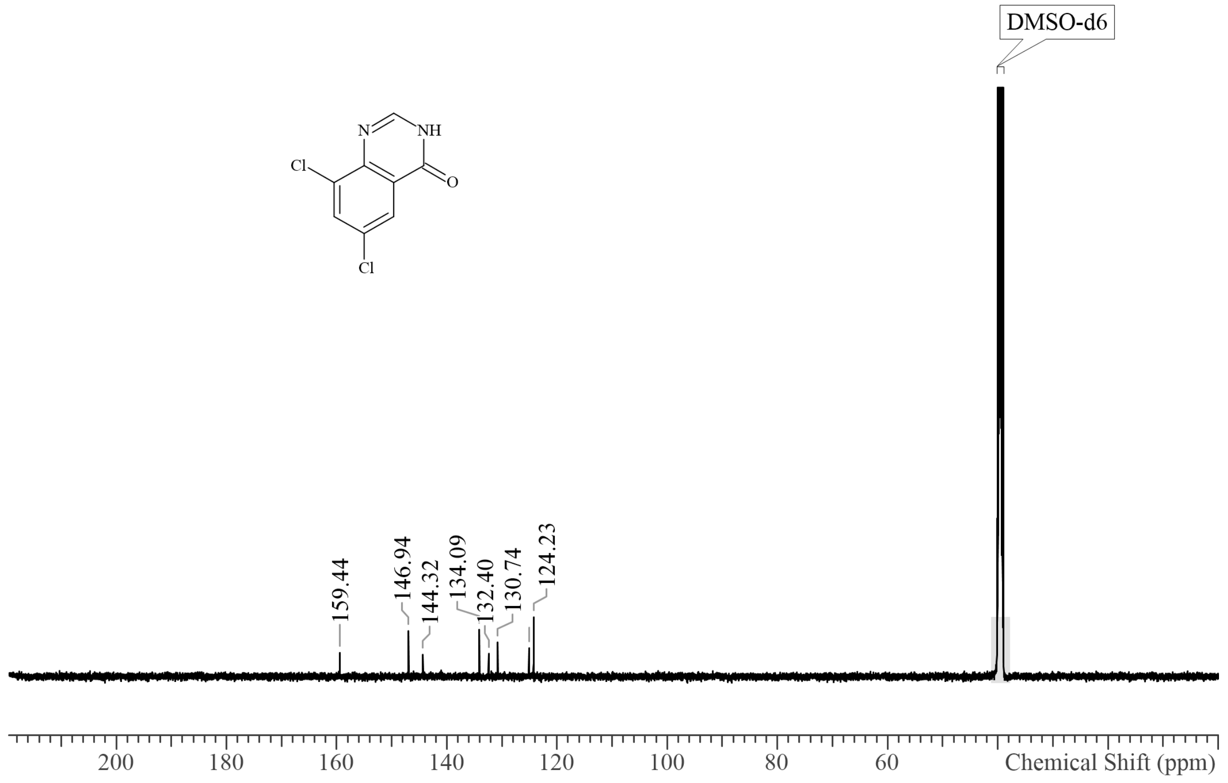

SB-Fig.2: ^{13}C NMR spectrum of 3b in DMSO-d$_6$ at 126 MHz.

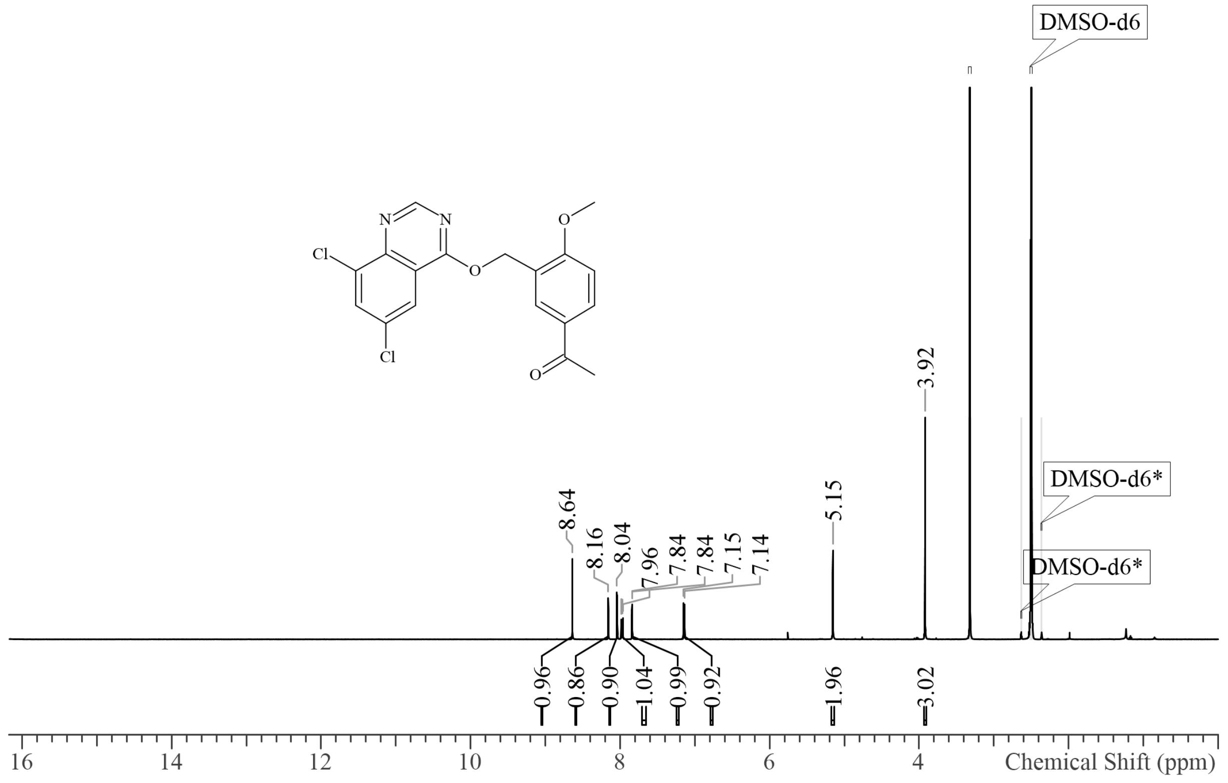

SB-Fig.3: ^{1}H NMR spectrum of 3s recorded in DMSO-d$_6$ at 500 MHz.

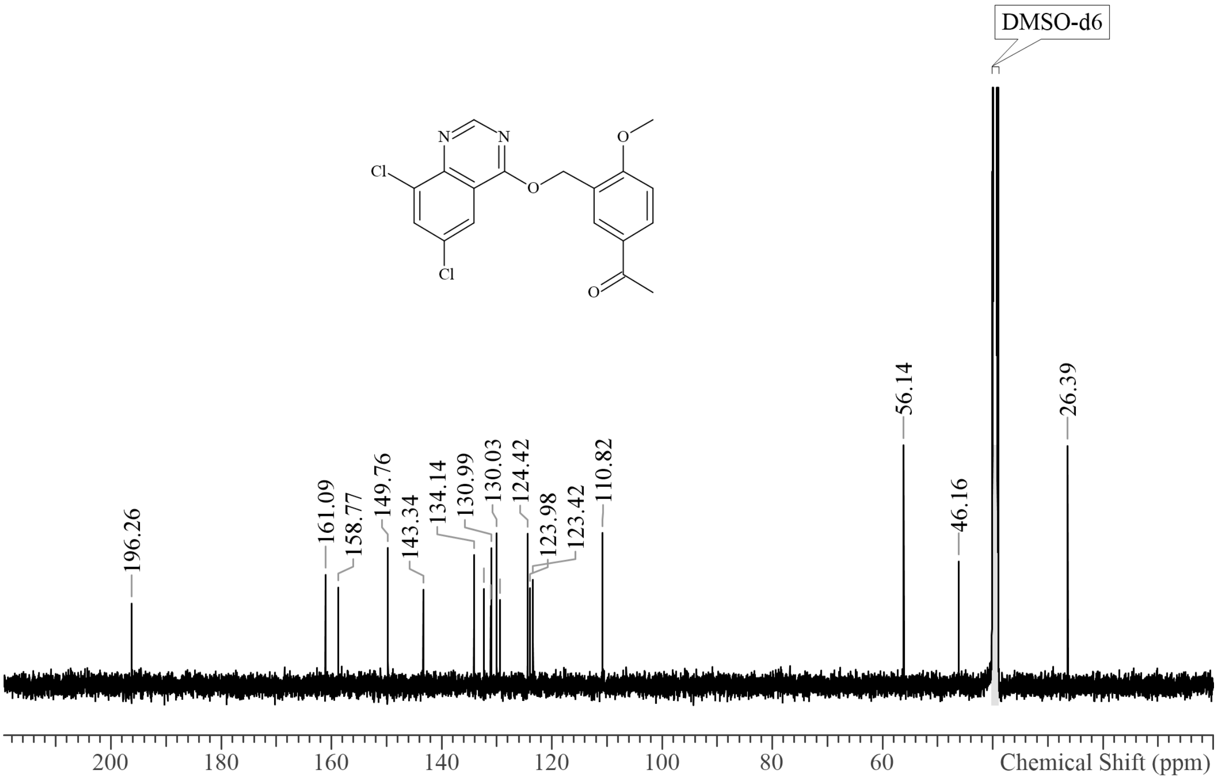

SB-Fig.4: ^{13}C NMR spectrum of 3s recorded in DMSO-d$_6$ at 126 MHz.

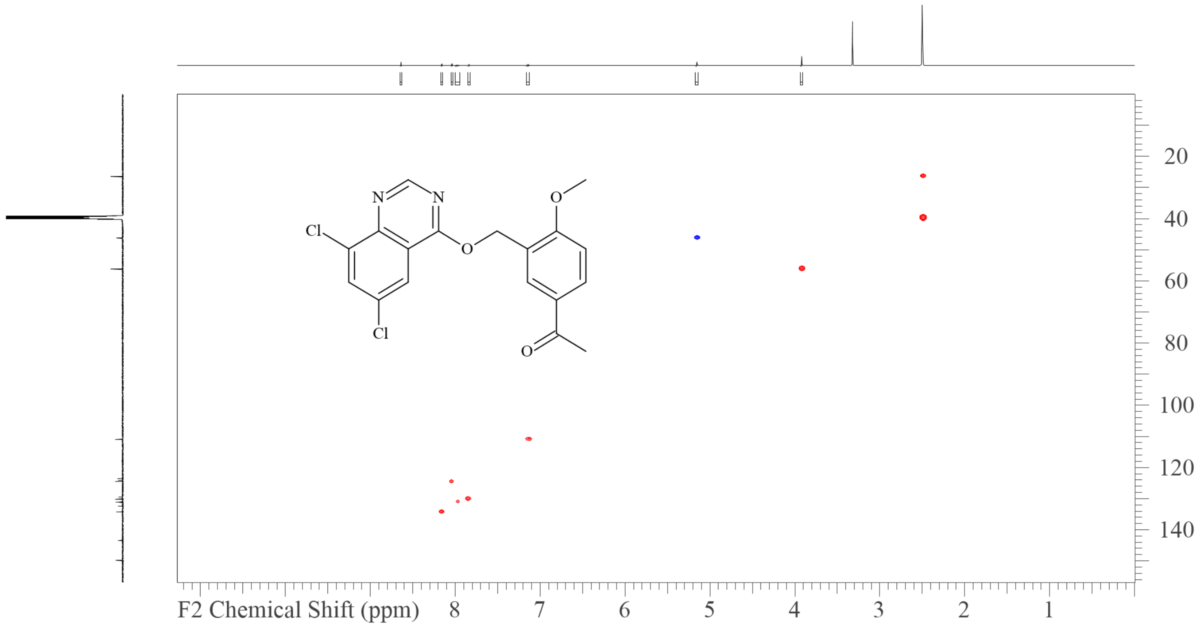

SB-Fig.5: HSQC-2D spectrum of 3s recorded in DMSO-d$_6$.

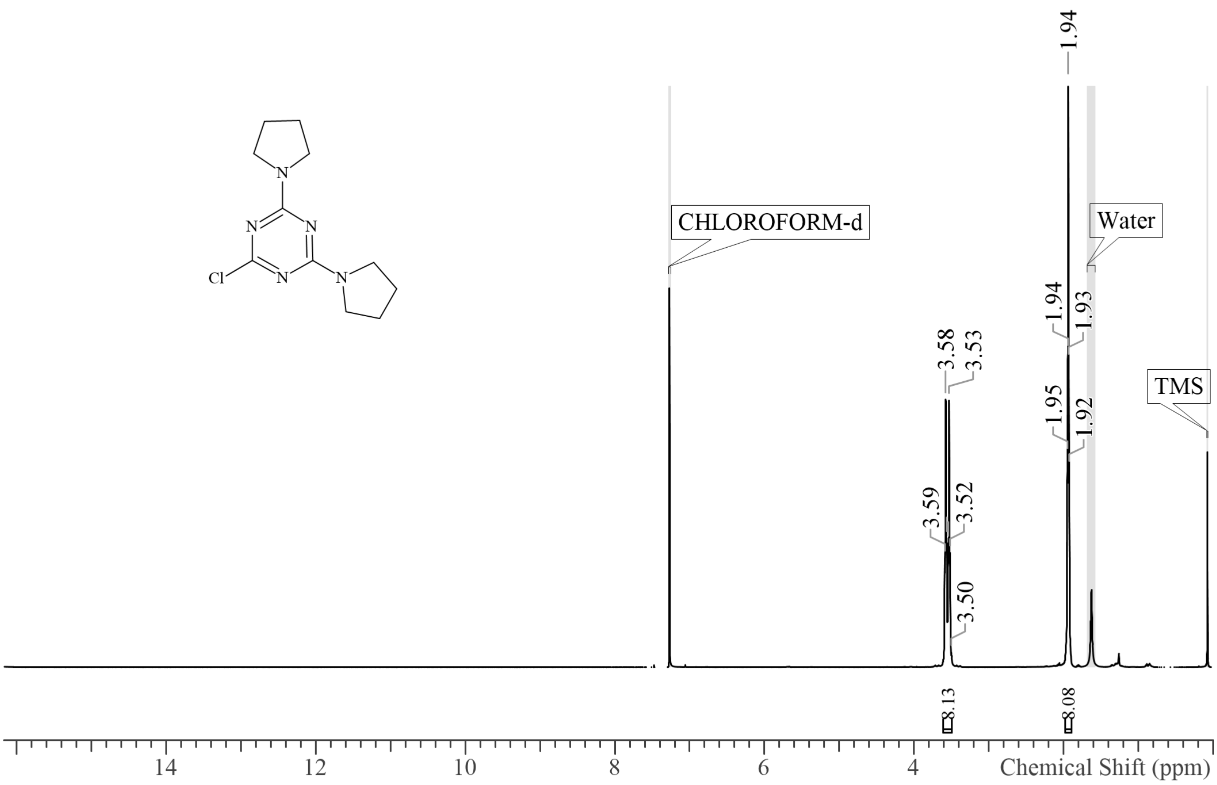

SB-Fig.6: ^{1}H NMR spectrum of 5b recorded in $CDCl_3$ at 500 MHz.

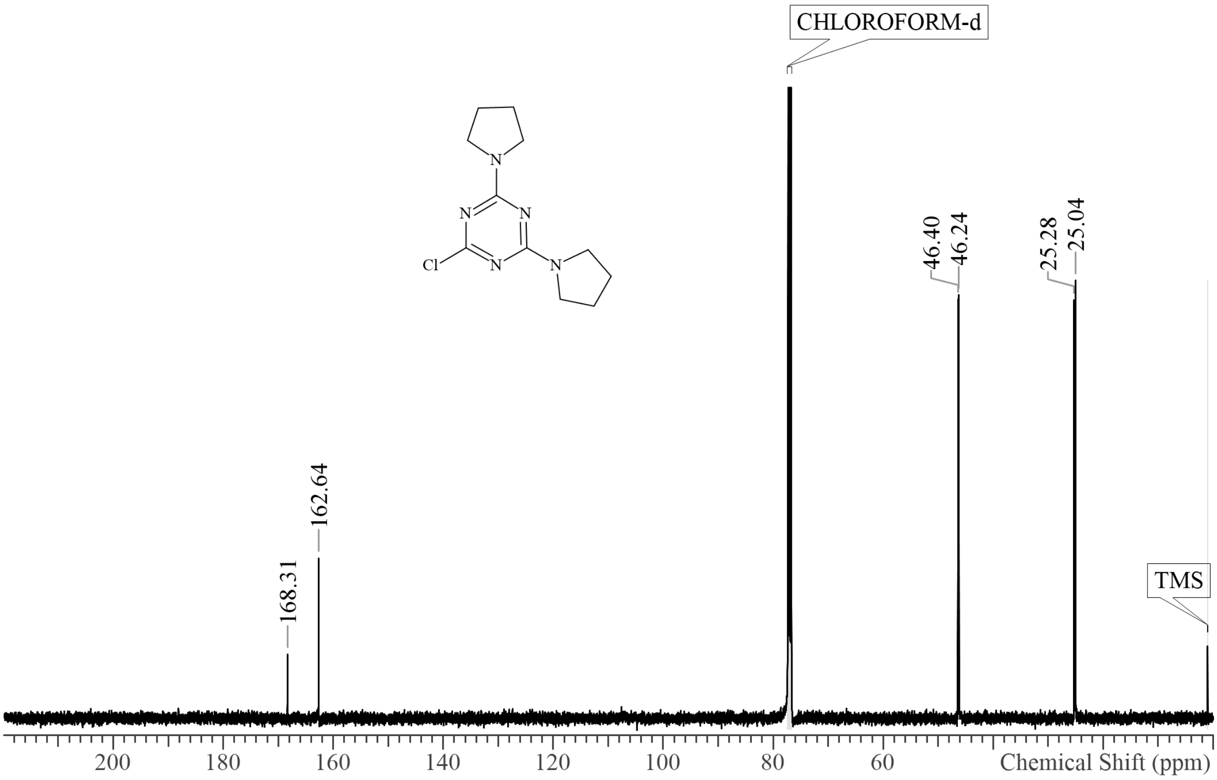

SB-Fig.7: ^{13}C NMR spectrum of 5b recorded in $CDCl_3$ at 126 MHz.

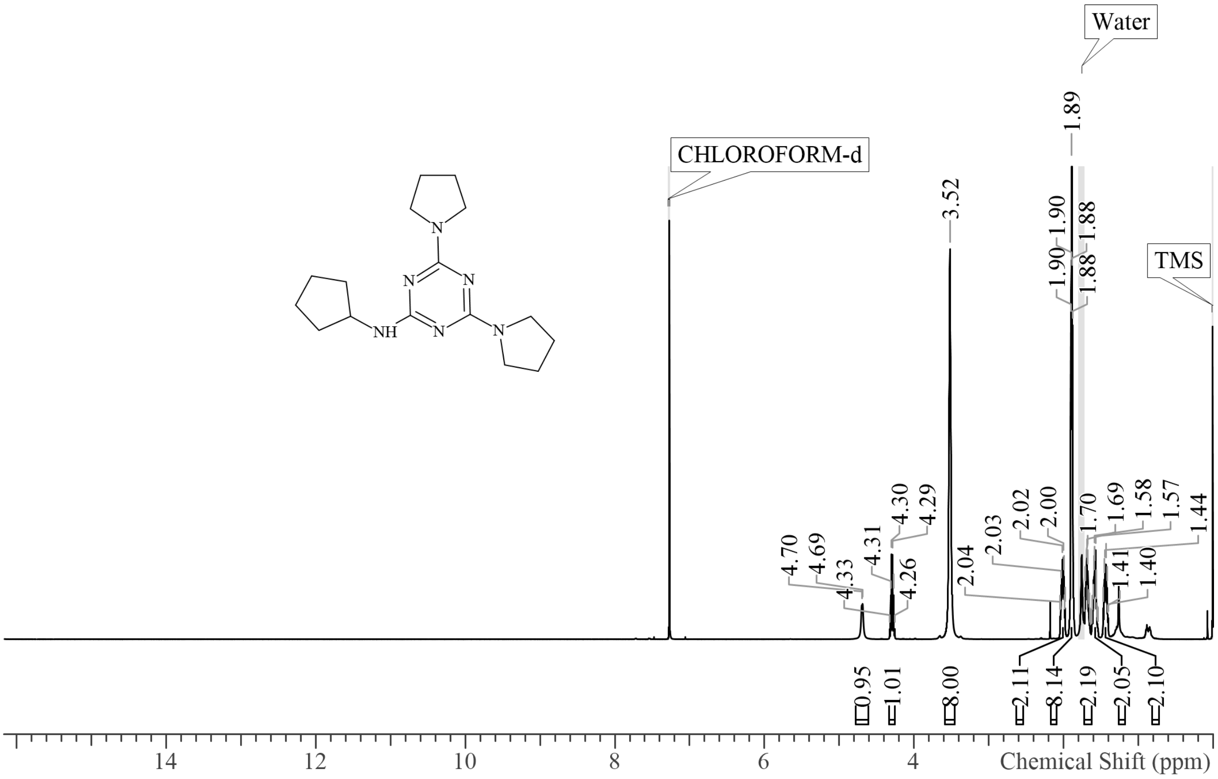

SB-Fig.8: ^{1}H NMR spectrum of 5s recorded in $CDCl_3$ at 500 MHz.

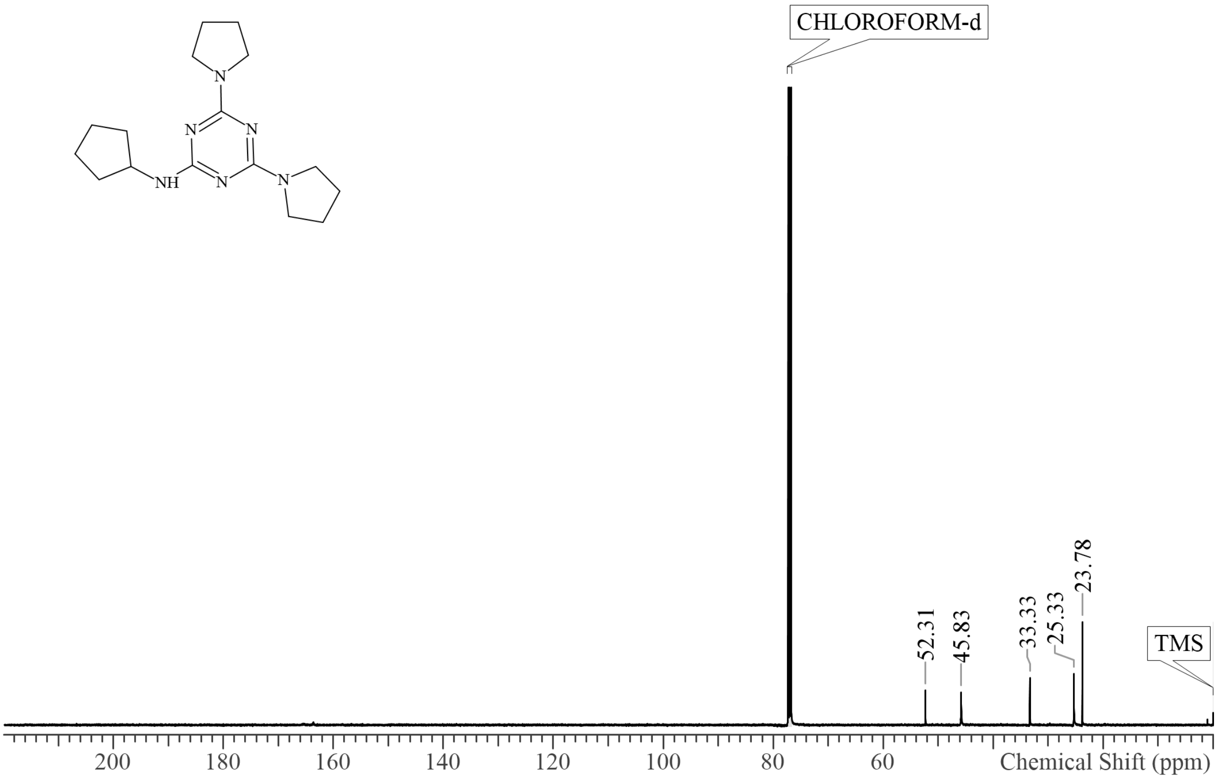

SB-Fig.9: ^{13}C NMR spectrum of 5s recorded in $CDCl_3$ at 126 MHz.

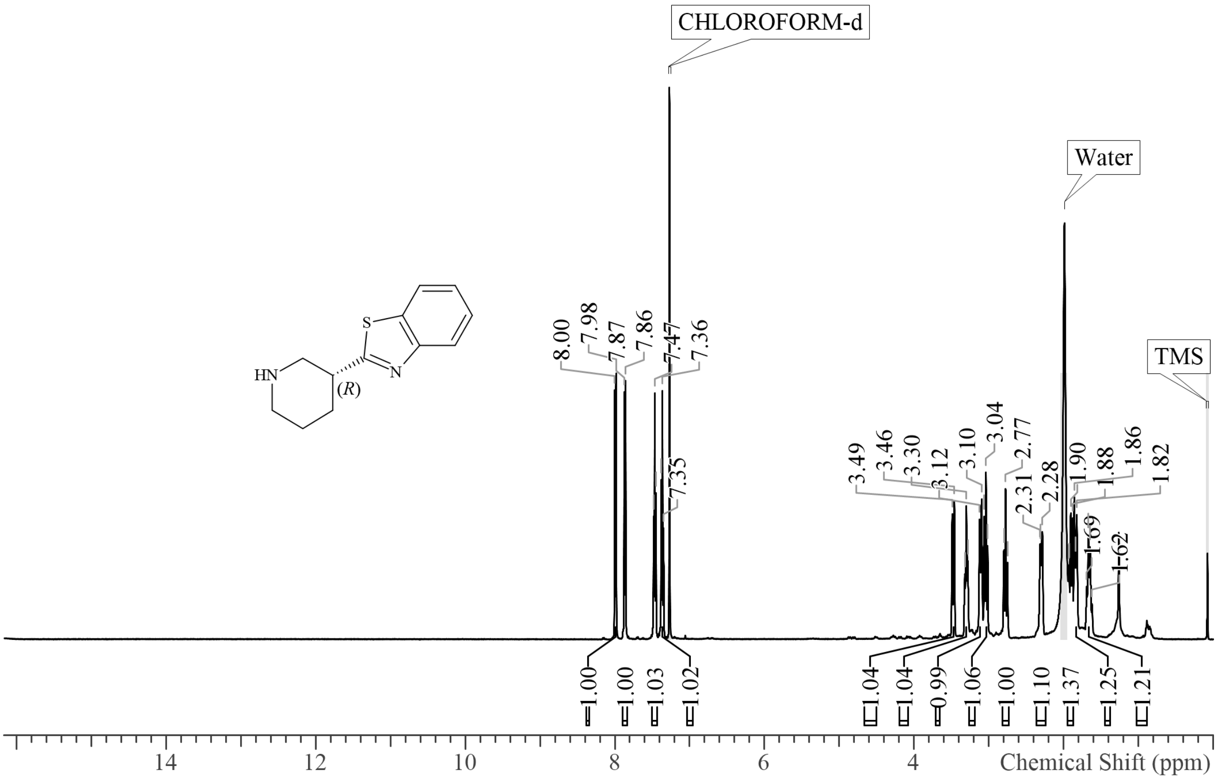

SB-Fig.10: ^{1}H NMR spectrum of 9b-1 recorded in $CDCl_3$ at 500 MHz.

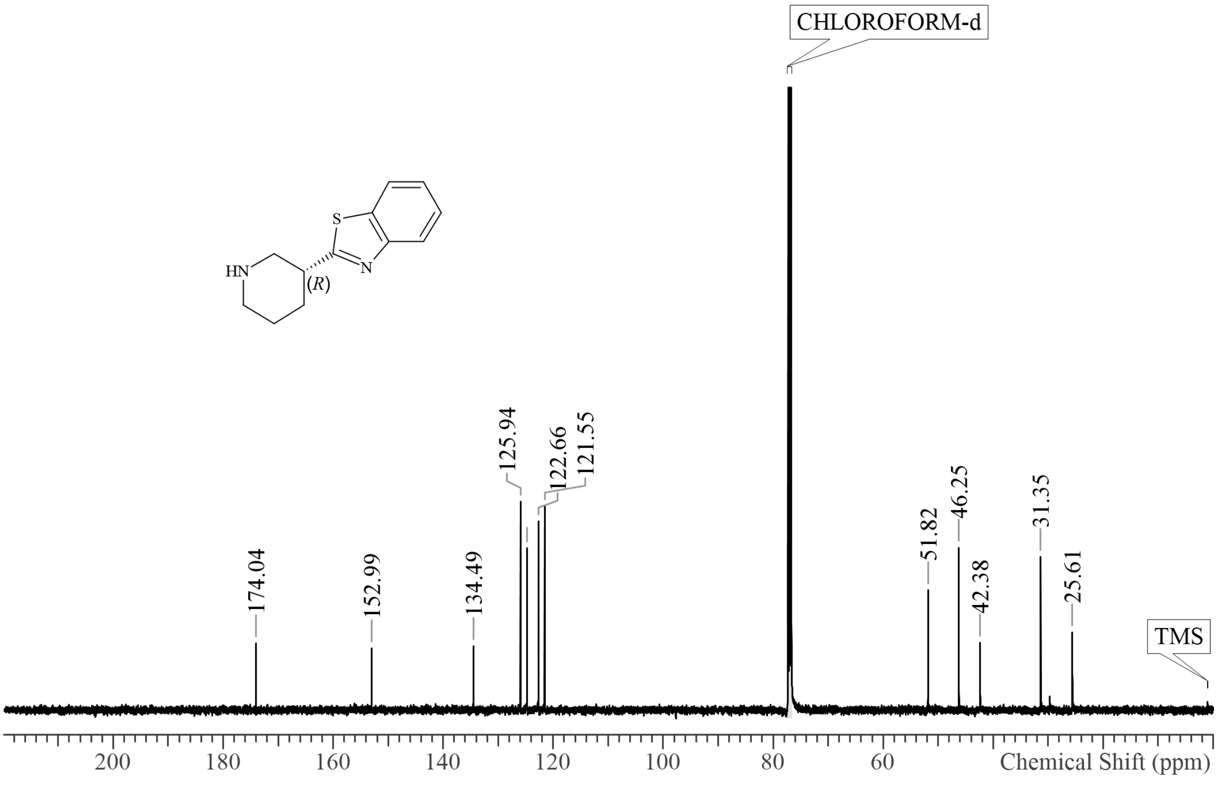

SB-Fig.11: ^{13}C NMR spectrum of 9b-1 recorded in $CDCl_3$ at 126 MHz.

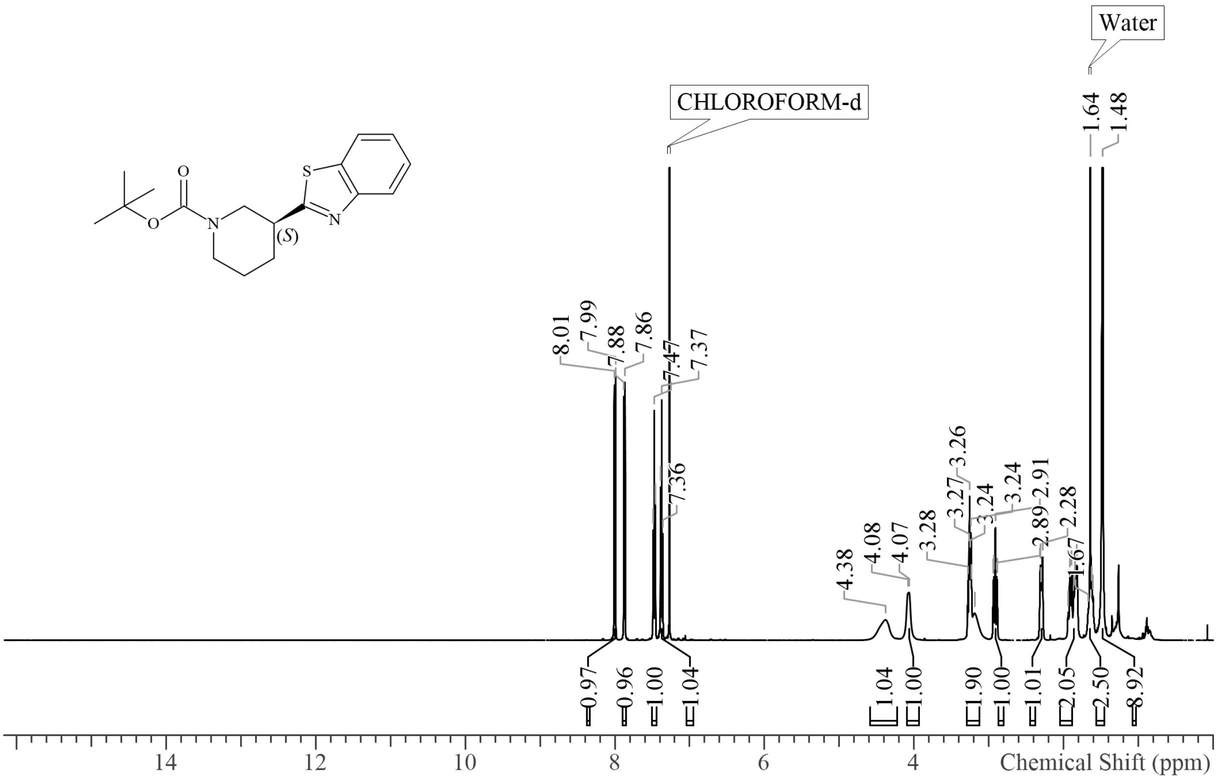

SB-Fig.12: ^{1}H NMR spectrum of 9b-2 recorded in $CDCl_3$ at 500 MHz.

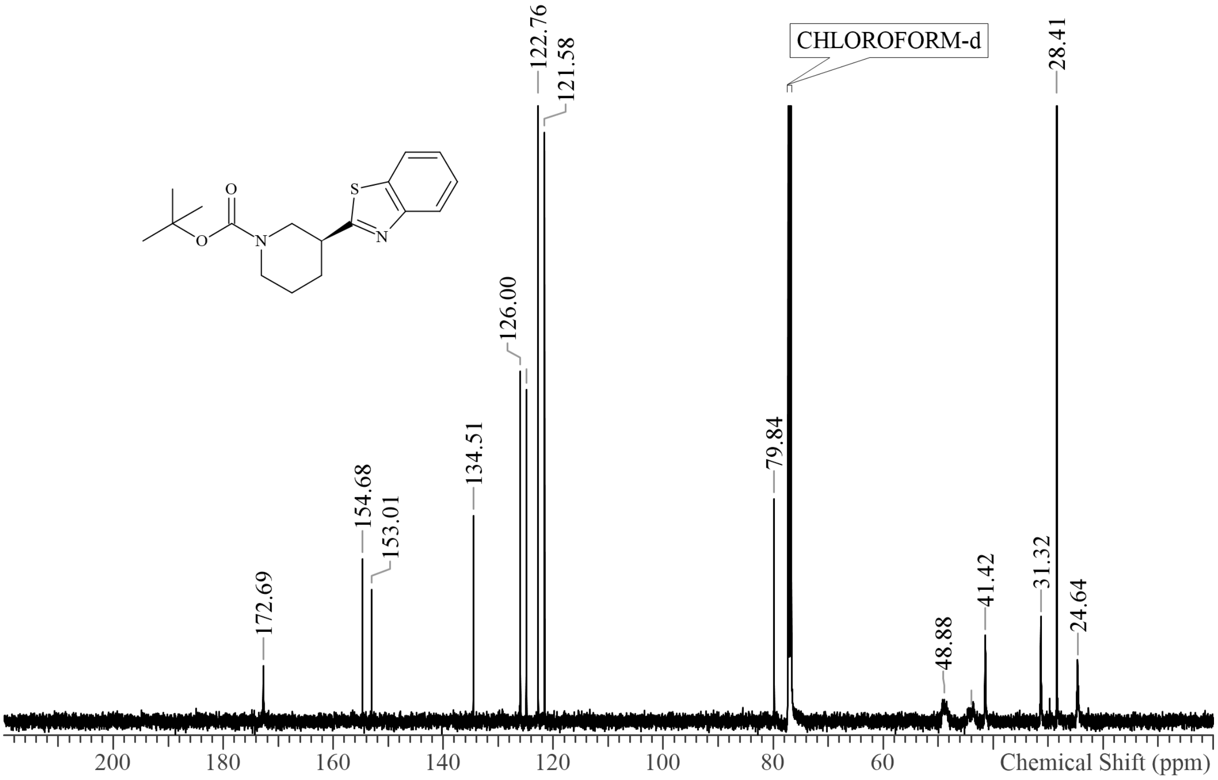

SB-Fig.13: ^{13}C NMR of 9b-2 recorded in $CDCl_3$ at 126 MHz.

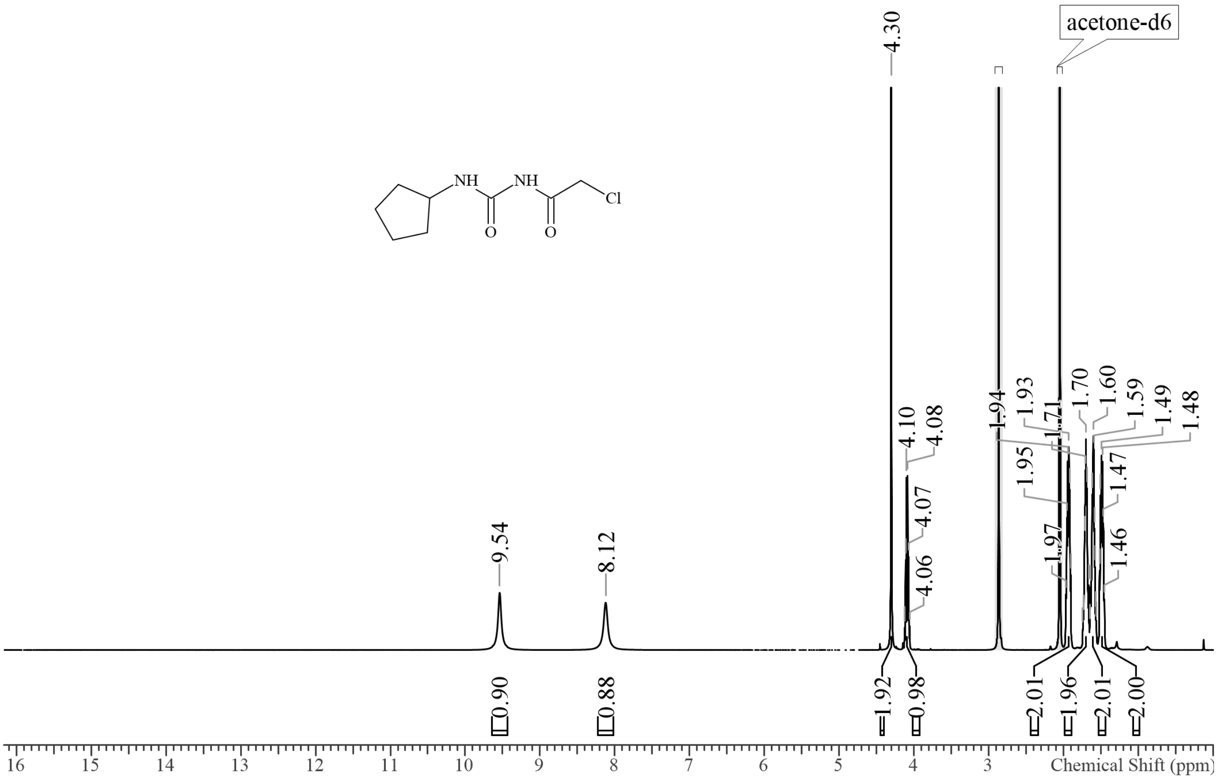

SB-Fig.14: ^{1}H NMR spectrum of 9d recorded in acetone-d_6 at 500 MHz.

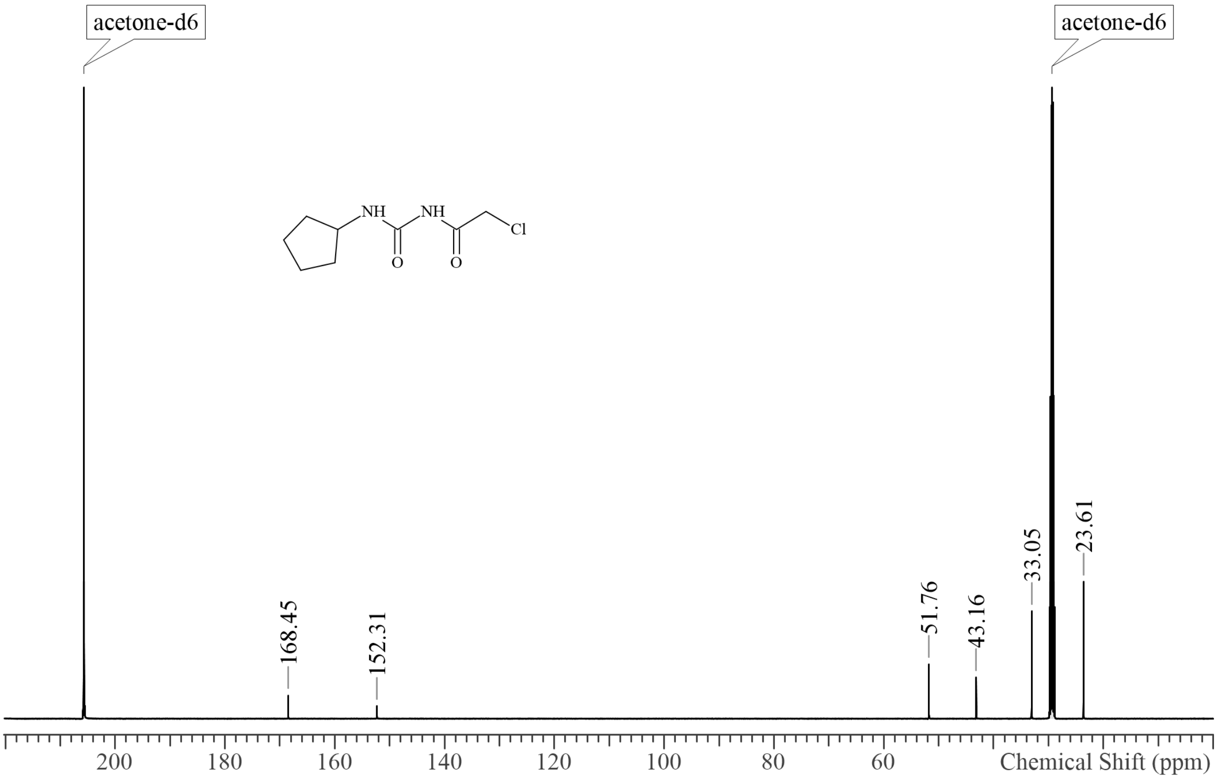

SB-Fig.15: ^{13}C NMR spectrum of 9d recorded in acetone-d_6 at 126 MHz.

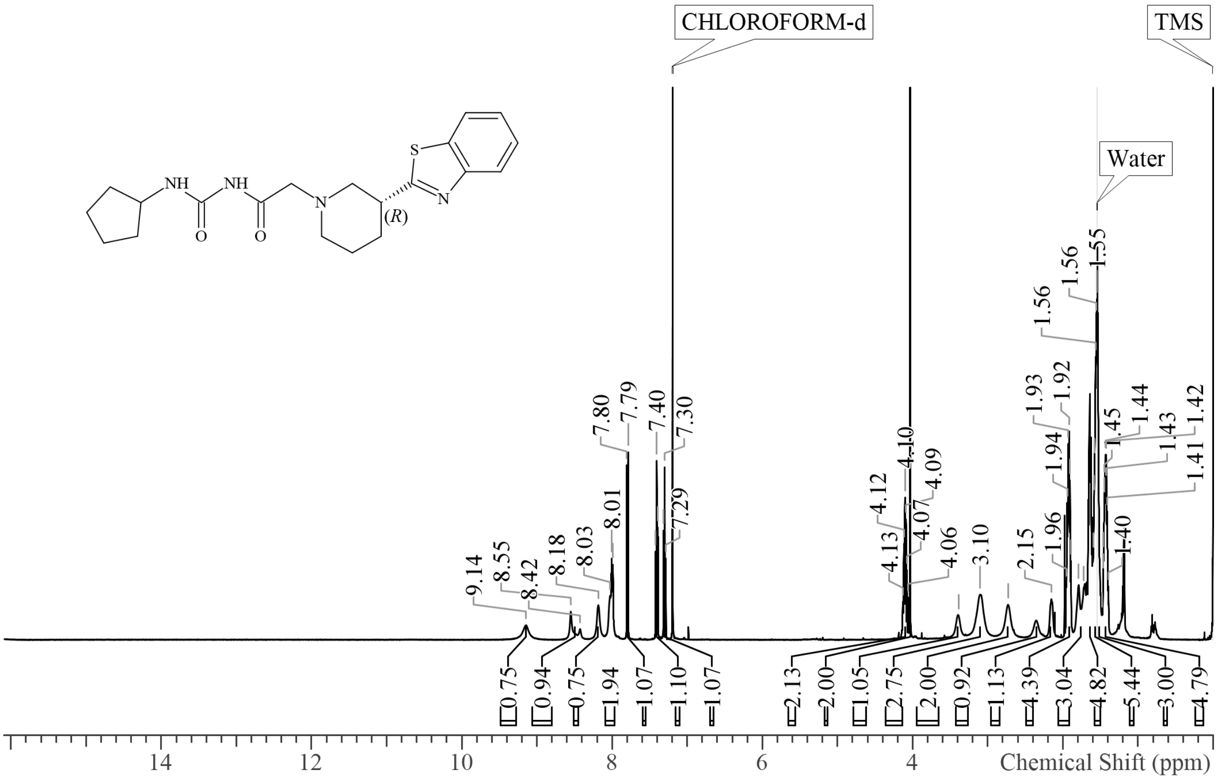

SB-Fig.16: ^{1}H NMR spectrum of (*R*)-9s recorded in $CDCl_3$ at 500 MHz.

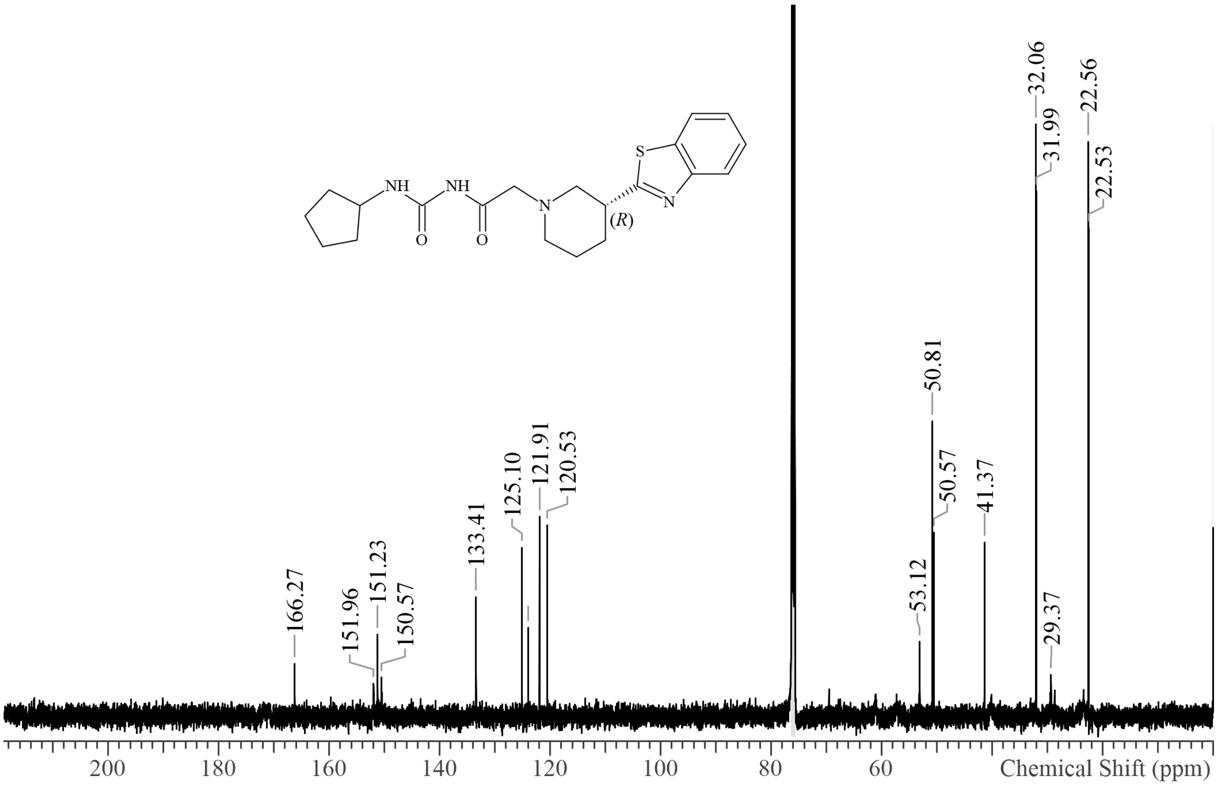

SB-Fig.17: ^{13}C NMR spectrum of (*R*)-9s recorded in $CDCl_3$ at 126 MHz.

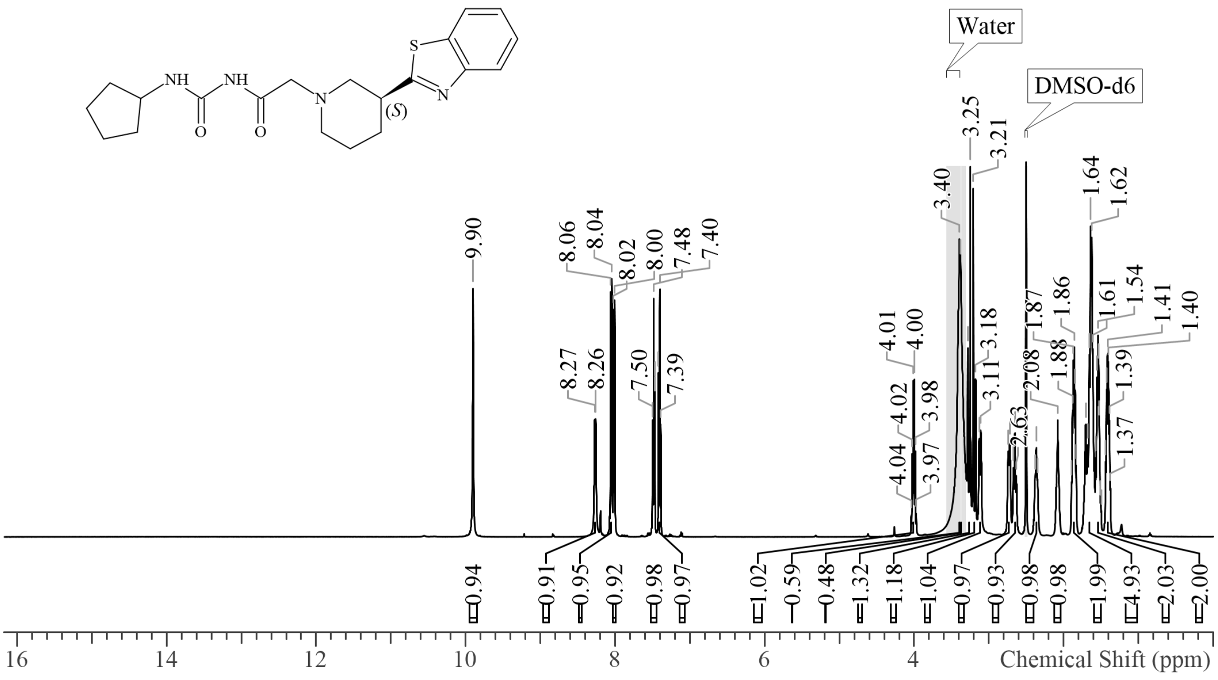

SB-Fig.18: ^{1}H NMR spectrum of (*S*)-9s recorded in DMSO-d$_6$ at 500 MHz.

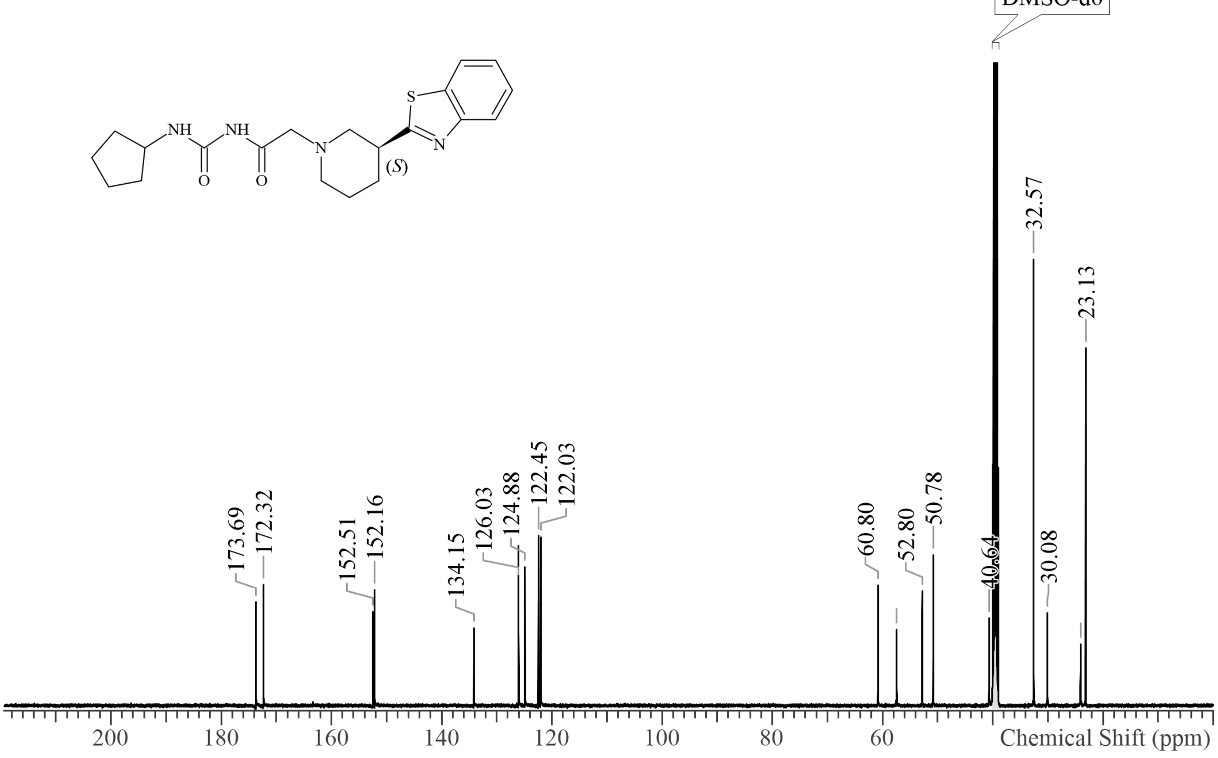

SB-Fig.19: ^{13}C NMR spectrum of (*S*)-9s measured in DMSO-d$_6$ at 126 MHz.

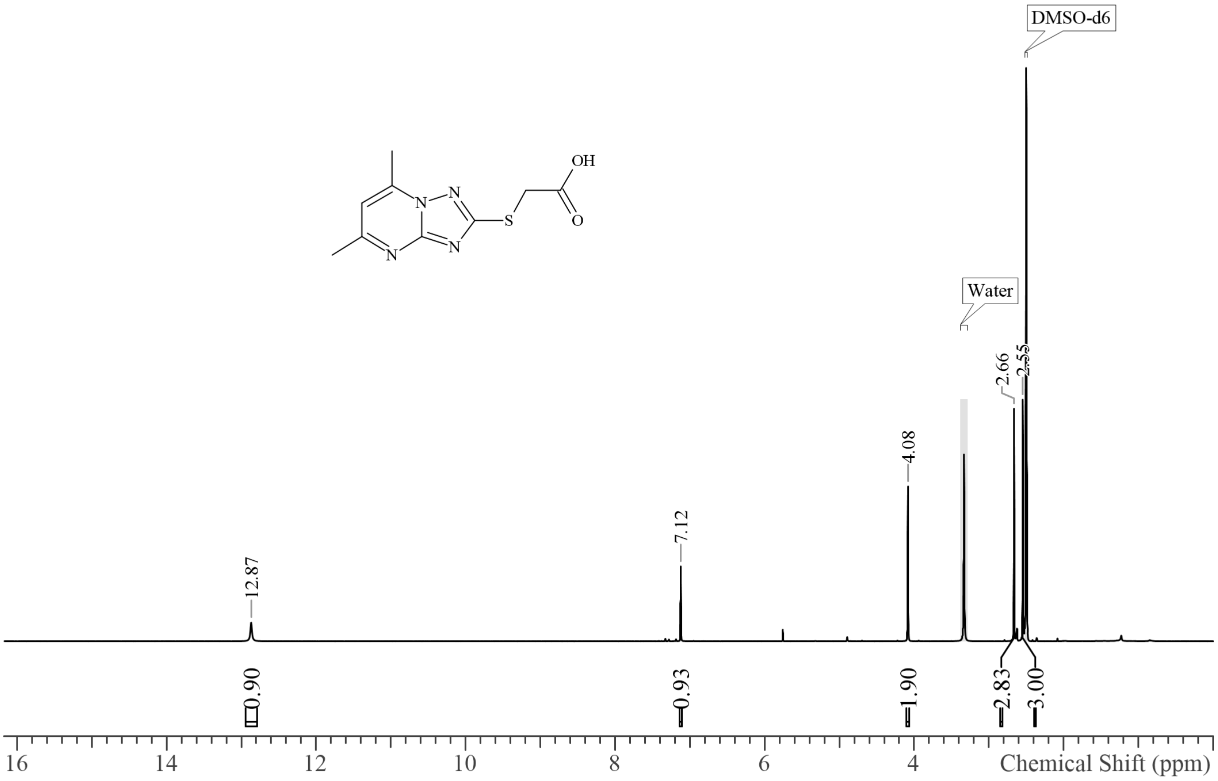

SB-Fig.20: ^{1}H NMR spectrum of 12b measured in DMSO-d_6 at 500 MHz.

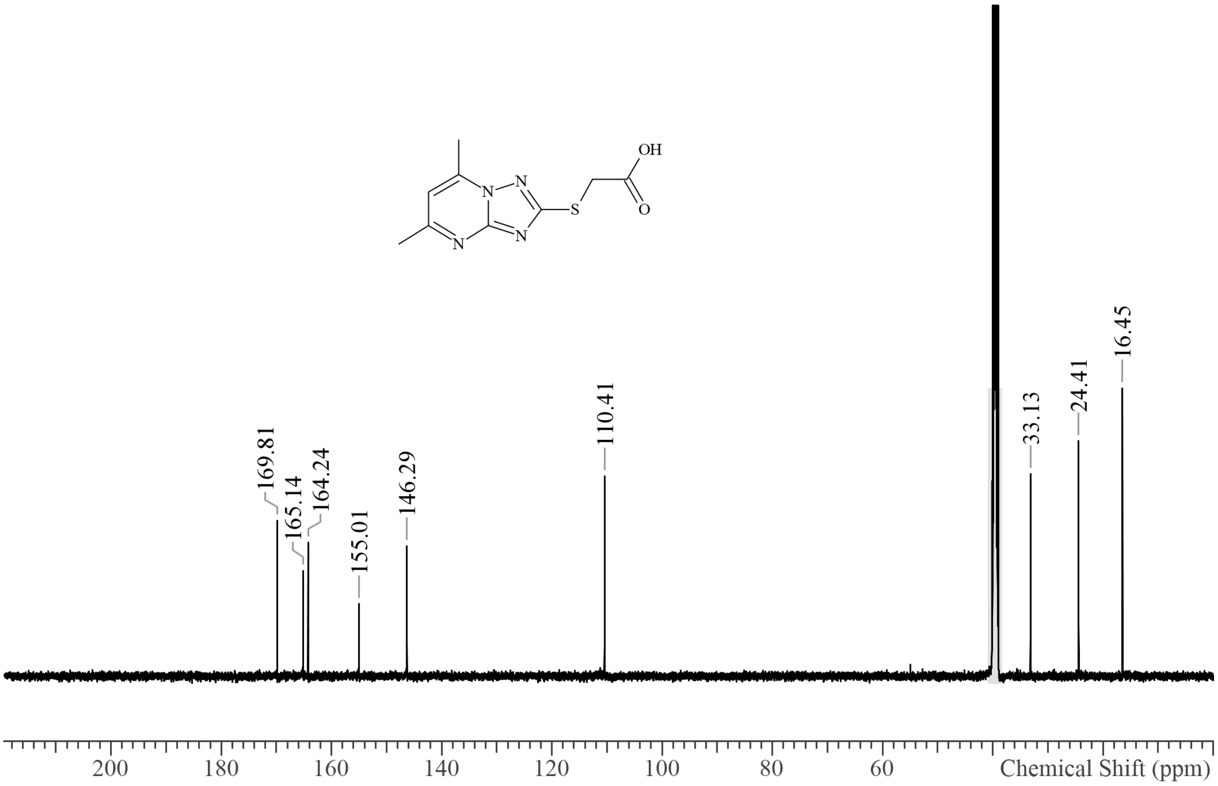

SB-Fig.21: ^{13}C NMR spectrum of 12b measured in DMSO-d_6 at 126 MHz.

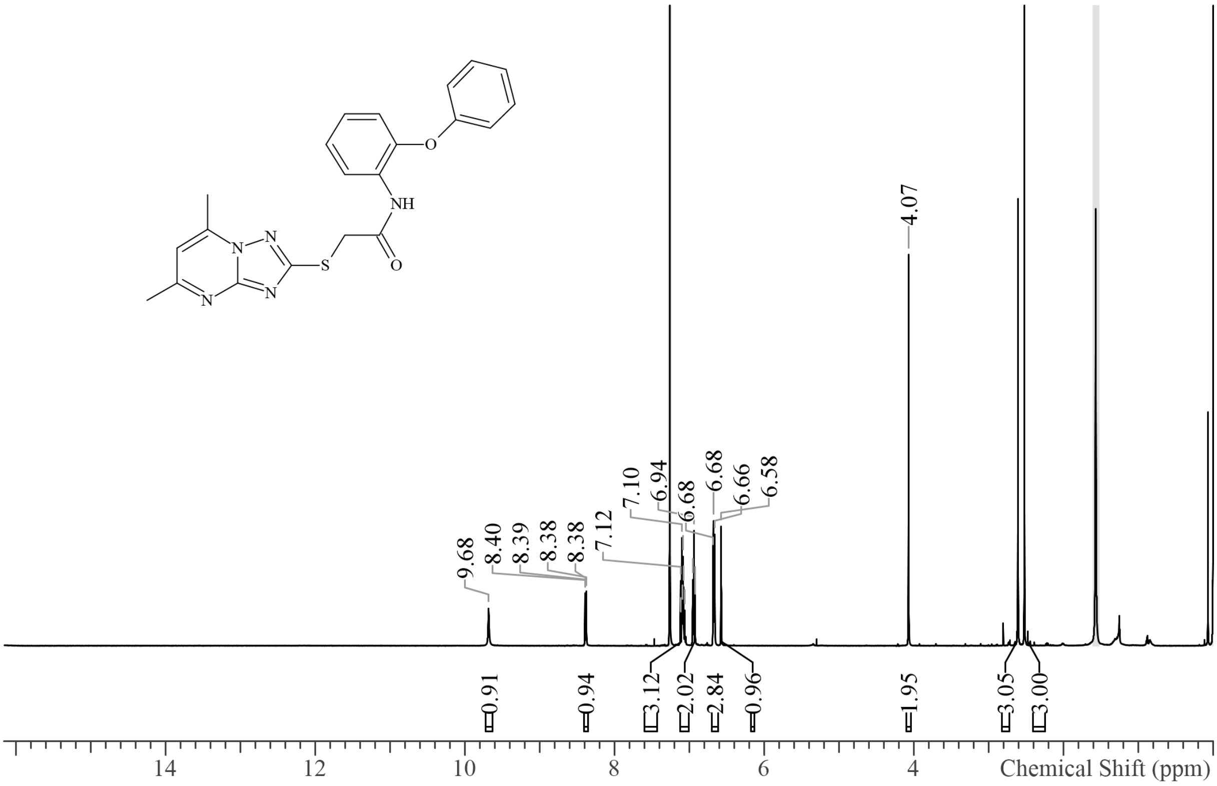

SB-Fig.22: ^{1}H NMR spectrum of 12s measured in $CDCl_3$ at 500 MHz.

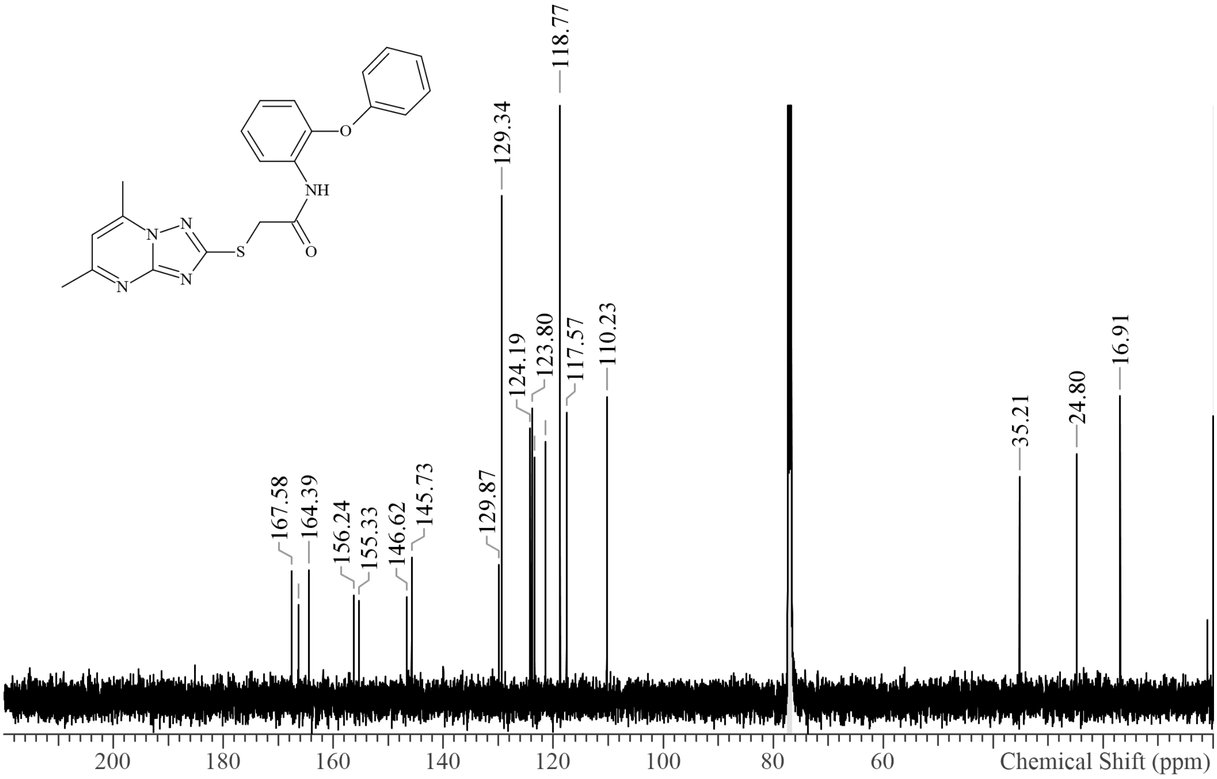

SB-Fig.23: ^{13}C NMR spectrum of 12s measured in $CDCl_3$ at 126 MHz.

4.2 LC-MS data

4.2.1 Characterizations/purity analyses of intermediates and final compounds

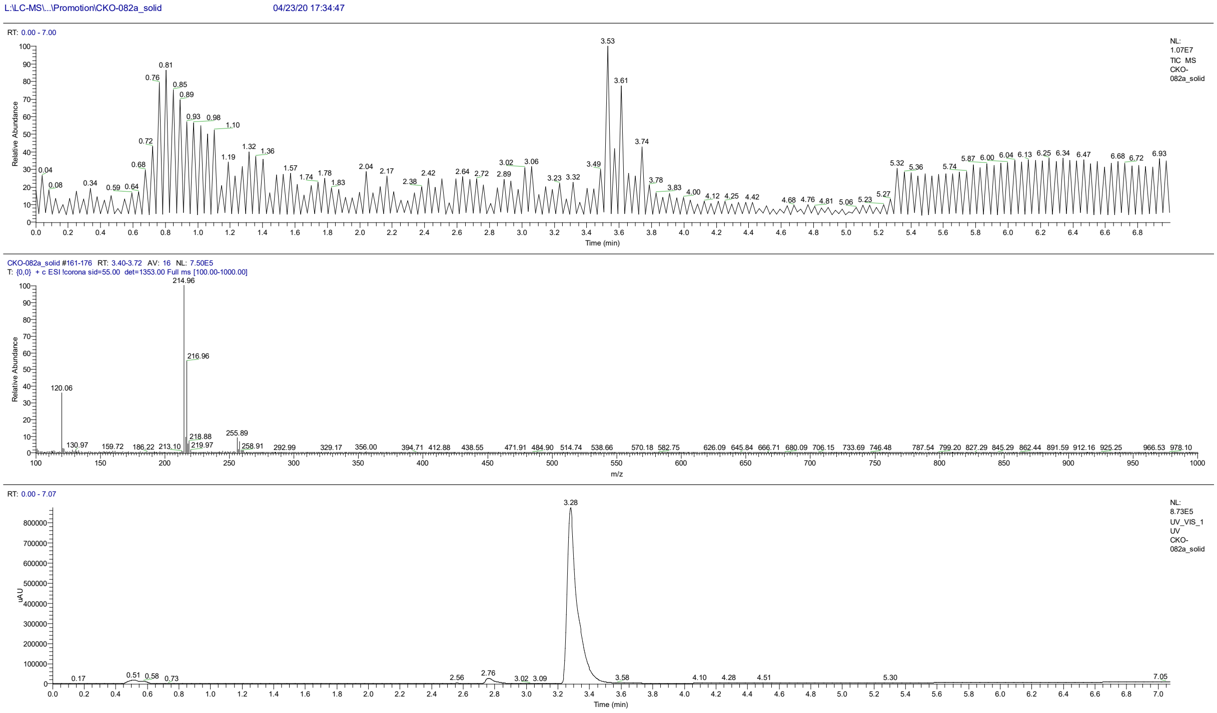

SB-Fig.24: LC-MS data of 3b (top: mass chromatogram, middle: mass spectrum of UV/Vis peak, bottom: UV/Vis chromatogram).

SB-Table 1: Peak list of UV/Vis chromatogram for purity analysis of 3b.

Apex RT	Start RT	End RT	Area	%Area	Height	%Height
2.76	2.73	2.85	80737.208	2.02	23779.566	2.66
3.28	3.23	3.57	3917181.870	97.98	868548.459	97.34

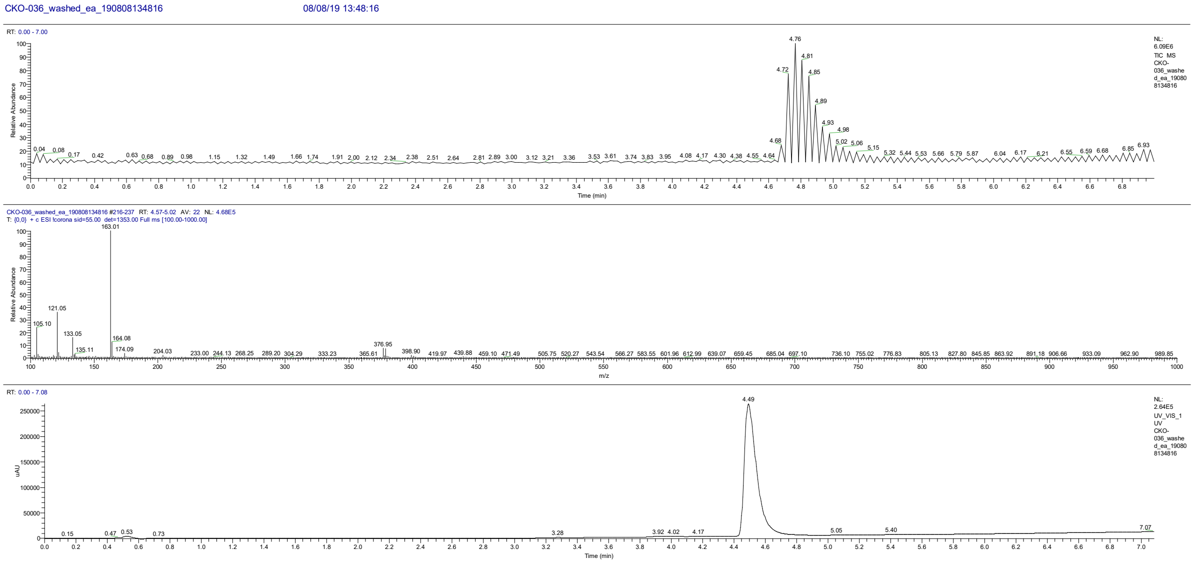

SB-Fig.25: LC-MS data of 3s (top: mass chromatogram, middle: mass spectrum of UV/Vis peak, bottom: UV/Vis chromatogram).

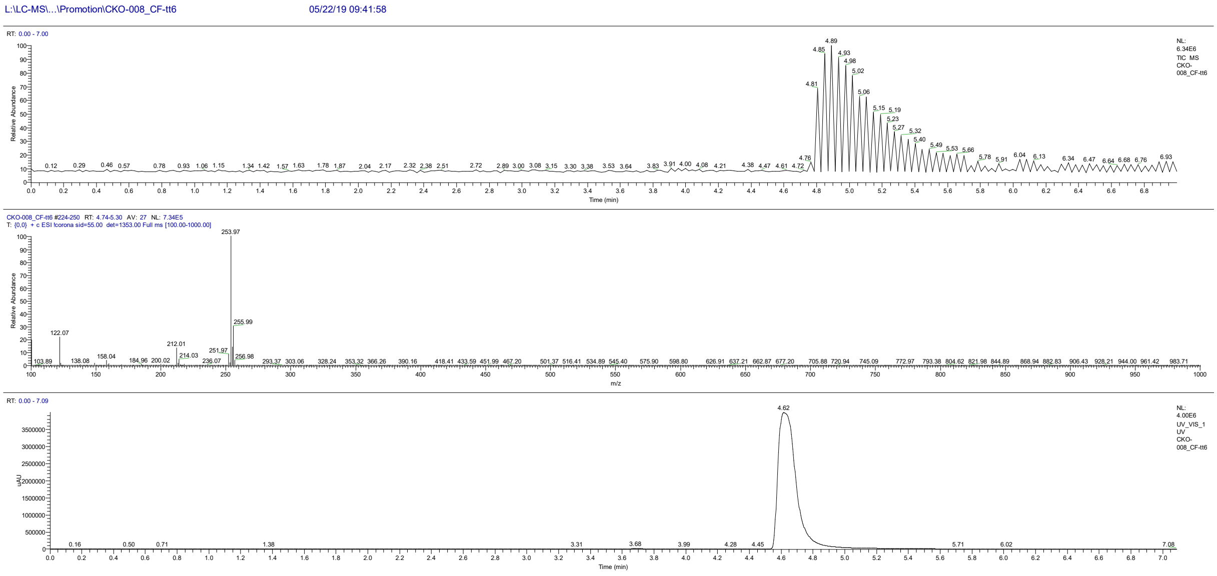

SB-Fig.26: LC-MS data of 5b (top: mass chromatogram, middle: mass spectrum of UV/Vis peak, bottom: UV/Vis chromatogram).

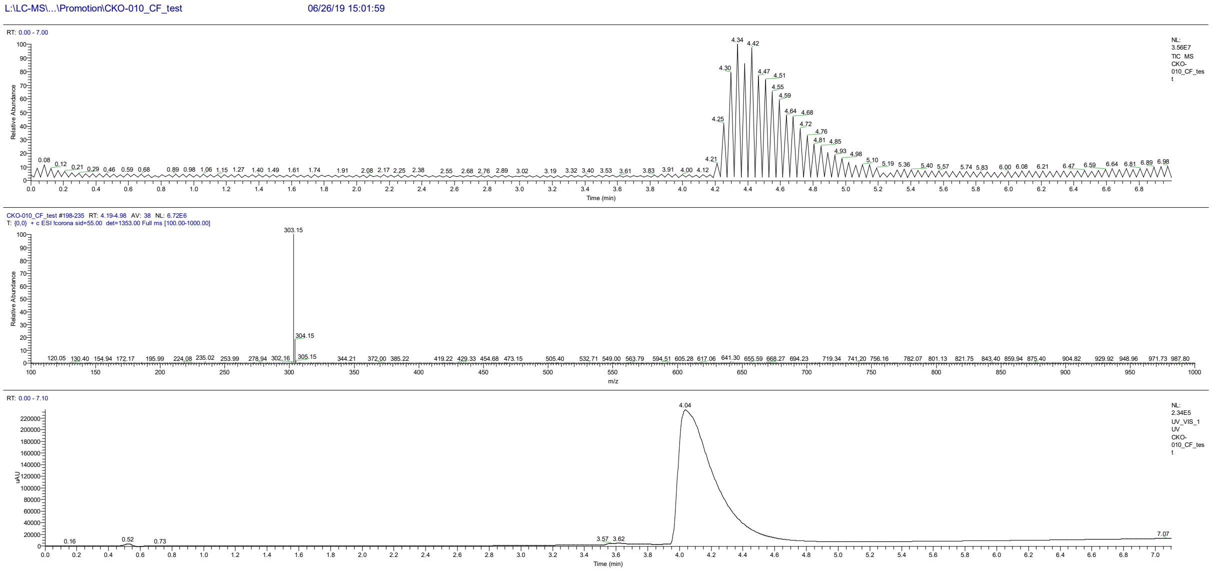

SB-Fig.27: LC-MS data of 5s (top: mass chromatogram, middle: mass spectrum of main UV/Vis peak, bottom: UV/Vis chromatogram).

SB-Table 2: Peak list of UV/Vis chromatogram for purity analysis of 5s.

Apex RT	Start RT	End RT	Area	%Area	Height	%Height
3.62	3.53	3.87	21430.153	0.59	2403.576	1.03
4.04	3.94	4.74	3587255.601	99.41	229963.617	98.97

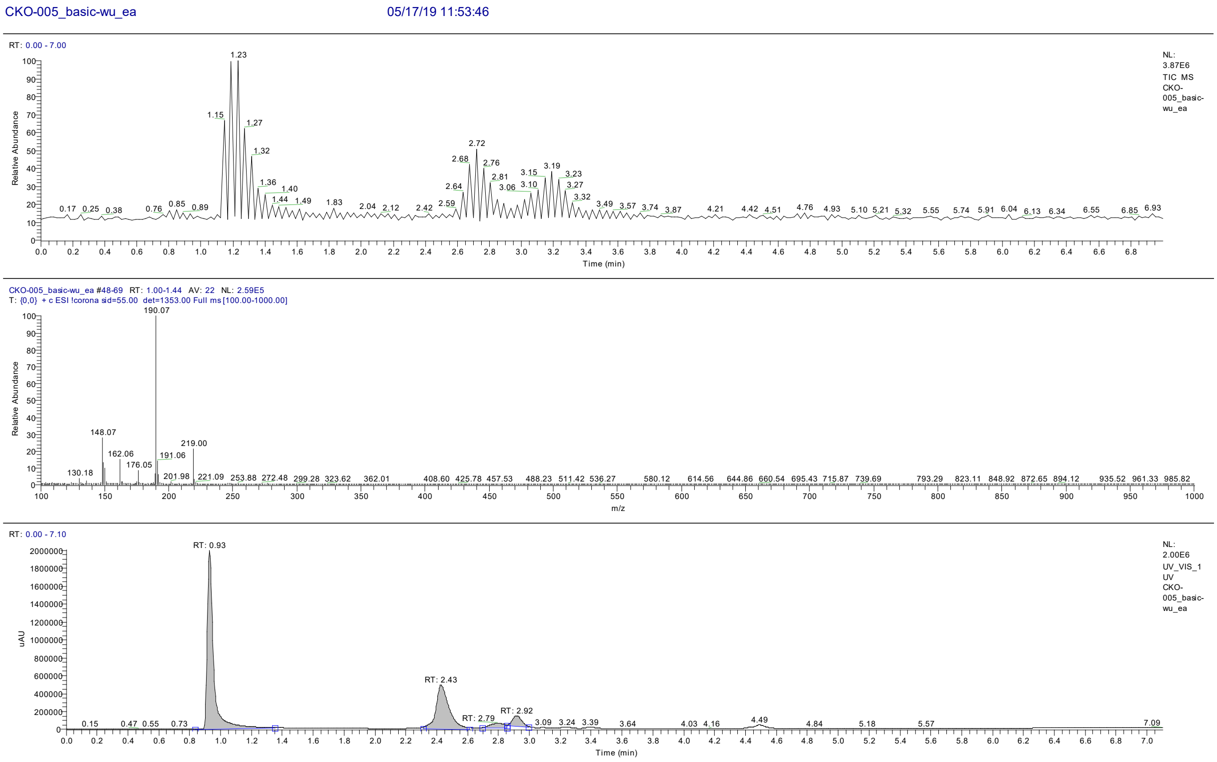

SB-Fig.28: LC-MS data of 9b-1 (top: mass chromatogram, middle: mass spectrum of main UV/Vis peak, bottom: UV/Vis chromatogram).

SB-Table 3: Peak list of UV/Vis chromatogram for purity analysis of 9b-1.

Apex RT	Start RT	End RT	Area	%Area	Height	%Height
0.93	0.84	1.35	5671055.252	61.76	1998212.286	75.87
2.43	2.31	2.61	2818722.123	30.70	484777.726	18.41
2.79	2.70	2.85	264549.408	2.88	42706.931	1.62
2.92	2.86	2.99	427880.252	4.66	107870.408	4.10

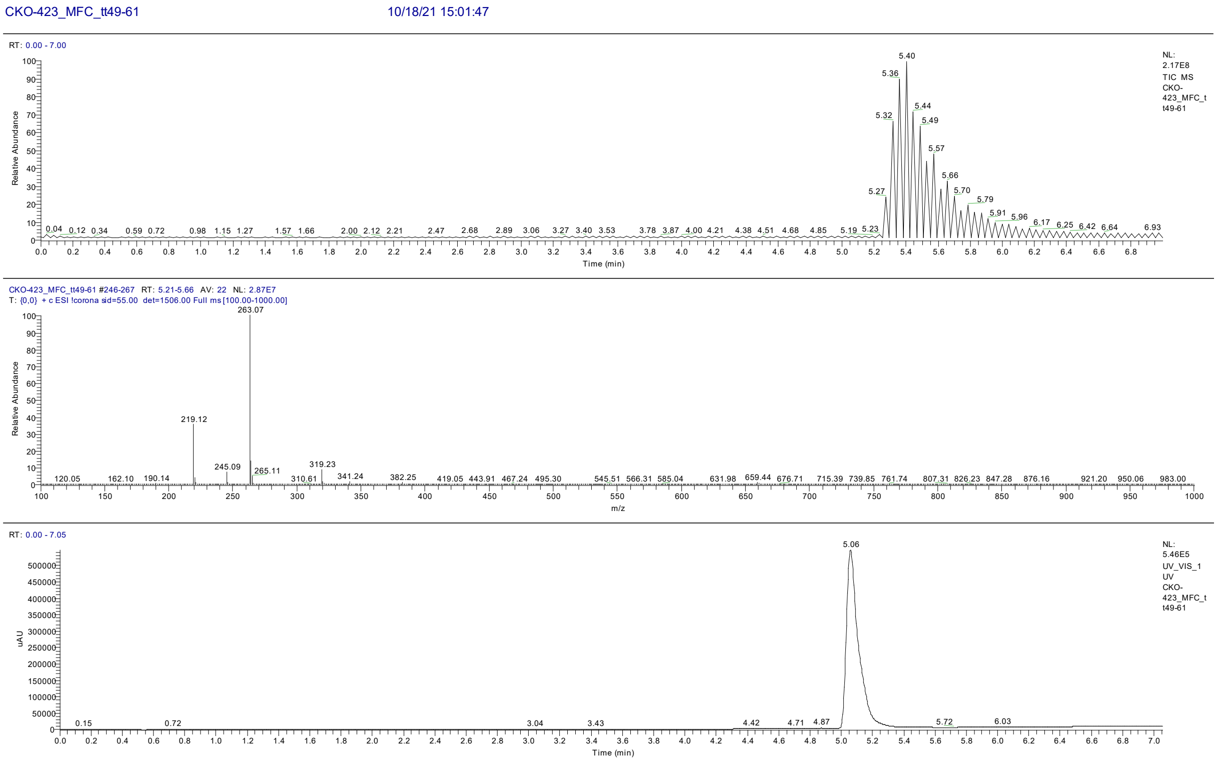

SB-Fig.29: LC-MS data of 9b-2 (top: mass chromatogram, middle: mass spectrum of UV/Vis peak, bottom: UV/Vis chromatogram).

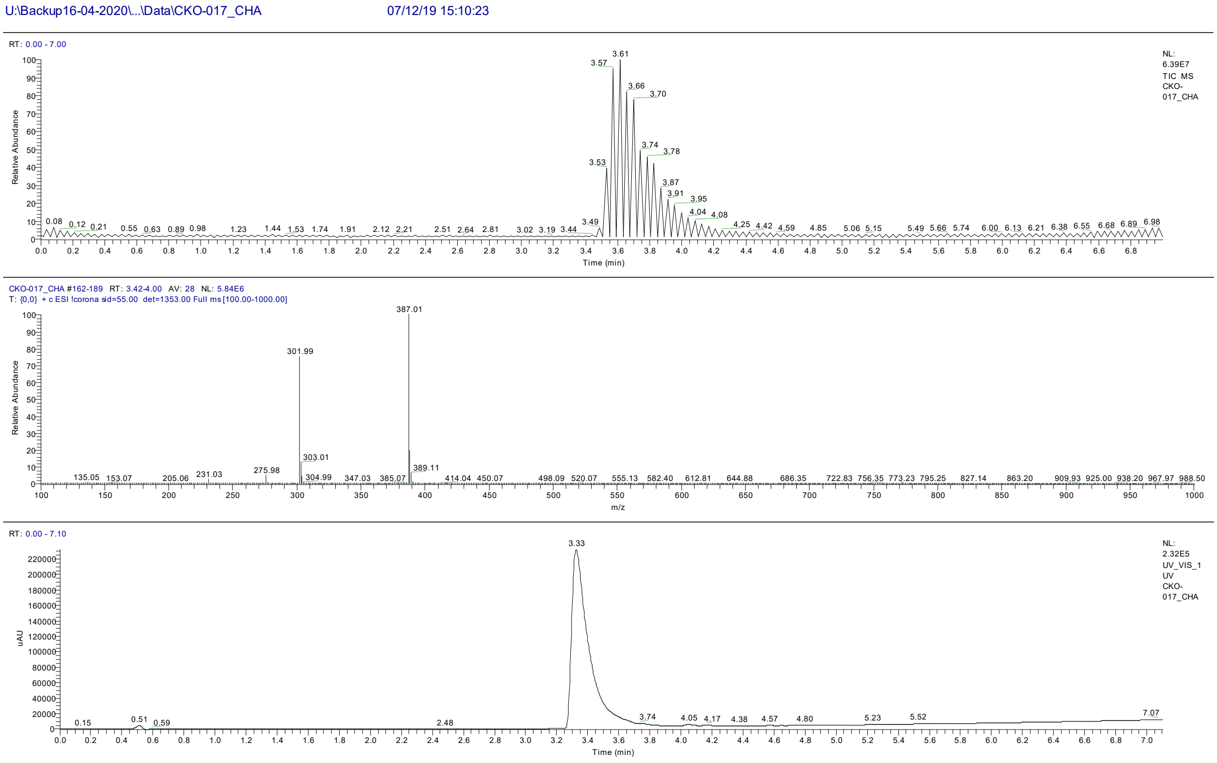

SB-Fig.30: LC-MS data of (*R*)-9s (top: mass chromatogram, middle: mass spectrum of UV/Vis peak, bottom: UV/Vis chromatogram).

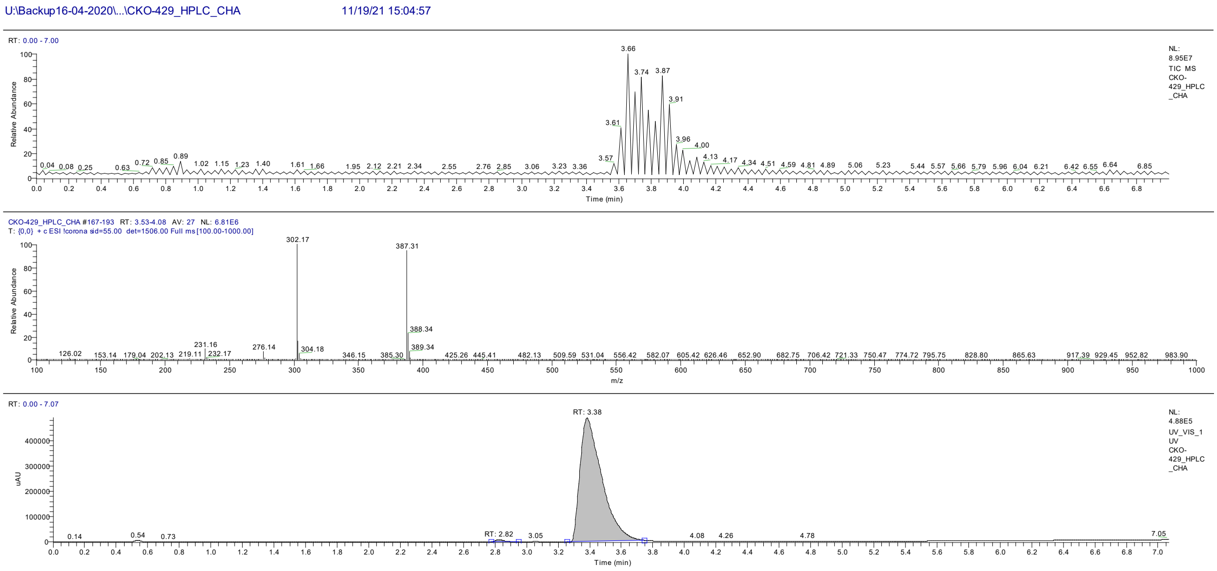

SB-Fig.31: LC-MS data of (*S*)-9s (top: mass chromatogram, middle: mass spectrum of main UV/Vis peak, bottom: UV/Vis chromatogram).

SB-Table 4: Peak list of UV/Vis chromatogram for purity analysis of (*S*)-9s.

Apex RT	Start RT	End RT	Area	%Area	Height	%Height
2.82	2.78	2.95	49126.895	1.04	10493.928	2.11
3.38	3.25	3.75	4672597.072	98.96	486172.558	97.89

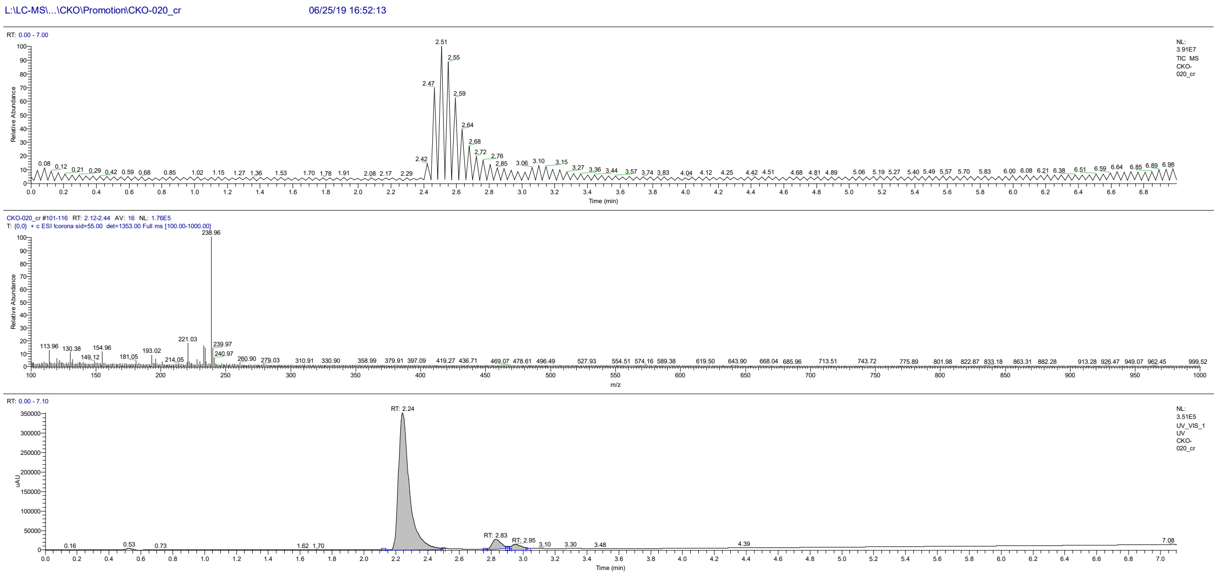

SB-Fig.32: LC-MS data of 12b (top: mass chromatogram, middle: mass spectrum of main UV/Vis peak, bottom: UV/Vis chromatogram).

SB-Table 5: Peak list of UV/Vis chromatogram for purity analysis of 12b.

Apex RT	Start RT	End RT	Area	%Area	Height	%Height
2.24	2.12	2.50	1678611.788	91.09	350247.991	90.11
2.83	2.76	2.90	94756.308	5.14	24123.759	6.21
2.95	2.91	3.03	69338.978	3.76	14315.320	3.68

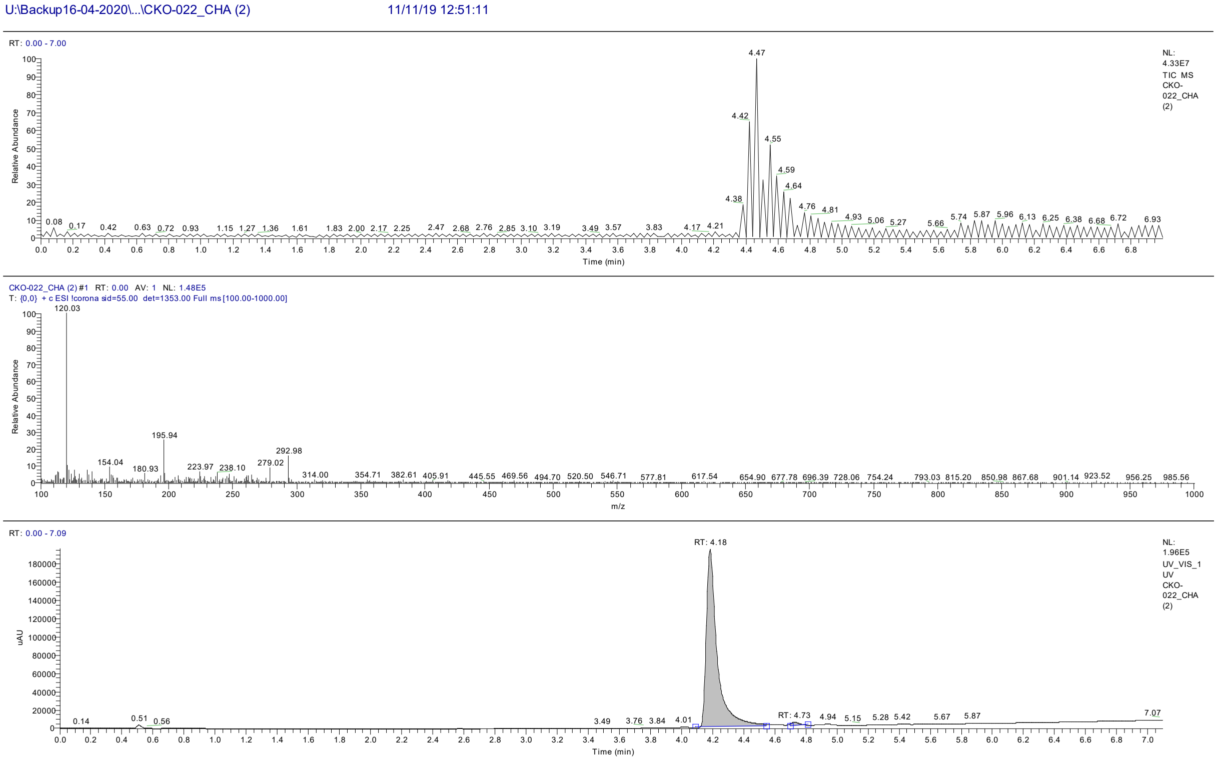

SB-Fig.33: LC-MS data of 12s (top: mass chromatogram, middle: mass spectrum of main UV/Vis peak, bottom: UV/Vis chromatogram).

SB-Table 6: Peak list of UV/Vis chromatogram for purity analysis of 12s.

Apex RT	Start RT	End RT	Area	%Area	Height	%Height
4.18	4.09	4.55	979004.637	98.34	195111.321	97.85
4.73	4.70	4.81	16529.825	1.66	4295.881	2.15

4.2.2 Purity analyses of commercial compounds

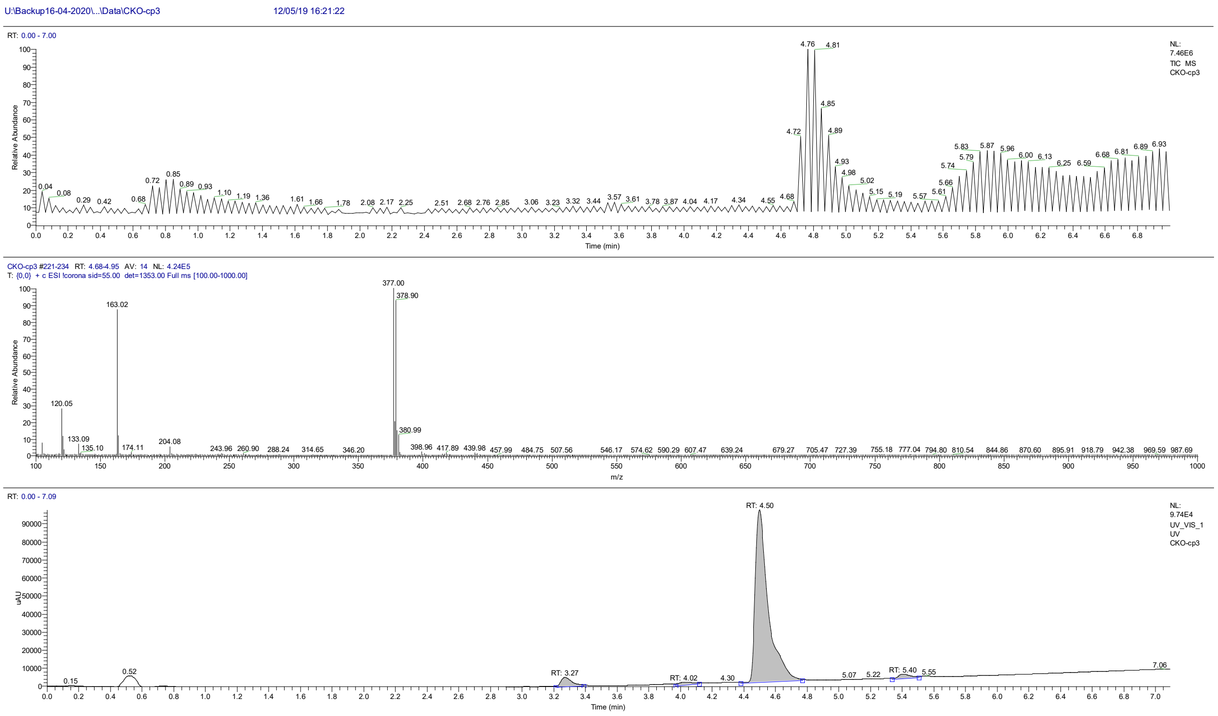

SB-Fig.34: LC MS data of Z15682900 (top: mass chromatogram, middle: mass spectrum of main UV/Vis peak, bottom: UV/Vis chromatogram).

SB-Table 7: Peak list of UV/Vis chromatogram for purity analysis of Z15682900.

Apex RT	Start RT	End RT	Area	%Area	Height	%Height
3.27	3.21	3.39	22131.608	3.85	4910.965	4.77
4.02	3.97	4.12	6018.691	1.05	1120.822	1.09
4.50	4.38	4.77	537914.502	93.60	94987.635	92.21
5.40	5.34	5.51	8618.649	1.50	1998.097	1.94

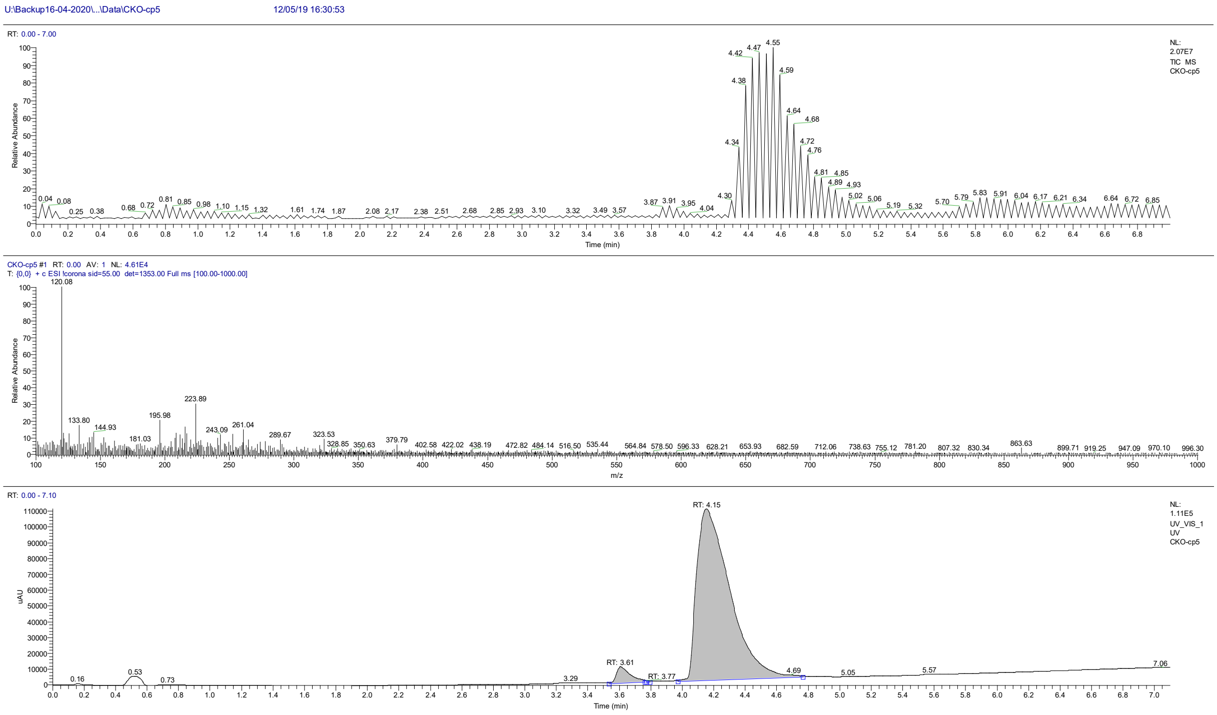

SB-Fig.35: LC-MS data of T6092069 (top: mass chromatogram, middle: mass spectrum of main UV/Vis peak, bottom: UV/Vis chromatogram).

SB-Table 8: Peak list of UV/Vis chromatogram for purity analysis of T6092069.

Apex RT	Start RT	End RT	Area	%Area	Height	%Height
3.61	3.55	3.81	59172.816	3.74	9831.828	8.41
4.15	4.03	4.66	1525044.130	96.26	107108.906	91.59

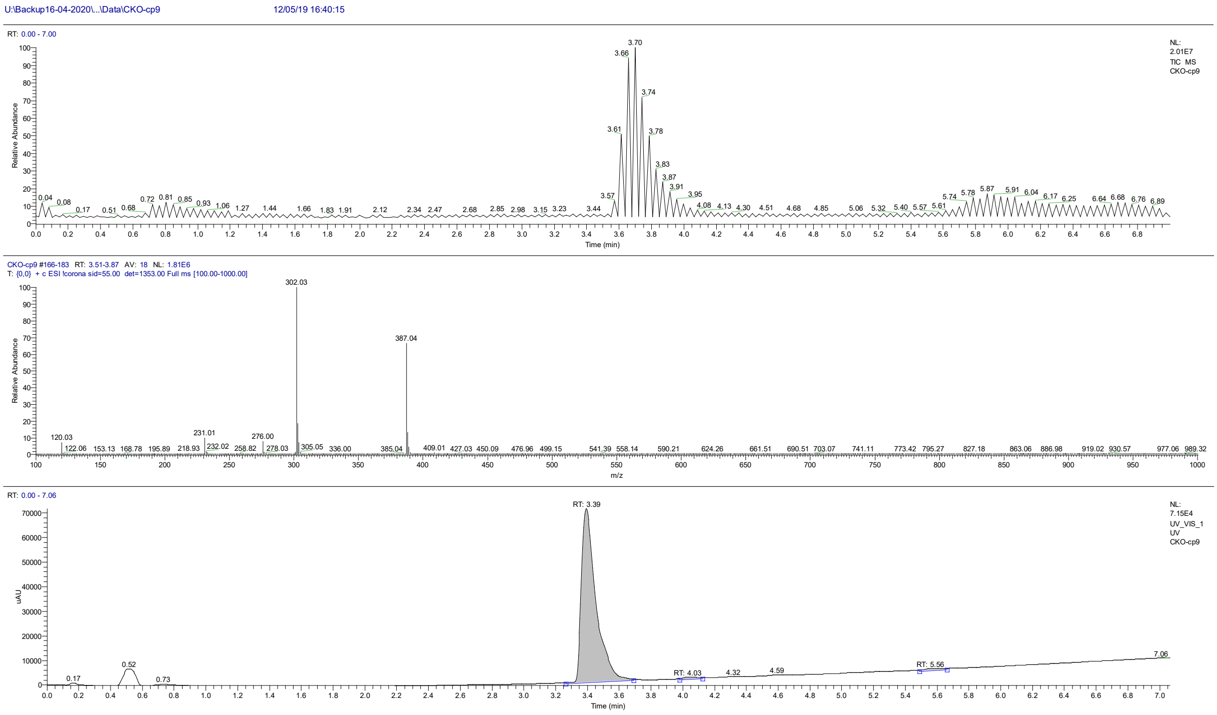

SB-Fig.36: LC-MS data of Z46486286. (top: mass chromatogram, middle: mass spectrum of main UV/Vis peak, bottom: UV/Vis chromatogram).

SB-Table 9: Peak list of UV/Vis chromatogram for purity analysis of Z46486286.

Apex RT	Start RT	End RT	Area	%Area	Height	%Height
3.39	3.26	3.69	446142.831	98.66	70256.712	98.45
4.03	3.98	4.12	3261.649	0.72	648.641	0.91
5.56	5.49	5.66	2816.670	0.62	457.245	0.64

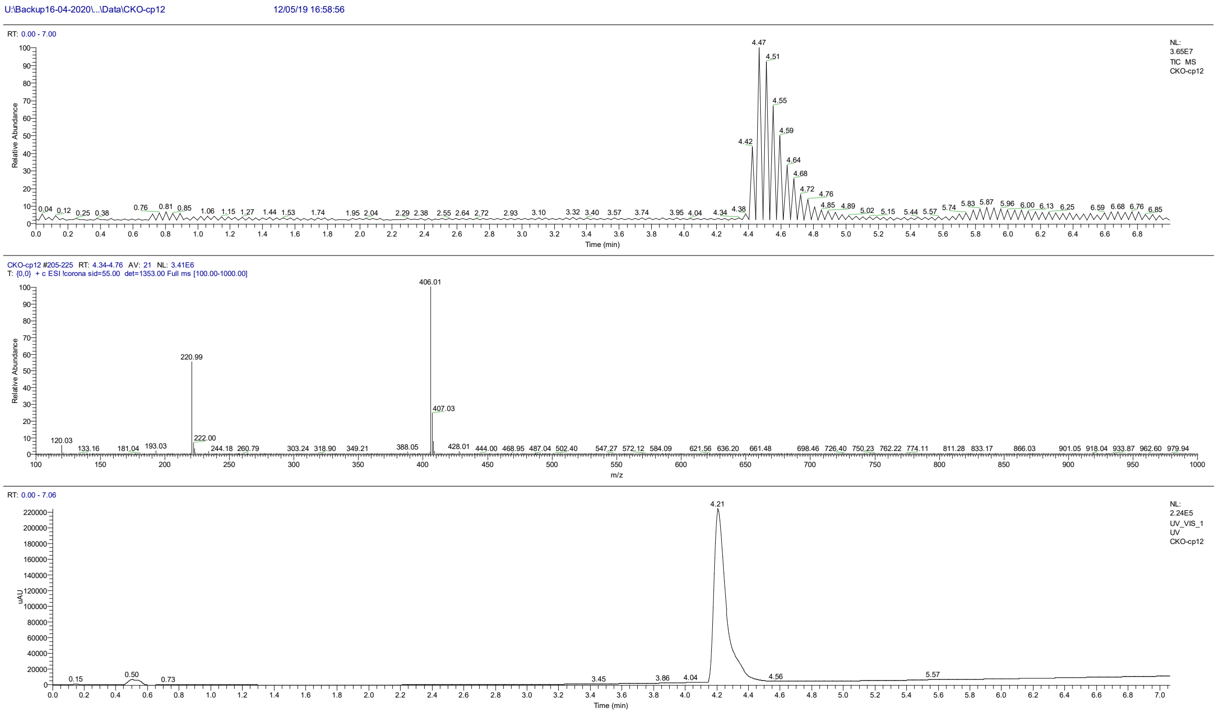

SB-Fig.37: LC-MS data of Z14490688. (top: mass chromatogram, middle: mass spectrum of UV/Vis peak, bottom: UV/Vis chromatogram).

4.3 HRMS data

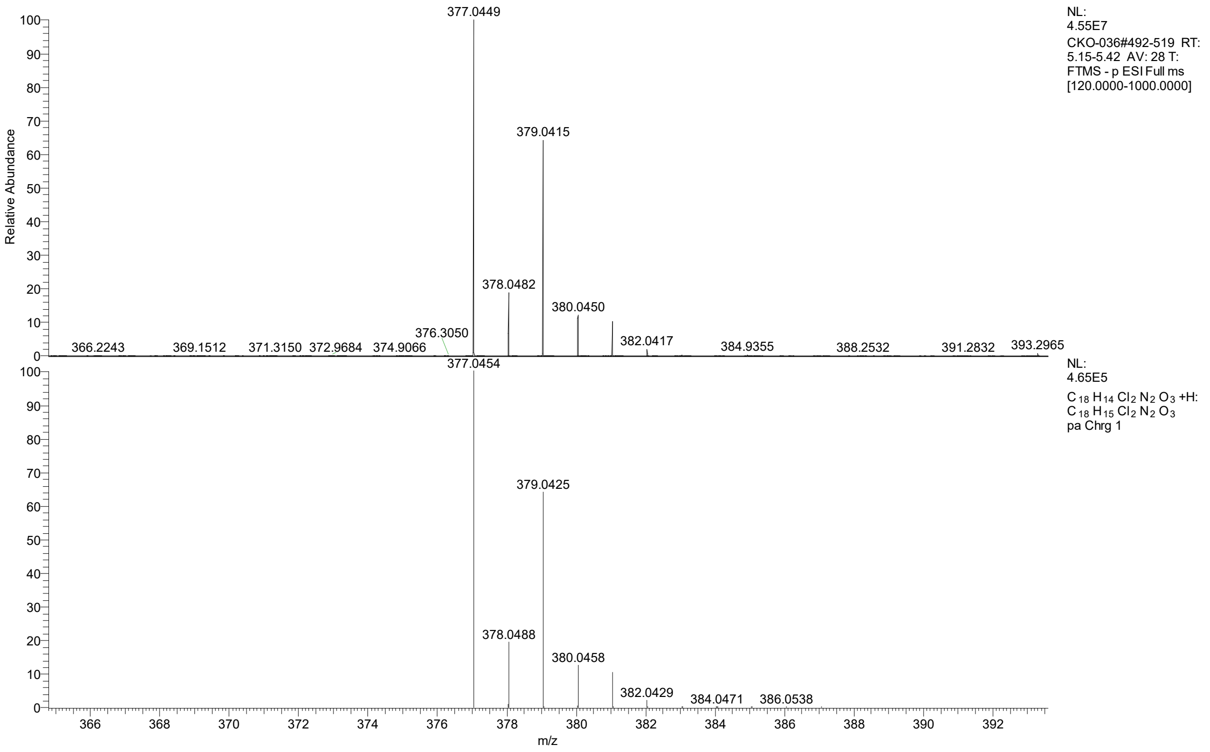

SB-Fig.38: HRMS spectra of 3s (top: measured spectrum, middle and bottom: calculated spectra).

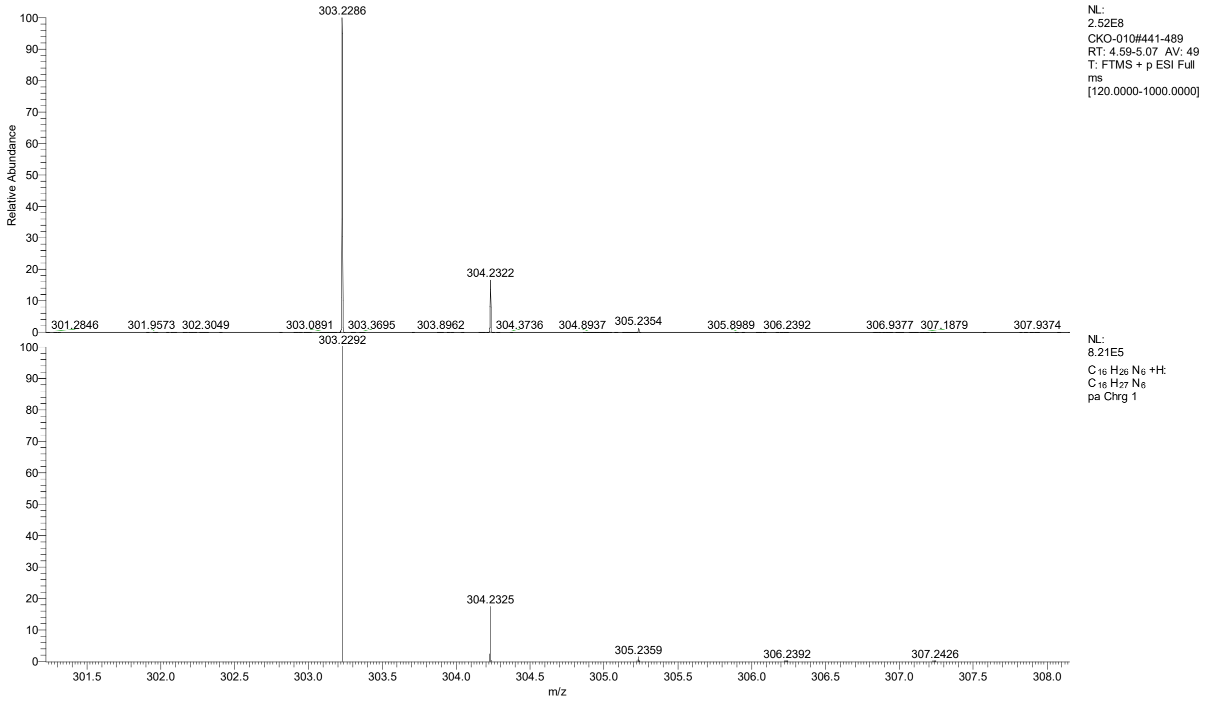

SB-Fig.39: HRMS spectra of 5s (top: measured spectrum, bottom: calculated spectrum).

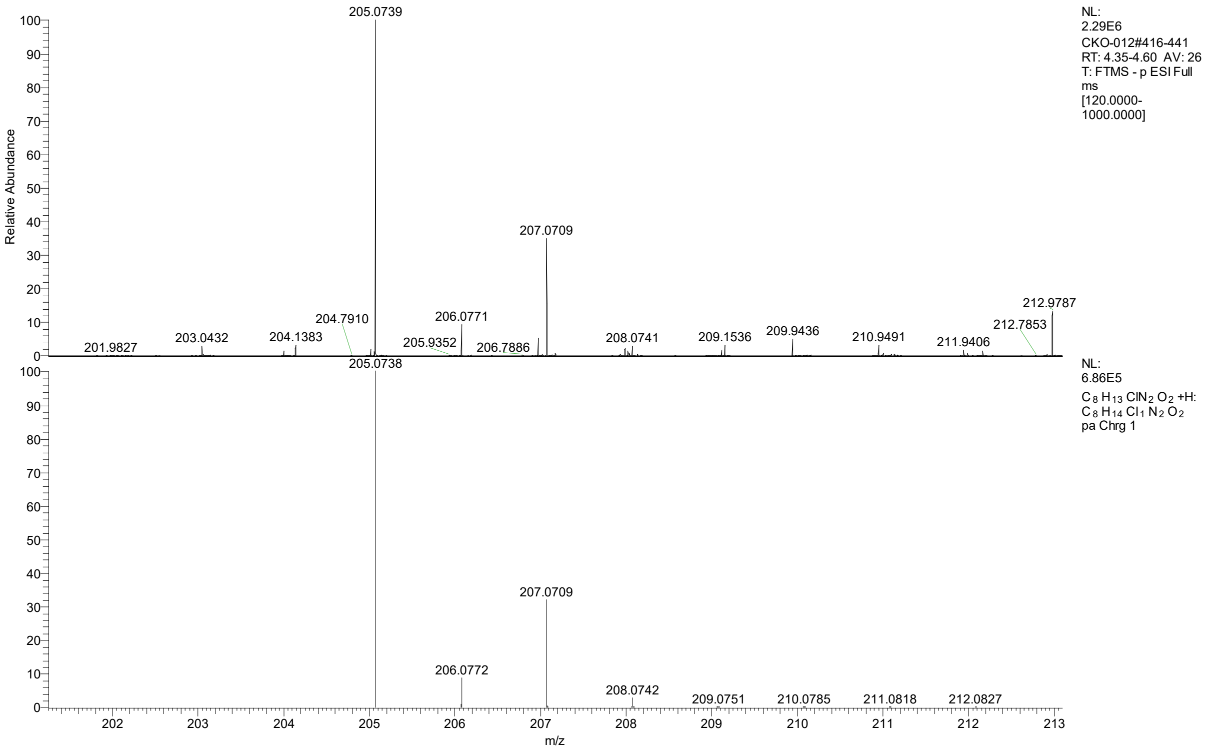

SB-Fig.40: HRMS spectra of 9d (top: measured spectrum, bottom: calculated spectrum).

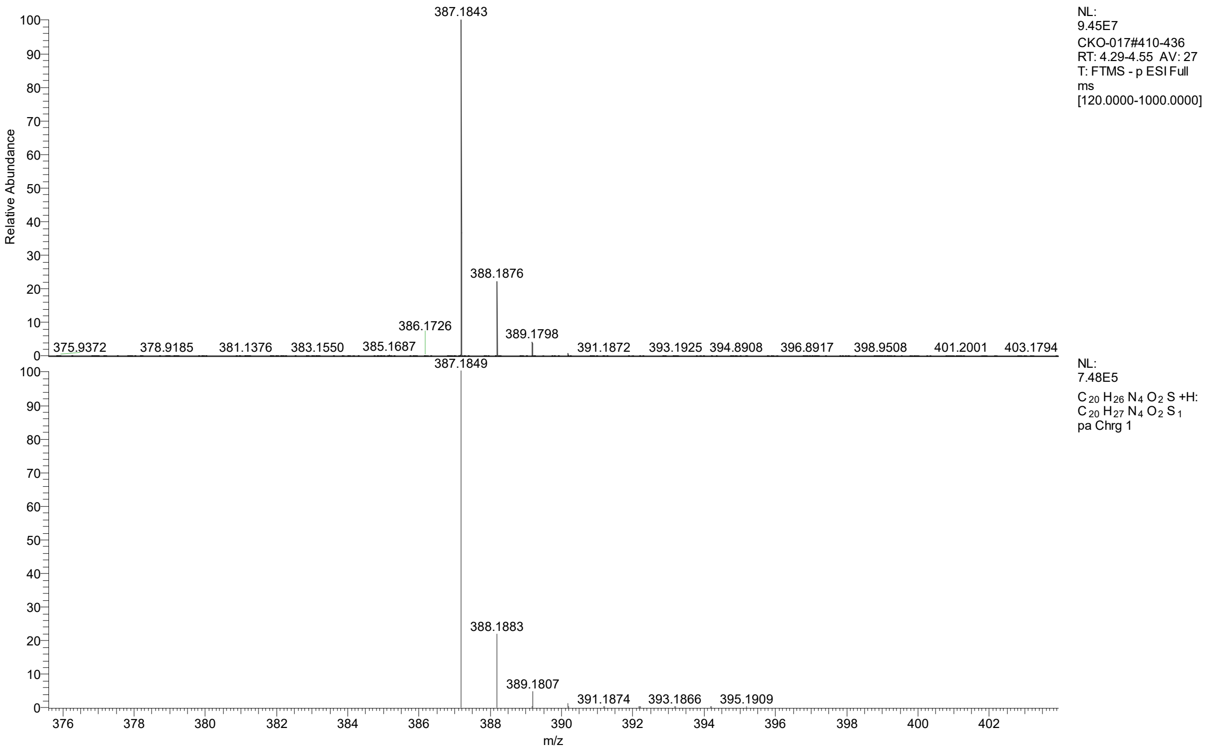

SB-Fig.41: HRMS spectra of (*R*)-9s (top: measured spectrum, middle and bottom: calculated spectra).

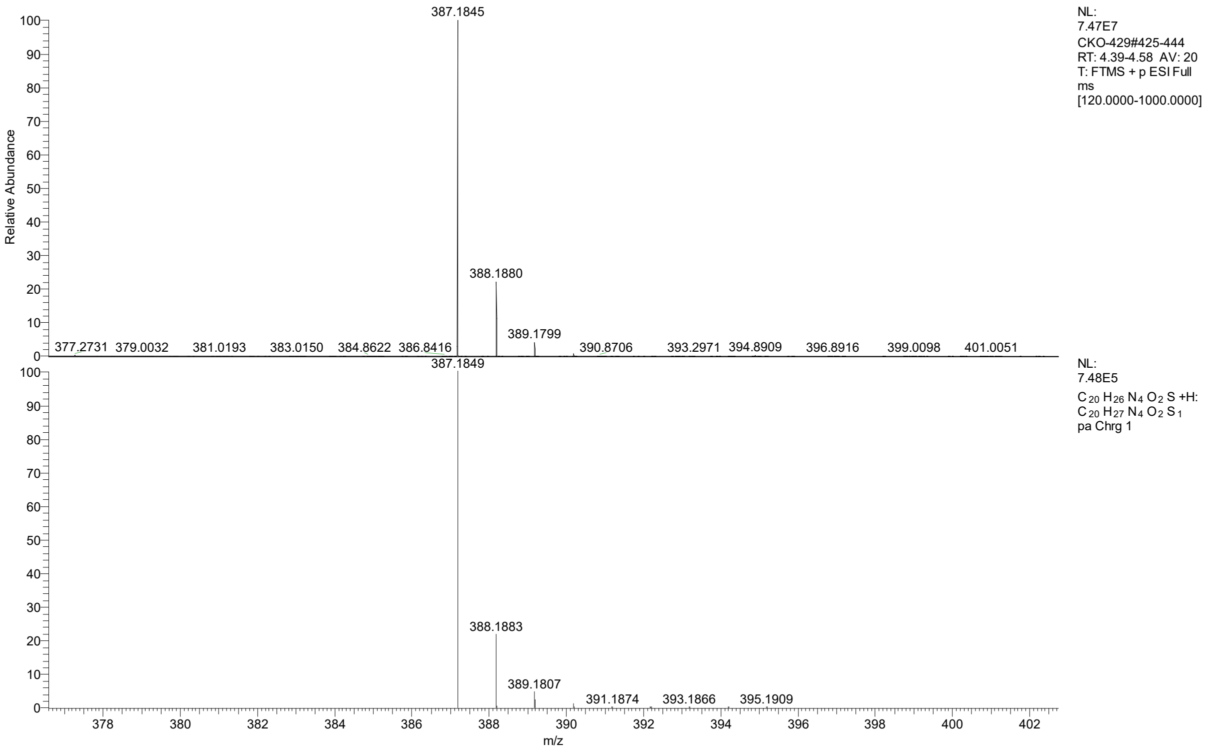

SB-Fig.42: HRMS spectra of (*S*)-9s (top: measured spectrum, middle and bottom: calculated spectra).

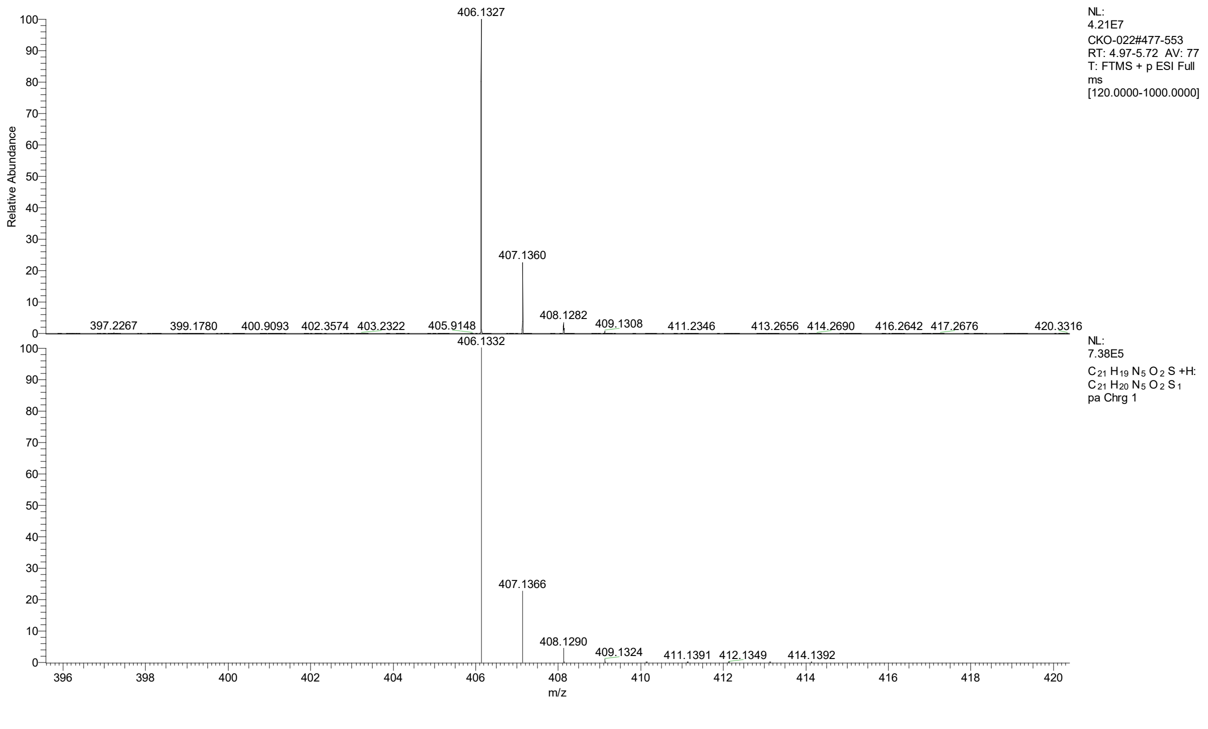

SB-Fig.43: HRMS spectra of 12s (top: measured spectrum, bottom: calculated spectrum).

5. List of Figures

SB-Fig.1: ^{1}H NMR spectrum of 3b in DMSO-d_6 at 500 megahertz (MHz). 13
SB-Fig.2: ^{13}C NMR spectrum of 3b in DMSO-d_6 at 126 MHz. 14
SB-Fig.3: ^{1}H NMR spectrum of 3s recorded in DMSO-d_6 at 500 MHz. 14
SB-Fig.4: ^{13}C NMR spectrum of 3s recorded in DMSO-d_6 at 126 MHz. 15
SB-Fig.5: HSQC-2D spectrum of 3s recorded in DMSO-d_6. 15
SB-Fig.6: ^{1}H NMR spectrum of 5b recorded in $CDCl_3$ at 500 MHz. 16
SB-Fig.7: ^{13}C NMR spectrum of 5b recorded in $CDCl_3$ at 126 MHz. 16
SB-Fig.8: ^{1}H NMR spectrum of 5s recorded in $CDCl_3$ at 500 MHz. 17
SB-Fig.9: ^{13}C NMR spectrum of 5s recorded in $CDCl_3$ at 126 MHz. 17
SB-Fig.10: ^{1}H NMR spectrum of 9b-1 recorded in $CDCl_3$ at 500 MHz. 18
SB-Fig.11: ^{13}C NMR spectrum of 9b-1 recorded in $CDCl_3$ at 126 MHz. 18
SB-Fig.12: ^{1}H NMR spectrum of 9b-2 recorded in $CDCl_3$ at 500 MHz. 19
SB-Fig.13: ^{13}C NMR of 9b-2 recorded in $CDCl_3$ at 126 MHz. 19
SB-Fig.14: ^{1}H NMR spectrum of 9d recorded in acetone-d_6 at 500 MHz. 20
SB-Fig.15: ^{13}C NMR spectrum of 9d recorded in acetone-d_6 at 126 MHz. 20
SB-Fig.16: ^{1}H NMR spectrum of (*R*)-9s recorded in $CDCl_3$ at 500 MHz. 21
SB-Fig.17: ^{13}C NMR spectrum of (*R*)-9s recorded in $CDCl_3$ at 126 MHz. 21
SB-Fig.18: ^{1}H NMR spectrum of (*S*)-9s recorded in DMSO-d_6 at 500 MHz. 22
SB-Fig.19: ^{13}C NMR spectrum of (*S*)-9s measured in DMSO-d_6 at 126 MHz. 22
SB-Fig.20: ^{1}H NMR spectrum of 12b measured in DMSO-d_6 at 500 MHz. 23
SB-Fig.21: ^{13}C NMR spectrum of 12b measured in DMSO-d_6 at 126 MHz. 23
SB-Fig.22: ^{1}H NMR spectrum of 12s measured in $CDCl_3$ at 500 MHz. 24
SB-Fig.23: ^{13}C NMR spectrum of 12s measured in $CDCl_3$ at 126 MHz. 24
SB-Fig.24: LC-MS data of 3b (top: mass chromatogram, middle: mass spectrum of UV/Vis peak, bottom: UV/Vis chromatogram). 25
SB-Fig.25: LC-MS data of 3s (top: mass chromatogram, middle: mass spectrum of UV/Vis peak, bottom: UV/Vis chromatogram). 26
SB-Fig.26: LC-MS data of 5b (top: mass chromatogram, middle: mass spectrum of UV/Vis peak, bottom: UV/Vis chromatogram). 26
SB-Fig.27: LC-MS data of 5s (top: mass chromatogram, middle: mass spectrum of main UV/Vis peak, bottom: UV/Vis chromatogram). 27
SB-Fig.28: LC-MS data of 9b-1 (top: mass chromatogram, middle: mass spectrum of main UV/Vis peak, bottom: UV/Vis chromatogram). 28
SB-Fig.29: LC-MS data of 9b-2 (top: mass chromatogram, middle: mass spectrum of UV/Vis peak, bottom: UV/Vis chromatogram). 29
SB-Fig.30: LC-MS data of (*R*)-9s (top: mass chromatogram, middle: mass spectrum of UV/Vis peak, bottom: UV/Vis chromatogram). 29
SB-Fig.31: LC-MS data of (*S*)-9s (top: mass chromatogram, middle: mass spectrum of main UV/Vis peak, bottom: UV/Vis chromatogram). 30
SB-Fig.32: LC-MS data of 12b (top: mass chromatogram, middle: mass spectrum of main UV/Vis peak, bottom: UV/Vis chromatogram). 30
SB-Fig.33: LC-MS data of 12s (top: mass chromatogram, middle: mass spectrum of main UV/Vis peak, bottom: UV/Vis chromatogram). 31
SB-Fig.34: LC MS data of Z15682900 (top: mass chromatogram, middle: mass spectrum of main UV/Vis peak, bottom: UV/Vis chromatogram). 32
SB-Fig.35: LC-MS data of T6092069 (top: mass chromatogram, middle: mass spectrum of main UV/Vis peak, bottom: UV/Vis chromatogram). 33
SB-Fig.36: LC-MS data of Z46486286. (top: mass chromatogram, middle: mass spectrum of main UV/Vis peak, bottom: UV/Vis chromatogram). 34
SB-Fig.37: LC-MS data of Z14490688. (top: mass chromatogram, middle: mass spectrum of UV/Vis peak, bottom: UV/Vis chromatogram). 35
SB-Fig.38: HRMS spectra of 3s (top: measured spectrum, middle and bottom: calculated spectra). 36
SB-Fig.39: HRMS spectra of 5s (top: measured spectrum, bottom: calculated spectrum). 36
SB-Fig.40: HRMS spectra of 9d (top: measured spectrum, bottom: calculated spectrum). 37
SB-Fig.41: HRMS spectra of (*R*)-9s (top: measured spectrum, middle and bottom: calculated spectra). 37

SB-Fig.42: HRMS spectra of (*S*)-9s (top: measured spectrum, middle and bottom: calculated spectra)............. 38
SB-Fig.43: HRMS spectra of 12s (top: measured spectrum, bottom: calculated spectrum). 38

6. List of Tables

SB-Table 1: Peak list of UV/Vis chromatogram for purity analysis of 3b.. 25
SB-Table 2: Peak list of UV/Vis chromatogram for purity analysis of 5s. 27
SB-Table 3: Peak list of UV/Vis chromatogram for purity analysis of 9b-1. 28
SB-Table 4: Peak list of UV/Vis chromatogram for purity analysis of (*S*)-9s. 30
SB-Table 5: Peak list of UV/Vis chromatogram for purity analysis of 12b.. 31
SB-Table 6: Peak list of UV/Vis chromatogram for purity analysis of 12s. 31
SB-Table 7: Peak list of UV/Vis chromatogram for purity analysis of Z15682900. 32
SB-Table 8: Peak list of UV/Vis chromatogram for purity analysis of T6092069. 33
SB-Table 9: Peak list of UV/Vis chromatogram for purity analysis of Z46486286. 34

7. Supplementary References

1. Cheng C, Liu Z, Shen L et al. Synthesis and spectral properties of triazine-based dendritic dithienylethenes. *Journal of Chemical Research* 2011; **35**: 282-7.
2. Harty RNF, Bruce D.; Wrobel, Jay E.; Reitz, Allen B.; Loughran, H. Marie Novel antiviral compounds and methods usinge same cross-reference to related applications., 2015.
3. Singh RB, Singh GK, Chaturvedi K et al. Design, synthesis, characterization, and molecular modeling studies of novel oxadiazole derivatives of nipecotic acid as potential anticonvulsant and antidepressant agents. *Medicinal Chemistry Research* 2018; **27**: 137-52.
4. Orfi L, Waczek F, Pato J et al. Improved, High Yield Synthesis of 3H-Quinazolin-4-ones, the Key Intermediates of Recently Developed Drugs. *Current Medicinal Chemistry* 2004; **11**: 2549-53.
5. Li F, Feng Y, Meng Q et al. An efficient construction of quinazolin-4 (3H)-ones under microwave irradiation. *Arkivoc* 2007; **1**: 40-50.
6. Lee NK, Lee, Jun Won, Lee, Sukho, Im, Guang-jin, Han, Hye Young, Kim, Tae Kon, Kim, Yong Hyuk, Kwak, Wie-jong, Kim, Sang Woong, Ha, Joohun, Kim, Eon Kyum, Lee, Jung Kyu, Yoo, Choong Yeul, Lee, Dae Yeon. Quinazoline derivatives for the treatment and prevention of diabetes and obesity. United States: SK CHEMICALS CO., LTD. (Suwon-si, KR),LEADGENEX INC. (Daejeon, KR),INDUSTRY ACADEMIC COOPERATION FOUNDATION OF KYUNGHEE UNIVERSITY (Yongsin-si, KR), 2008.
7. Fortin JS, Lacroix J, Desjardins M et al. Alkylation potency and protein specificity of aromatic urea derivatives and bioisosteres as potential irreversible antagonists of the colchicine-binding site. *Bioorg Med Chem* 2007; **15**: 4456-69.
8. Yang G, Xu L, Lu A. Synthesis and bioactivity of novel triazolo [1, 5-a] pyrimidine derivatives [3]. *Heteroatom Chemistry: An International Journal of Main Group Elements* 2001; **12**: 491-6.
